# Causal association of plasma lipidome with lung carcinoma and mediating role of inflammatory proteins: evidence from Mendelian randomization analysis

**DOI:** 10.7150/jca.99990

**Published:** 2024-09-03

**Authors:** Haihao Yan, Jiao Feng, Xiao Jin, Yuanyuan Zhang, Cui Bao, Chenghua Zhu, Ganzhu Feng

**Affiliations:** 1 Department of Respiratory and Critical Care Medicine, The Second Affiliated Hospital of Nanjing Medical University, Nanjing 210011, China.; 2 Department of Respiratory Medicine, Nanjing Pukou Hospital of TCM, Pukou Hospital of Chinese Medicine affiliated to China Pharmaceutical University, Nanjing 210000, China.

**Keywords:** plasma lipidome, inflammatory proteins, lung carcinoma, Mendelian randomization

## Abstract

The evidence from clinical studies suggests that lung carcinoma (LC) patients exhibit dysregulation in lipid metabolism. However, the causal relationship between plasma lipidome and LC, and whether inflammatory proteins mediate, remains to be determined. Genetic data for 179 plasma lipids and 91 inflammatory proteins were obtained from the latest published genome-wide association studies. Genetic data on LC and subtypes were from the largest available meta-analysis. The causal relationship between plasma lipidome and LC was determined by the two-sample Mendelian randomization (MR) method. Mediation MR analysis was employed to ascertain whether inflammatory proteins mediate the impact of plasma lipidome on LC. We identified 39 causal relationships between genetically predicted plasma lipidome and LC and subtypes. These relationships involve the influence of phosphatidylcholines, phosphatidylethanolamines, diacylglycerols, triacylglycerols, sphingomyelins, and Sterol esters. Additionally, the mediating role of 5 inflammatory proteins in the causal relationship between plasma lipidome and LC and subtypes was determined. Our results highlight the complex network of plasma lipidome and inflammatory proteins regulating LC. Integrating plasma lipidome and inflammatory proteins into clinical practice may open new avenues for the prevention and treatment of LC.

## Introduction

Lung carcinoma (LC) is a highly lethal systemic invasive disease often associated with systemic metabolic dysregulation. Currently, LC remains a leading cause of cancer-related deaths globally, ranking second in incidence among all cancers 1.

Lipids are a highly diverse class of molecules, including phosphatidylcholines (PC), phosphatidylethanolamines (PE), diacylglycerols (DAG), triacylglycerols (TAG), sphingomyelins (SM), phosphatidylinositols (PI), cholesterol esters (CE), and sterol esters (SE). Lipids regulate and perform various biological functions, such as energy storage, maintenance of normal cell structure, involvement in cell signaling pathways, and regulation of inflammatory responses 2. Many studies have confirmed the presence of lipid metabolism dysregulation in LC patients. Circulating levels of PC in early-stage non-small cell lung carcinoma (NSCLC) patients show significant differences compared to healthy control groups 3. Heterogeneity in circulating lipidomic profiles has also been observed among patients with different subtypes of LC, such as lung adenocarcinoma (LADC), squamous cell carcinoma (SqCLC), or small cell lung carcinoma (SCLC) 4. Moreover, a significant correlation exists between the lipidomic profiles and the genome of lipid-associated proteins 4.

One of the key roles of lipids in maintaining metabolic homeostasis is regulating inflammatory responses. Different lipids may either promote or attenuate the inflammatory process (such as prostaglandins, leukotrienes, and endocannabinoid signaling). Observational evidence suggests that chronic inflammation is associated with the development of LC 5, 6. Prospective cohort studies indicate that specific circulating inflammatory proteins, such as Interleukin (IL)-6, IL-8, and C-reactive protein (CRP), are associated with an increased LC risk 5, 7, 8. Elevated serum concentrations of monokine induced by gamma interferon (MIG) and serum amyloid A (SAA) prior to diagnosis are also positively correlated with the risk of LC 9.

The evidence of the above observational studies suggests that lipids and inflammatory proteins may be associated with the LC risk. Inflammatory protein may be the mediating factor of lipids affecting LC. However, traditional observational designs are susceptible to residual confounding and reverse causality. Even with appropriate statistical methods, the impact of these biases remains unavoidable. Therefore, translating the results of observational studies into practical cancer control strategies still presents significant challenges.

Mendelian Randomization (MR) utilizes germline genetic variants as genetic instruments to proxy lifelong exposure to risk factors (such as lipids and inflammatory proteins) 10, 11. One of the advantages of MR is its ability to overcome the limitations of traditional epidemiology, thereby strengthening the evidence for potential causal effects of risk factors on cancer risk. Another advantage of MR is its consideration of the long-term effects of risk factors on cancer risk, as there may be a considerable exposure period from exposure to specific risk factors to the occurrence of cancer.

This study used an MR design to explore the causal relationship between plasma lipidome and LC and subtypes, including LADC, SqCLC, and SCLC. Mediated MR was used to evaluate whether inflammatory proteins serve as mediators of lipids affecting LC. Our study will expand insights into the impact of lipids and inflammatory proteins on the etiology of LC and support the development of LC prevention strategies.

## Materials and methods

### Study design

**Figure 1:**
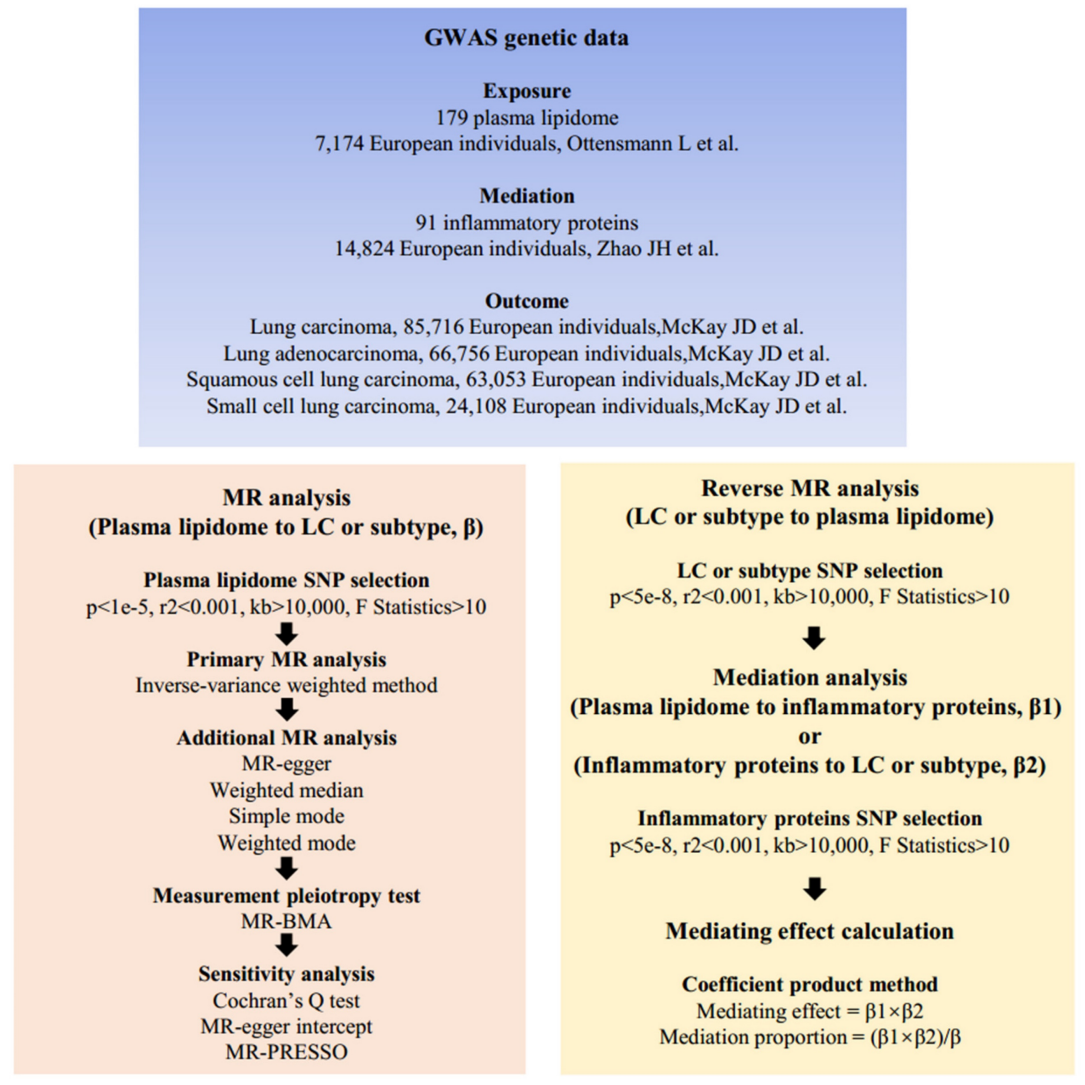
Mendelian randomization analysis flow chart. β, the total effect of lipids on LC, obtained using the IVW method, is denoted as β; β1, the effect of lipids on inflammatory proteins is denoted as β1; β2, the effect of inflammatory proteins on LC is denoted as β2. Abbreviations: SNPs, single nucleotide polymorphisms; GWAS, genome-wide association studies; MR, mendelian randomization; MR-PRESSP, the MR Pleiotropy RESidual Sum and Outlier.

Our study meets the STROBE-MR criteria 12 (**Supplementary Table S1**) and consists of three main parts. Firstly, we determined whether 179 plasma lipids are associated with the risk of LC and subtypes. Secondly, we analyzed whether LC has a reverse causal effect on plasma lipidome. Finally, mediation MR analysis was conducted to ascertain whether inflammatory proteins mediate the causal relationship between plasma lipidome and LC. The overall design of this study is illustrated in **Figure 1**.

### Plasma lipidome

The genetic data for 179 plasma lipids were obtained from the latest genome-wide association studies (GWAS) based on the GeneRISK cohort. This cohort recruited 7,266 participants from southern Finland between 2015 and 2017, including 2,624 males and 4,642 females aged 45 to 66 years old. Blood samples were collected from fasting participants for serum, plasma, and DNA extraction. The lipidomic analysis based on mass spectrometry was conducted by Linotype GmbH (Dresden, Germany) to analyze the lipids of the participants. Ultimately, this study identified 495 genome-wide associations of plasma lipids at 56 genetic loci 13.

### Inflammatory proteins

The genetic data for 91 inflammatory proteins were obtained from the latest GWAS conducted by Zhao et al. This study recruited 11 cohorts comprising a total of 14,824 participants of European ancestry. Protein profiling data for each cohort were generated by the Olink laboratory in Uppsala. The researchers used the Olink Target platform to measure 91 plasma proteins and performed genome-wide protein quantitative trait locus (pQTL) mapping and meta-analysis. A total of 180 pQTLs were identified (59 cis, 121 trans). This GWAS identified genetic determinants of inflammation-related proteins, which can aid in further understanding the etiology of immune-mediated LC 14.

### LC and subtypes

The GWAS related to LC was derived from a meta-analysis based on the International Lung Carcinoma Consortium (ILCCO) and the Transdisciplinary Research in Cancer of the Lung (TRICL) consortium. It included a total of 85,716 participants of European ancestry, comprising 29,266 patients and 56,450 controls. Additionally, the histological types were further categorized into LADC, SqCLC, and SCLC. **Supplementary Table S2** displayed all genetic data.

### Screening genetic instruments

Genetic instruments for MR analysis need to meet three criteria: (1) they should be strongly associated with the exposure; (2) they should be unrelated to potential confounding variables; (3) they should only affect the outcome through the exposure. To obtain enough single nucleotide polymorphisms (SNPs) for MR analysis, for plasma lipidome and inflammatory proteins, we selected SNPs with p<1E-5. This threshold is also used in many MR studies 15, 16. Then, genetic instruments in linkage disequilibrium (LD) were further excluded. Subsequent SNPs used for MR should meet the criteria of r2<0.001 and distance >10,000kb. Additionally, the F statistic corresponding to each SNP was calculated to exclude weak genetic instrument bias. SNPs with F statistics less than 10 were further excluded. Finally, we excluded palindromic SNPs to ensure consistent effects of genetic instruments on exposure and outcomes. The plasma lipidome, inflammatory proteins, and LC-associated SNPs were presented in **Supplementary Tables S3, S4, and S5**, respectively.

### Forward MR analysis

In the primary analysis, the inverse variance-weighted (IVW) method was employed to assess the causal effects of 179 plasma lipids on LC and subtypes. Using the Wald ratio method, the individual impact of each genetic instrument on the outcome was calculated. The IVW method conducts a meta-analysis of the Wald ratios corresponding to each genetic instrument, and the results are presented as odds ratios (OR) and 95% confidence intervals (CI). The robustness of the IVW method was assessed using the MR-Egger, Weighted Median, Simple Mode, and Weighted Mode methods. These MR methods have been described in our previous study 17. Due to the high correlation among the characteristics of plasma lipidome and inflammatory proteins involved in this study, which is based on a shared large number of SNPs, to correct for the "measurement pleiotropy" effect, we introduced the MR Bayesian model averaging (MR-BMA) method. The MR-BMA method is more suitable for detecting the effects of related biomarkers acting through the same causal pathway 18. Statistical significance is considered only when the IVW method is consistent with other MR methods and when both the IVW and MR-BMA methods yield p-values less than 0.05.

### Reverse MR analysis

Because mediation MR analysis requires excluding the influence of reverse causality, we tested the impact of LC and subtypes on changes in plasma lipidome. For LC and subtypes, we selected SNPs with p < 5E-8 for MR analysis. As mentioned earlier, we removed SNPs in LD and calculated the F-statistic. The methods used for MR analysis were consistent with those used in forward MR analysis.

### Sensitivity analysis

Cochran's Q test was used to assess the heterogeneity among the genetic instruments corresponding to each exposure 19. MR-Egger regression and MR-PRESSO were employed to detect whether MR analysis was influenced by potential horizontal pleiotropy 20, 21. Results with heterogeneity or horizontal pleiotropy were further excluded.

### Mediation MR analysis

The lipid for mediation analysis needs to meet the following conditions: (1) The lipid has a causal effect on LC or subtypes; (2) LC or subtypes do not have a reverse causal effect on the lipid. For lipids that meet these conditions, we first detect the causal effect of specific lipids on 91 inflammatory proteins. Next, we will determine the causal relationship between inflammatory proteins significantly influenced by specific lipids and LC or subtypes. Once the inflammatory proteins acting as mediators are identified, we will further calculate the direct effect of lipids on LC or subtypes, the indirect effect (mediation effect) mediated by inflammatory proteins, and the proportion of the effect of lipids on LC or subtypes explained by inflammatory proteins. We use the Coefficient product method for mediation analysis 22, 23. The total effect of lipids on LC, obtained using the IVW method, is denoted as β. The effect of lipids on inflammatory proteins is denoted as β1, and the effect of inflammatory proteins on LC is denoted as β2. Here, the mediation effect exerted by inflammatory proteins is calculated as β1×β2. The direct effect is the effect of exposure on the outcome after excluding the influence of the mediator, calculated as β - β1×β2. The proportion of the mediation effect to the total effect is calculated as (β1×β2)/β. All analyses in this study were conducted in R (version 4.3.2). The R packages used include the "TwoSampleMR" package 24 and the "MR-PRESSO" package 21.

## Results

### Causal effects of plasma lipidome on LC and subtypes

#### LC

**Figure 2:**
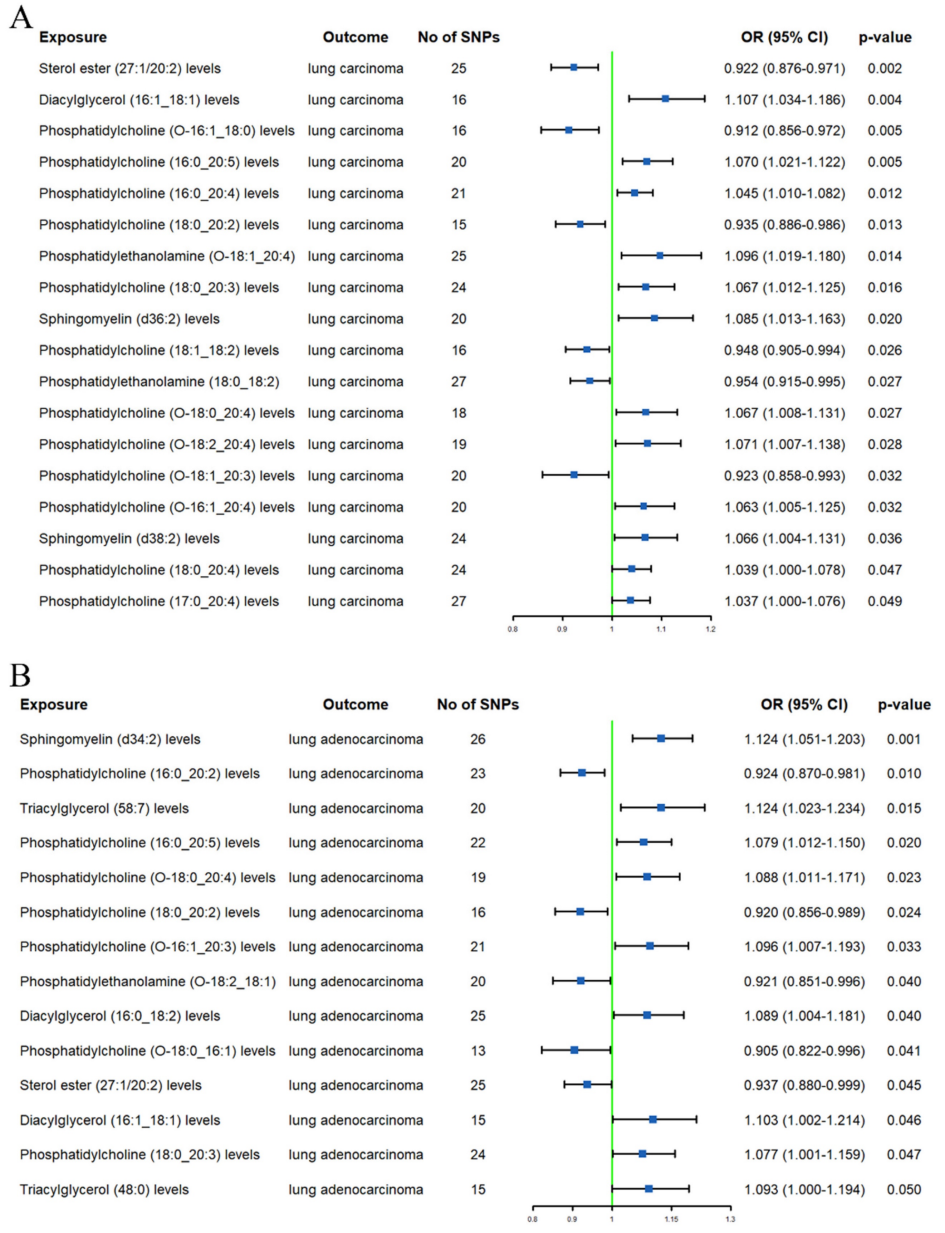
Forest plots of causal effect of plasma lipidome on lung carcinoma (A) and lung adenocarcinoma (B).

In the initial analysis, a total of 19 lipids were identified to have a causal relationship with LC (**Supplementary Table S6**). After using the MR-BMA method to remove lipids with non-significant results (p ≥ 0.05) (**Supplementary Table S7**), there were still 12 lipids associated with LC (**Figure 2A**). Specifically, an increase in genetically predicted SM (d36:2) levels (OR 1.085, 95% CI 1.013-1.163) and DAG (16:1_18:1) levels (OR 1.107, 95% CI 1.034-1.186) was associated with an increased risk of LC. However, an increase in SE (27:1/20:2) levels (OR 0.922, 95% CI 0.876-0.971) was associated with a decreased risk of LC. Additionally, PC (O-16:1_18:0), PC (18:0_20:2), PC (18:1_18:2), and PC (O-18:1_20:3) levels were found to be associated with a decreased LC risk (ORs = 0.912, 0.935, 0.948, and 0.923, respectively). In contrast, PC (16:0_20:5), PC (O-18:2_20:4), PC (16:0_20:4) levels, PC (O-16:1_20:4), and PC (17:0_20:4) levels increased the risk of LC (ORs = 1.070, 1.071, 1.045, 1.063, and 1.037, respectively). Additional MR methods (MR-Egger, Weighted Median, Simple Mode, and Weighted Mode) yielded consistent results with the IVW method, further confirming the robustness of our findings.

#### LADC

In the preliminary analysis, 14 lipids were found to be associated with LADC (**Supplementary Table S6**). After excluding lipids that did not pass the MR-BMA test (**Supplementary Table S7**), we finally identified 10 lipids (**Figure 2B**). The IVW method revealed that high standard deviation (SD) of TAG (48:0) (OR 1.093, 95% CI 1.000-1.194), TAG (58:7) (OR 1.124, 95% CI 1.023-1.234), DAG (16:0_18:2) (OR 1.089, 95% CI 1.004-1.181), and DAG (16:1_18:1) levels (OR 1.103, 95% CI 1.002-1.214) were associated with higher LADC risk. A high SD of SE (27:1/20:2) levels (OR 0.937, 95% CI 0.880-0.999) was associated with lower LADC risk. We also observed that higher PC (16:0_20:5) and PC (O-16:1_20:3) levels were associated with increased LADC risk (ORs = 1.079 and 1.096, respectively); however, higher PC (18:0_20:2) and PC (16:0_20:2) levels were associated with decreased LADC risk (ORs = 0.920 and 0.924, respectively). Additionally, a high SD of PE (O-18:2_18:1) levels (OR 0.921, 95% CI 0.851-0.996) was also associated with lower LADC risk. Furthermore, four additional MR analyses showed consistent directions with the main method.

#### SqCLC

**Figure 3:**
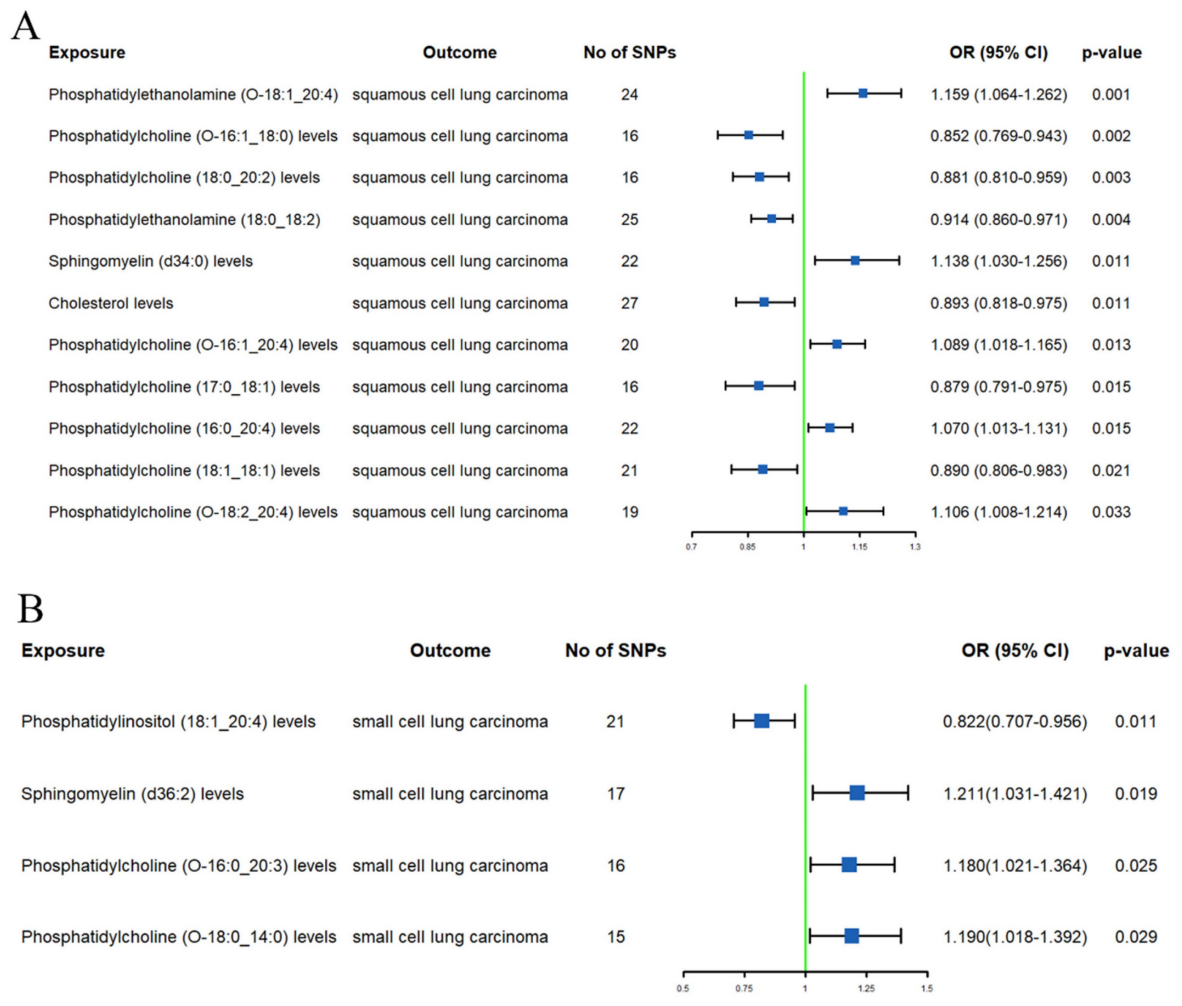
Forest plots of causal effect of plasma lipidome on squamous cell lung carcinoma (A) and small cell lung carcinoma (B).

After excluding 1 lipid using MR-BMA (**Supplementary Table S7**), we identified 14 lipids with a causal relationship with SqCLC (**Supplementary Table S6 and Figure 3A**). High genetically predicted SM (d34:0) levels (OR 1.138, 95% CI 1.030-1.256) were associated with an increased SqCLC risk. Conversely, a high PE (O-18:1_20:4) (OR 0.822, 95% CI 0.707-0.956) and PE (18:0_18:2) (OR 0.914, 95% CI 0.860-0.971) levels were associated with a low SqCLC risk. Additionally, PC (O-16:1_20:4), PC (16:0_20:4), and PC (O-18:2_20:4) levels were associated with an increased SqCLC risk (ORs = 1.089, 1.070, and 1.106, respectively). A high SD of PC (18:0_20:2), PC (O-16:1_18:0), PC (18:1_18:1), and PC (17:0_18:1) levels were associated with a decreased SqCLC risk (ORs = 0.881, 0.852, 0.890, and 0.879, respectively). It is noteworthy that although the IVW method suggested associations of SM (d38:2), SM (d36:1), PC (O-16:0_18:1), and PC (18:0_20:4) levels with SqCLC risk, additional MR analyses showed directions opposite to the primary method (**Supplementary Table S6**).

#### SCLC

After excluding 4 lipids using the MR-BMA method (**Supplementary Table S7**), we found only 4 lipids associated with SCLC risk (**Supplementary Table S6 and Figure 3B**). Specifically, a high SD of SM (d36:2) (OR 1.211, 95% CI 1.031-1.421), PC (O-16:0_20:3) (OR 1.180, 95% CI 1.021-1.364), and PC (O-18:0_14:0) levels (OR 1.190, 95% CI 1.018-1.392) were associated with an increased SCLC risk. PI (18:1_20:4) levels (OR 0.822, 95% CI 0.707-0.956) were associated with lower SCLC risk. The results of additional MR analyses were consistent with those of the IVW method in terms of direction (**Supplementary Table S6**).

### Reverse causal effects of LC and subtypes on plasma lipidome

To assess potential reverse causality effects, we used LC and its subtypes as exposures and plasma lipidome as outcomes. Based on the IVW method analysis, we only found potential causal effects of an increased risk of LADC (OR 0.934, 95% CI 0.873-1.000) with decreased PC (16:0_20:2) levels. No other evidence was found for the causal effects of LC and subtypes on plasma lipidome (**Supplementary Table S8**).

### MR sensitivity analysis

Cochran's Q test revealed heterogeneity among the genetic instruments for PC (O-16:1_20:4), PC (O-18:1_20:3), TAG (58:7), DAG (16:0_18:2), and SM (d36:1) levels (**Supplementary Table S9**). MR-Egger regression indicated that the causal effect of PC (O-16:1_20:3) on LADC was influenced by horizontal pleiotropy (**Supplementary Table S9**). Additionally, MR-PRESSO detected outliers among the genetic instruments for PC (O-16:1_20:4) and SM (d36:1) levels (**Supplementary Table S9**).

### Mediation effect of inflammation proteins

The initial mediation MR analysis identified 8 inflammation proteins that are simultaneously associated with both plasma lipidome and LC or subtypes. However, causal analyses for the effects of plasma lipidome on inflammation proteins (**Supplementary Table S10**) or inflammation proteins on LC or subtypes (**Supplementary Table 11**) showed that the additional MR analysis results for protein S100-A12, C-X-C motif chemokine 10, monocyte chemoattractant protein-3, and C-C motif chemokine 4 levels were inconsistent with the main analysis direction. Sensitivity analysis also revealed heterogeneity in the effect of C-X-C motif chemokine 10 levels on LC, and the causal relationship between C-C motif chemokine 4 levels and SqCLC was influenced by horizontal pleiotropy (**Supplementary Table S9**).

**Figure 4:**
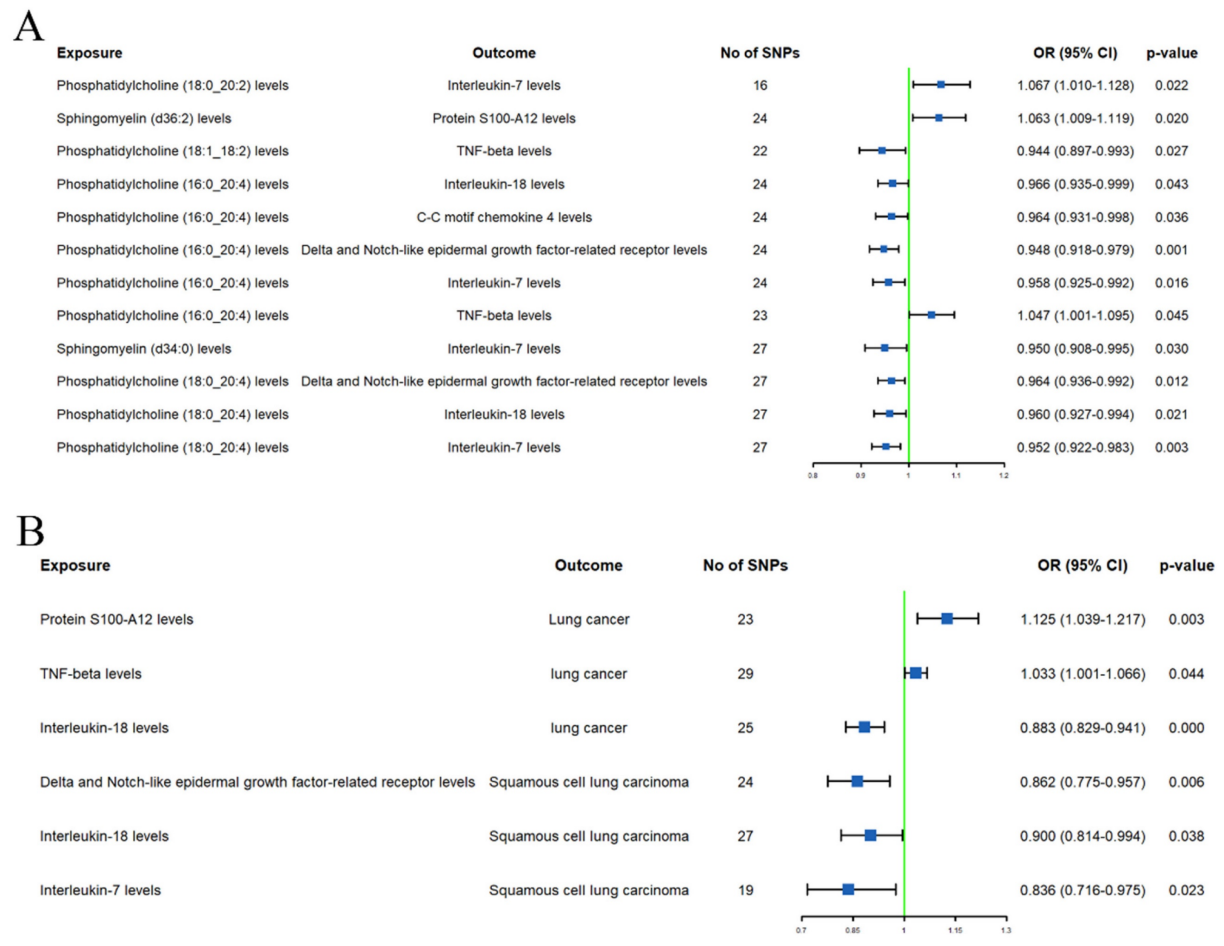
Forest plots of causal effects of plasma lipidome on inflammatory proteins (A) and causal effects of inflammatory proteins on lung carcinoma or subtypes (B).

**Figure 5:**
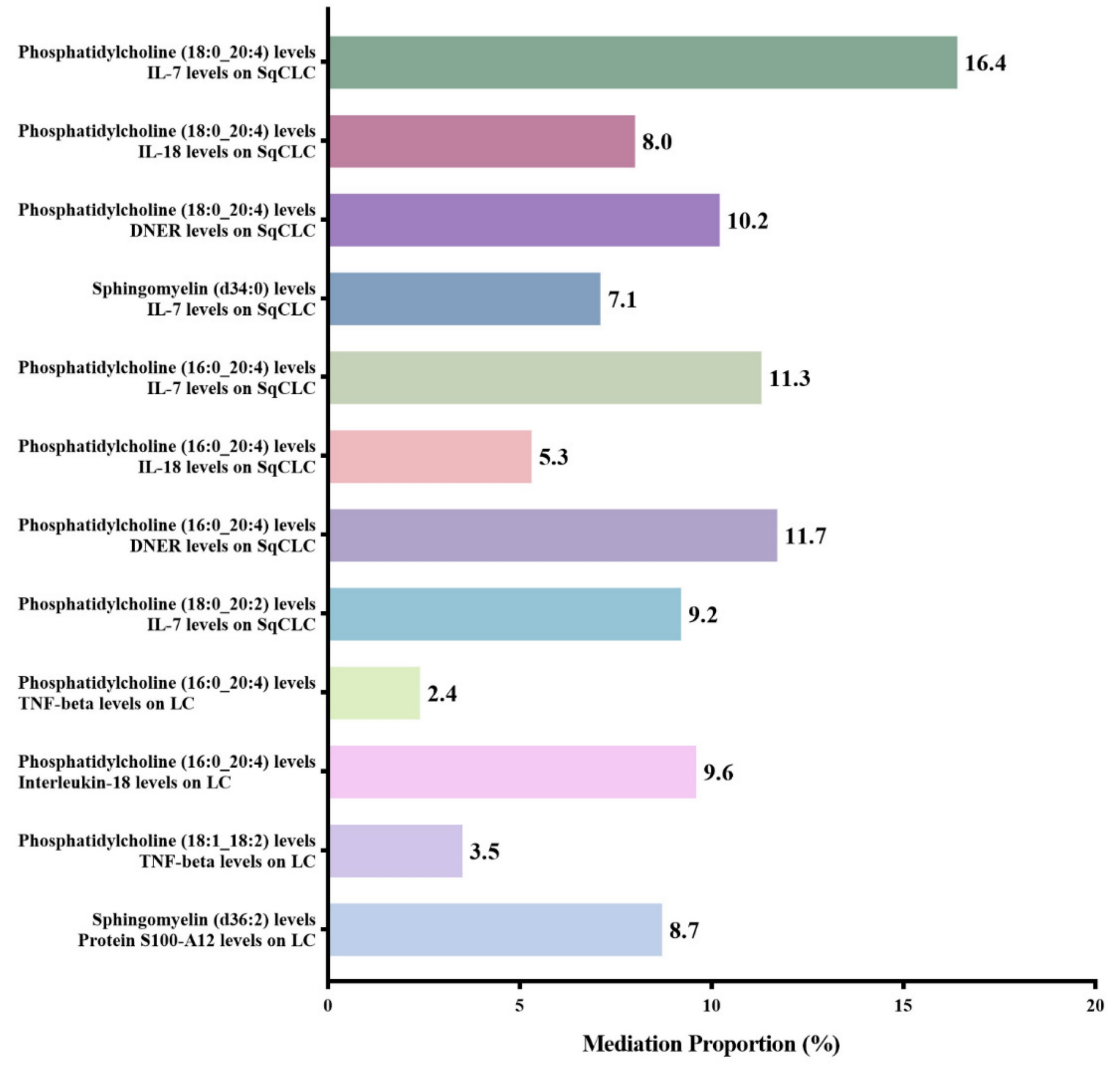
Mediation analysis of inflammatory proteins between plasma lipidome and lung carcinoma or subtypes.

**Figure 6:**
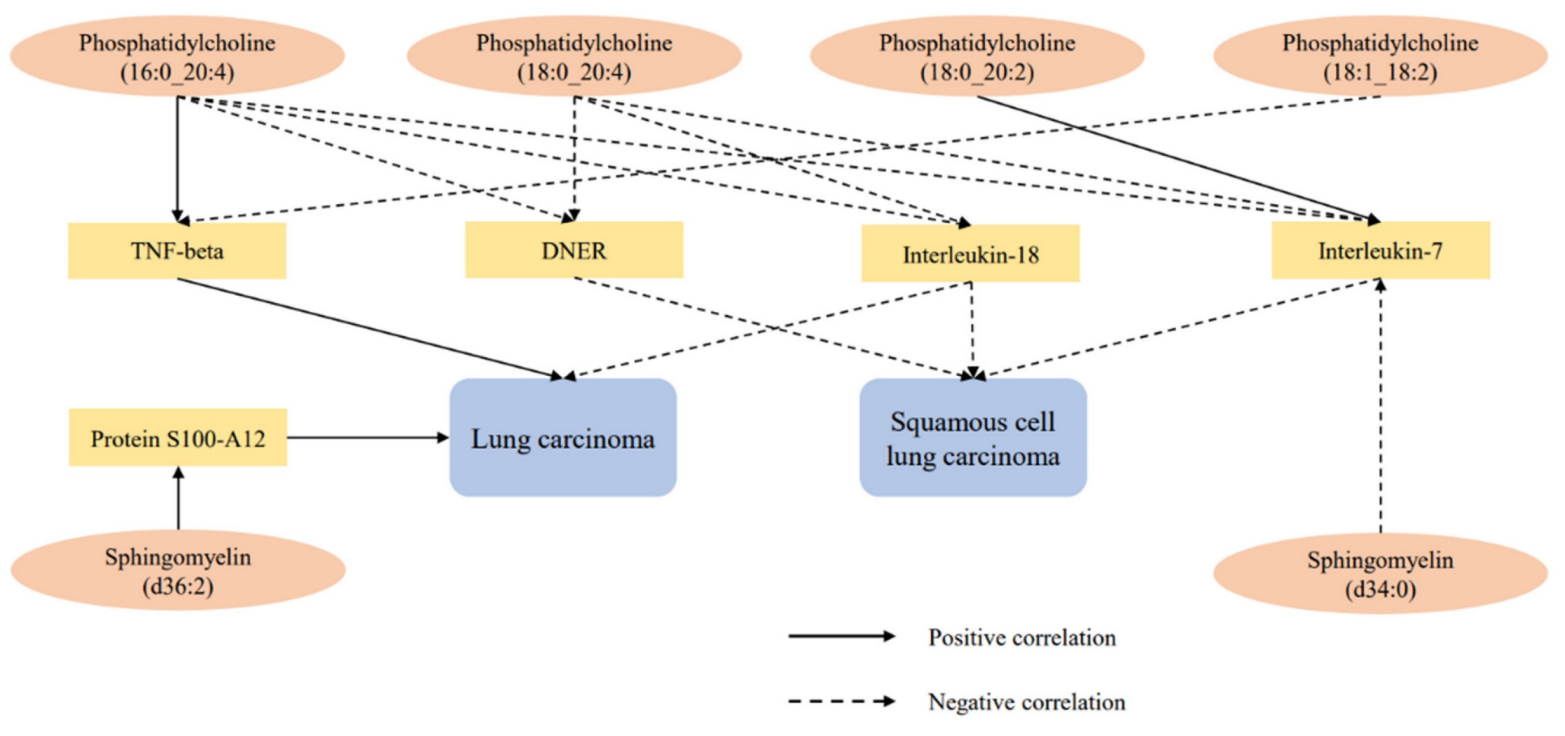
Regulatory network of lung carcinoma and subtypes by plasma lipidome and inflammatory proteins.

After excluding the above-mentioned inflammatory proteins, we finally identified 5 inflammatory proteins that exerted mediating effects (**Supplementary Table S12 and Figure 4**). Specifically, protein S100-A12 levels mediate the effect of SM (d36:2) levels on LC, with a mediation effect accounting for 8.7%; TNF-beta levels mediate the effects of PC (18:1_18:2) and PC (16:0_20:4) levels on LC, with mediation effects of 3.5% and 9.6%, respectively; IL-7 levels mediate the effects of PC (18:0_20:2), PC (16:0_20:4), SM (d34:0), and PC (18:0_20:4) levels on SqCLC, with mediation effects of 9.2%, 11.3%, 7.1%, and 16.4%, respectively; IL-18 levels mediate not only the effect of PC (16:0_20:4) levels on LC (mediation effect of 9.6%) but also the effects of PC (16:0_20:4) and PC (18:0_20:4) levels on SqCLC, with mediation effects of 5.3% and 8.0%, respectively. Additionally, delta and Notch-like epidermal growth factor-related receptor (DNER) levels mediate the effects of PC (16:0_20:4) and PC (18:0_20:4) levels on SqCLC, with mediation effects of 11.7% and 10.2%, respectively (**Supplementary Table S12 and Figure 5**). No evidence was found for other inflammatory proteins mediating the causal effects of plasma lipids on LC and subtypes. **Figure 6** provides an intuitive illustration of the regulatory network of plasma lipidome and inflammatory proteins on LC and subtypes.

## Discussion

This study employed an MR design to elucidate the causal relationship between plasma lipidome and LC and subtypes. Mediation MR analysis revealed that inflammatory proteins may play a significant role in the impact of plasma lipidome on LC. Our findings highlight the complex interplay between plasma lipidome, inflammatory proteins, and LC, underscoring the potential for devising preventive strategies.

The lipid metabolism network in cancer cells is highly complex. On one hand, lipid metabolism in cancer cells is regulated not only by intracellular oncogenic signals but also by the tumor microenvironment, which comprises various components, including lipids. On the other hand, aberrant lipid metabolism can alter oncogenic signals in cancer cells, affecting nearby non-cancerous cells by modifying the secretion of substances, including lipids, from cancer cells 25. Excessive lipid levels are also associated with cancer cell proliferation and metastasis. By altering the composition of lipid membranes, cancer cells can invade adjacent tissues and overcome cell death mechanisms. Cancer cells can acquire energy and protect themselves from oxidative stress damage by modulating lipid breakdown and synthesis metabolism. Additionally, cancer cells can exploit lipid metabolism to regulate the activity of immune cells, thereby evading the host's normal immune clearance functions 26. Key genes/proteins associated with lipid metabolism may serve as potential indicators for predicting the prognosis of various cancers 27.

As the most abundant phospholipid in the body, multiple studies have explored the relationship between PC and LC. One study, based on scRNA-seq and lipidomics, found that five PC subclasses could serve as important detection features for early LC 28. Another metabolomic analysis indicated that levels of saturated PC and monounsaturated PC significantly increase in NSCLC, whereas levels of polyunsaturated PC decrease significantly in NSCLC patients 3. A cross-omics analysis focusing on clinical features and lipidomic profiles among different subtypes of LC patients found that PC (19:0-19:0 and 19:0-21:2) is specifically associated with LADC 29. Our study identified 7 subclasses of PC associated with the risk of LC and subtypes. Specifically, a high PC (16:0_20:2), PC (O-16:1_18:0), PC (17:0_18:1), PC (18:0_20:2), PC (18:1_18:1), PC (18:1_18:2), and PC (O-18:1_20:3) levels were associated with a decreased risk of LC, LADC, and SqCLC. On the contrary, a high PC (16:0_20:4), PC (16:0_20:5), PC (17:0_20:4), PC (O-16:0_20:3), PC (O-16:1_20:3), PC (O-16:1_20:4), PC (O-18:0_14:0), and PC (O-18:2_20:4) levels increased the risk of LC, LADC, SqCLC, and SCLC. It is worth noting that different subclasses of PC exhibited bidirectional effects on the risk of LC or subtypes. This suggests that the spatial structure of lipids, such as carbon atoms, double bonds, or isomerism, may affect the proliferation and differentiation of cancer cells.

PE is the second most abundant phospholipid after PC. PE participates in various cellular functions, including activating oxidative phosphorylation, maintaining mitochondrial morphology, and participating in cell death pathways such as ferroptosis and apoptosis 30, 31. Mechanistic studies have found that excessive production of mitochondrial PE can inhibit cancer cell proliferation 32. Evidence from retrospective studies suggests significant differences in the levels of PE among patients with malignant or benign pulmonary nodules 33. However, cross-omics analysis revealed that specific PE subtype levels are significantly higher in SCLC compared to other LC subtypes or healthy controls 29. This study found that high PE (O-18:1_20:4), PE (18:0_18:2), and PE (O-18:2_18:1) levels are associated with a low LADC or SqCLC risk. Our results further support the role of PE as a protective factor against LC.

DAG, as a lipid second messenger, is involved in transmitting intracellular signals that influence mammalian cell proliferation, survival, and motility. DAG has been widely implicated in the onset, progression, and metastasis of cancers 34. Some molecules that indirectly regulate DAG, such as protein kinase C (PKC) isoform PKCε and phosphatidic acid phosphatase Lipin-1, are associated with the progression and adverse outcomes of LC 35, 36. Our study provided further evidence that elevated DAG (16:1_18:1) and DAG (16:0_18:2) levels are associated with an increased LC and LADC risk.

Our study also demonstrated that elevated SM(d36:2) and SM(d34:0) levels are associated with an increased LC, SqCLC, or SCLC risk. A case-control study conducted in Japan supports our findings 37. This study found that increased SM levels in primary LC tissue samples are associated with an increased risk of LC recurrence. Furthermore, we also found that elevated genetically predicted TAG (48:0) and TAG (58:7) levels are associated with an increased LADC risk, while increased SE (27:1/20:2) levels are associated with a low LC and LADC risk. High PI (18:1_20:4) levels are associated with a lower SCLC risk. However, there is currently no observational evidence linking these three lipids to LC.

Our mediation MR analysis also revealed the involvement of inflammatory proteins in the causal effect of plasma lipidome on LC or subtypes. Among them, IL-7 and IL-18 are both pro-inflammatory cytokines, and several studies have reported the therapeutic potential of IL-7 and IL-18 in LC 38-42. Our MR analysis revealed that high IL-7 and IL-18 levels are associated with a low LC or SqCLC risk. PC (18:0_20:2), PC (16:0_20:4), and PC (16:0_20:4) levels may potentially influence the incidence of SqCLC by modulating the levels of IL-7. PC (16:0_20:4) may also increase the risk of LC and SqCLC by reducing the levels of IL-18. Additionally, protein S100-A12 is involved in the regulation of SM (d36:2) for LC. TNF-beta partially mediates the effects of PC (18:1_18:2) and PC (16:0_20:4) on LC. The causal effects of PC (16:0_20:4) and PC (18:0_20:4) on SqCLC are partially mediated by DNER. These three inflammatory proteins have been reported to be associated with prostate cancer, papillary thyroid carcinoma, ovarian cancer, gastric cancer, and breast cancer 43-47. Our study is the first to identify their involvement in the effects of the lipidome on LC or subtypes. The mechanisms of these inflammatory proteins on LC require further investigation.

This is the first study to systematically explore the causal relationship between plasma lipidome and LC and subtypes using MR design. We also identified 5 inflammatory proteins playing crucial mediating roles in the association between plasma lipidome and LC and subtypes. However, MR methods have limitations that need to be acknowledged. First, despite using SNPs as genetic instruments to mitigate confounding effects, some unmeasured confounders that influence the genetic instruments may still bias the results of MR analysis. In this study, we rigorously employed 4 additional MR methods, the BMA-MR approach, and sensitivity analyses to maximize the reduction of potential biases from unmeasured confounders. Second, to obtain adequate genetic instruments, our study used a threshold of p < 1E-5 for SNP screening. However, this may violate the MR assumption that there is a strong association between genetic instruments and exposure. Therefore, further MR analysis is needed when more appropriate genetic instruments are available. Finally, this MR study was primarily based on European populations. Since the same genetic instruments may have different effects in different ethnic groups, this may limit the generalizability of our results to broader populations.

In conclusion, our findings underscore the intricate network between plasma lipidome and inflammatory proteins in regulating LC. Further research is needed to identify the key lipids and inflammatory proteins that play critical roles in cancer development, along with corresponding functional studies and experimental validations to elucidate the underlying mechanisms involved. Additionally, our study highlights the potential of integrating plasma lipidome into clinical practice. Incorporating plasma lipidome into cancer risk assessment and prevention strategies may pave the way for new approaches in the prevention and management of LC.

## Supplementary Material

Supplementary tables.

## Abbreviations

LC: lung carcinoma
PC: phosphatidylcholines
PE: phosphatidylethanolamines
DAG: diacylglycerols
TAG: triacylglycerols
SM: sphingomyelins
PI: phosphatidylinositols
CE: cholesterol esters
SE: sterol esters
NSCLC: non-small cell lung carcinoma
LADC: lung adenocarcinoma
SqCLC: squamous cell carcinoma
SCLC: small cell lung carcinoma
CRP: C-reactive protein
MIG: monokine induced by gamma interferon
SAA: serum amyloid A
MR: Mendelian Randomization
GWAS: genome-wide association studies
pQTL: protein quantitative trait locus
ILCCO: International Lung Carcinoma Consortium
TRICL: Transdisciplinary Research in Cancer of the Lung
SNPs: single nucleotide polymorphisms
LD: linkage disequilibrium
IVW: inverse variance-weighted
OR: odds ratios
CI: confidence intervals
MR-BMA: MR Bayesian model averaging
DNER: delta and Notch-like epidermal growth factor-related receptor
PKC: protein kinase C
