# Supplementary material for: Causal association of plasma lipidome with lung carcinoma and mediating role of inflammatory proteins: evidence from Mendelian randomization analysis
Source: J Cancer. 2024 Sep 3;15(17):5643–54. doi: 10.7150/jca.99990 (PMC11414616; doi:10.7150/jca.99990)
Supplement: Supplementary file 1 — Supplementary tables. [file jcav15p5643s1.pdf]

Supplementary material

Causal association of plasma lipidome with lung cancer and mediating role of inflammatory proteins: evidence from Mendelian randomization analysis

Supplementary table S1

STROBE checklist of the present study.

	Item No.	Recommendation	Page No.
**Title and abstract**	1	(*a*) Indicate the study's design with a commonly used term in the title or the abstract	1
		(*b*) Provide in the abstract an informative and balanced summary of what was done and what was found	1
**Introduction**			
Background/rationale	2	Explain the scientific background and rationale for the investigation being reported	1-2
Objectives	3	State specific objectives, including any prespecified hypotheses	2
**Methods**			
Study design	4	Present key elements of study design early in the paper	2-3
Setting	5	Describe the setting, locations, and relevant dates, including periods of recruitment, exposure, follow-up, and data collection	3-4
Participants	6	(*a*) *Cohort study*-Give the eligibility criteria, and the sources and methods of selection of participants. Describe methods of follow-up	3-4
		Case-control study-Give the eligibility criteria, and the sources and methods of case ascertainment and control selection. Give the rationale for the choice of cases and controls	
		Cross-sectional study-Give the eligibility criteria, and the sources and methods of selection of participants	
		(*b*) *Cohort study*-For matched studies, give matching criteria and number of exposed and unexposed	NA
		Case-control study-For matched studies, give matching criteria and the number of controls per case	
Variables	7	Clearly define all outcomes, exposures, predictors, potential confounders, and effect modifiers. Give diagnostic criteria, if applicable	4-5
Data sources/ measurement	8	For each variable of interest, give sources of data and details of methods of assessment (measurement). Describe comparability of assessment methods if there is more than one group	4-5, Table S2
Bias	9	Describe any efforts to address potential sources of bias	4-5

	Item No.	Recommendation	Page No.
Study size	10	Explain how the study size was arrived at	3-4
Quantitative variables	11	Explain how quantitative variables were handled in the analyses. If applicable, describe which groupings were chosen and why	4-6
Statistical methods	12	(*a*) Describe all statistical methods, including those used to control for confounding	4-6
		(*b*) Describe any methods used to examine subgroups and interactions	4-6
		(*c*) Explain how missing data were addressed	NA
		(*d*) *Cohort study*-If applicable, explain how loss to follow-up was addressed	NA
		Case-control study-If applicable, explain how matching of cases and controls was addressed	
		Cross-sectional study-If applicable, describe analytical methods taking account of sampling strategy	
		(*e*) Describe any sensitivity analyses	4-5
**Results**			
Participants	13 [a]	(a) Report numbers of individuals at each stage of study-eg numbers potentially eligible, examined for eligibility, confirmed eligible, included in the study, completing follow-up, and analyzed	6-9
		(b) Give reasons for non-participation at each stage	NA
		(c) Consider use of a flow diagram	NA
Descriptive data	14 [a]	(a) Give characteristics of study participants (eg demographic, clinical, social) and information on exposures and potential confounders	3-4
		(b) Indicate number of participants with missing data for each variable of interest	NA
		(c) *Cohort study*-Summarise follow-up time (eg, average and total amount)	NA
Outcome data	15 [a]	Cohort study-Report numbers of outcome events or summary measures over time	NA
		Case-control study-Report numbers in each exposure category, or summary measures of exposure	NA
		Cross-sectional study-Report numbers of outcome events or summary measures	NA
Main results	16	(*a*) Give unadjusted estimates and, if applicable, confounder-adjusted estimates and their precision (eg, 95% confidence interval). Make clear which confounders were adjusted for and why they were included	6-9
		(*b*) Report category boundaries when continuous variables were categorized	NA
		(*c*) If relevant, consider translating estimates of relative risk into absolute risk for a meaningful time period	NA
Other analyses	17	Report other analyses done-eg analyses of subgroups and interactions, and sensitivity analyses	6-9, Figure1-6
**Discussion**			

	Item No.	Recommendation	Page No.
Key results	18	Summarize key results with reference to study objectives	11
Limitations	19	Discuss limitations of the study, taking into account sources of potential bias or imprecision. Discuss both direction and magnitude of any potential bias	13
Interpretation	20	Give a cautious overall interpretation of results considering objectives, limitations, multiplicity of analyses, results from similar studies, and other relevant evidence	11-13
Generalizability	21	Discuss the generalizability (external validity) of the study results	11-13
**Other information**			
Funding	22	Give the source of funding and the role of the funders for the present study and, if applicable, for the original study on which the present article is based	20

STROBE, strengthening the reporting of observational studies in epidemiology; NA, not applicable; ST, supplementary table; F, figure.

[a] Give information separately for cases and controls in case-control studies and, if applicable, for exposed and unexposed groups in cohort and cross-sectional studies.

Supplementary table S2. Overview of GWAS data used in mediation Mendelian randomization.

Phenotype	Sample size	Cases	Controls	Ancestry	Author	PubMed ID
Plasma lipidome	7,266	-	-	European	Ottensmann L et al.	37907536
Circulating inflammatory proteins	14,824	-	-	European	Zhao JH et al.	37563310
Lung carcinoma	85,716	29,266	56,450	European	McKay JD et al.	28604730
Lung adenocarcinoma	66,756	11,273	55,483	European	McKay JD et al.	28604730
Squamous cell lung carcinoma	63,053	7,426	55,627	European	McKay JD et al.	28604730
Small cell lung carcinoma	24,108	2,664	21,444	European	McKay JD et al.	28604730

Supplementary table S3. Summary information for SNPs that were used as genetic instruments for Mendelian randomization analyses of plasma lipidome.

Phenotype	SNP	chromosome	base_pair_location	effect_allele	other_allele	BETA	SE	EAF	N	p-value	R2	F
Sterol ester (27:1/20:2) levels	rs675505	2	25435229	C	G	-	0.03	0.93	584	1.33E-	0.004	23.42

	7					0.180	7	5	8	06	0	
Sterol ester (27:1/20:2) levels	rs7933	2	43225044	G	A	0.106	0.020	0.329	5848	9.14E-08	0.0049	28.61
Sterol ester (27:1/20:2) levels	rs4245791	2	43847292	T	C	-0.256	0.022	0.773	5848	9.13E-32	0.0232	139.13
Sterol ester (27:1/20:2) levels	rs6709904	2	43853185	G	A	-0.211	0.032	0.100	5848	3.77E-11	0.0074	43.88
Sterol ester (27:1/20:2) levels	rs79849855	2	60821224	C	T	-0.311	0.067	0.020	5848	4.07E-06	0.0036	21.26
Sterol ester (27:1/20:2) levels	rs77590808	2	165115925	A	G	-0.388	0.087	0.012	5848	9.32E-06	0.0034	19.67
Sterol ester (27:1/20:2) levels	rs36194627	2	225135785	A	G	0.293	0.064	0.023	5848	4.70E-06	0.0036	20.99
Sterol ester (27:1/20:2) levels	rs75823353	3	7487632	G	A	0.169	0.036	0.073	5848	2.39E-06	0.0038	22.29
Sterol ester (27:1/20:2) levels	rs182695896	4	73947510	C	A	0.286	0.059	0.025	5848	1.52E-06	0.0039	23.16
Sterol ester (27:1/20:2) levels	rs13121156	4	96997008	C	T	-0.087	0.020	0.320	5848	9.25E-06	0.0034	19.69
Sterol ester (27:1/20:2) levels	rs74726976	5	90954717	T	C	0.227	0.051	0.034	5848	9.64E-06	0.0033	19.61
Sterol ester (27:1/20:2) levels	rs114838148	5	180649403	T	C	0.247	0.055	0.031	5848	7.99E-06	0.0034	19.97
Sterol ester (27:1/20:2) levels	rs9504426	6	5531009	G	C	-0.092	0.019	0.378	5848	1.16E-06	0.0040	23.68

Sterol ester (27:1/20:2) levels	rs12527805	6	138623575	C	T	0.131	0.029	0.119	5848	6.29E-06	0.0035	20.43
Sterol ester (27:1/20:2) levels	rs1404999	7	39081865	T	C	-0.102	0.020	0.314	5848	3.99E-07	0.0044	25.75
Sterol ester (27:1/20:2) levels	rs55837134	7	44532058	A	G	0.099	0.022	0.239	5848	4.95E-06	0.0036	20.89
Sterol ester (27:1/20:2) levels	rs700320	7	147774030	T	C	-0.096	0.021	0.269	5848	5.31E-06	0.0035	20.75
Sterol ester (27:1/20:2) levels	rs56276984	8	23548281	T	C	-0.168	0.036	0.069	5848	3.57E-06	0.0037	21.51
Sterol ester (27:1/20:2) levels	rs7853989	9	133256205	C	G	-0.133	0.026	0.156	5848	2.41E-07	0.0046	26.73
Sterol ester (27:1/20:2) levels	rs118137821	10	7348594	T	C	-0.269	0.059	0.026	5848	4.91E-06	0.0036	20.90
Sterol ester (27:1/20:2) levels	rs10826704	10	29809803	T	C	-0.173	0.038	0.067	5848	5.50E-06	0.0035	20.68
Sterol ester (27:1/20:2) levels	rs1903973	10	53105888	A	G	0.086	0.018	0.556	5848	2.62E-06	0.0038	22.11
Sterol ester (27:1/20:2) levels	rs2244334	10	69935534	C	T	-0.191	0.043	0.055	5848	7.39E-06	0.0034	20.12
Sterol ester (27:1/20:2) levels	rs6490297	12	120617356	C	T	-0.094	0.020	0.313	5848	2.04E-06	0.0038	22.59
Sterol ester (27:1/20:2) levels	rs4778971	15	79180180	G	A	-0.09	0.020	0.609	5848	4.61E-06	0.0036	21.02

						0						
Sterol ester (27:1/20:2) levels	rs7202845	16	26717568	C	A	0.099	0.020	0.670	5848	6.41E-07	0.0042	24.83
Sterol ester (27:1/20:2) levels	rs429358	19	44908684	C	T	0.162	0.024	0.189	5848	9.20E-12	0.0079	46.66
Cholesterol levels	rs11591147	1	55039974	T	G	-0.214	0.047	0.033	7166	5.51E-06	0.0029	20.67
Cholesterol levels	rs12562141	1	181952433	C	T	-0.139	0.030	0.083	7166	4.52E-06	0.0029	21.06
Cholesterol levels	rs180958041	2	170149397	T	C	-0.365	0.077	0.012	7166	2.22E-06	0.0031	22.43
Cholesterol levels	rs34462361	2	215511513	G	A	0.085	0.019	0.256	7166	8.05E-06	0.0028	19.95
Cholesterol levels	rs669552	3	172335874	G	A	-0.108	0.023	0.156	7166	3.40E-06	0.0030	21.60
Cholesterol levels	rs143214193	3	194964435	A	G	-0.495	0.109	0.008	7166	5.35E-06	0.0029	20.73
Cholesterol levels	rs11730766	4	72366511	T	C	0.253	0.052	0.029	7166	9.67E-07	0.0033	24.03
Cholesterol levels	rs77645768	4	72803111	A	G	0.242	0.054	0.026	7166	7.16E-06	0.0028	20.17
Cholesterol levels	rs182695896	4	73947510	C	A	0.268	0.053	0.025	7166	5.02E-07	0.0035	25.29
Cholesterol levels	rs75059546	5	126190702	A	C	-0.278	0.061	0.020	7166	5.45E-06	0.0029	20.70
Cholesterol levels	rs223747	7	50678097	T	C	-	0.02	0.19	716	2.52E-	0.003	22.18

	8					0.099	1	8	6	06	1	
Cholesterol levels	rs11781607	8	11644899	A	G	-0.081	0.018	0.322	7166	7.89E-06	0.0028	19.99
Cholesterol levels	rs112411704	9	2958367	G	A	0.303	0.066	0.018	7166	4.51E-06	0.0029	21.06
Cholesterol levels	rs10812660	9	27742537	A	T	0.086	0.018	0.375	7166	1.57E-06	0.0032	23.09
Cholesterol levels	rs78721266	9	96213656	G	A	0.191	0.041	0.044	7166	3.90E-06	0.0030	21.34
Cholesterol levels	rs12552499	9	121135217	G	A	-0.189	0.040	0.047	7166	2.50E-06	0.0031	22.19
Cholesterol levels	rs2879630	10	51918584	A	G	-0.101	0.021	0.189	7166	1.80E-06	0.0032	22.83
Cholesterol levels	rs7909144	10	52240944	G	A	0.076	0.017	0.593	7166	7.66E-06	0.0028	20.04
Cholesterol levels	rs12366015	11	117120135	G	A	-0.082	0.019	0.717	7166	9.91E-06	0.0027	19.55
Cholesterol levels	rs1271671	12	31810895	A	G	0.235	0.051	0.028	7166	4.70E-06	0.0029	20.99
Cholesterol levels	rs1259751	12	31822701	T	C	0.238	0.051	0.028	7166	4.02E-06	0.0030	21.29
Cholesterol levels	rs75611814	13	23150340	C	T	0.124	0.027	0.103	7166	5.82E-06	0.0029	20.57
Cholesterol levels	rs11858279	15	58420774	C	T	0.088	0.018	0.303	7166	1.43E-06	0.0032	23.27
Cholesterol levels	rs11857386	15	58428969	C	G	-0.07	0.017	0.471	7166	4.57E-06	0.0029	21.04

						9						
Cholesterol levels	rs16963800	16	14576910	C	T	0.110	0.023	0.160	7166	1.36E-06	0.0033	23.37
Cholesterol levels	rs223841	16	57423359	G	A	-0.098	0.022	0.172	7166	9.81E-06	0.0027	19.57
Cholesterol levels	rs141401656	18	35844265	C	T	-0.303	0.068	0.015	7166	9.94E-06	0.0027	19.54
Cholesterol levels	rs17702736	18	39850559	C	T	-0.077	0.017	0.389	7166	8.24E-06	0.0028	19.90
Cholesterol levels	rs118068660	19	11079868	T	C	-0.161	0.028	0.095	7166	1.16E-08	0.0045	32.62
Cholesterol levels	rs73005445	19	15830645	G	A	-0.090	0.019	0.253	7166	2.94E-06	0.0030	21.88
Cholesterol levels	rs429358	19	44908684	C	T	0.106	0.022	0.189	7166	9.26E-07	0.0034	24.11
Cholesterol levels	rs114243848	19	49823108	C	A	-0.228	0.042	0.042	7166	7.17E-08	0.0040	29.07
Cholesterol levels	rs57405165	20	40665923	T	C	-0.107	0.023	0.155	7166	3.52E-06	0.0030	21.54
Cholesterol levels	rs1800961	20	44413724	T	C	-0.180	0.037	0.052	7166	1.57E-06	0.0032	23.09
Cholesterol levels	rs76093749	22	50075106	T	C	0.395	0.087	0.010	7166	5.10E-06	0.0029	20.82
Diacylglycerol (16:0_18:2)	rs765878	1	186849811	T	G	0.17	0.04	0.05	607	9.36E-	0.003	19.66

levels	48					8	0	8	5	06	2	
Diacylglycerol (16:0_18:2) levels	rs6687291	1	230586281	A	G	0.098	0.020	0.320	6075	7.54E-07	0.0040	24.51
Diacylglycerol (16:0_18:2) levels	rs2004776	1	230712956	T	C	-0.108	0.023	0.196	6075	2.83E-06	0.0036	21.96
Diacylglycerol (16:0_18:2) levels	rs1260326	2	27508073	C	T	-0.102	0.019	0.651	6075	6.21E-08	0.0048	29.36
Diacylglycerol (16:0_18:2) levels	rs7569544	2	42290376	A	C	-0.091	0.021	0.265	6075	9.64E-06	0.0032	19.61
Diacylglycerol (16:0_18:2) levels	rs78504564	2	42953102	T	C	-0.630	0.134	0.005	6075	2.49E-06	0.0036	22.21
Diacylglycerol (16:0_18:2) levels	rs140314539	2	220215369	A	G	0.308	0.064	0.021	6075	1.38E-06	0.0038	23.35
Diacylglycerol (16:0_18:2) levels	rs149992851	3	55269076	G	A	0.293	0.065	0.020	6075	7.72E-06	0.0033	20.03
Diacylglycerol (16:0_18:2) levels	rs6810359	3	188792426	T	G	-0.192	0.041	0.053	6075	3.61E-06	0.0035	21.49
Diacylglycerol (16:0_18:2) levels	rs34606692	5	149755706	T	C	-0.114	0.023	0.196	6075	7.38E-07	0.0040	24.56
Diacylglycerol (16:0_18:2) levels	rs758398	6	28352897	T	C	-0.116	0.023	0.183	6075	7.42E-07	0.0040	24.54
Diacylglycerol (16:0_18:2) levels	rs11756450	6	64409802	A	C	0.094	0.019	0.324	6075	1.61E-06	0.0038	23.05
Diacylglycerol (16:0_18:2) levels	rs17145750	7	73612048	T	C	-0.13	0.026	0.141	6075	3.14E-07	0.0043	26.21

						4						
Diacylglycerol (16:0_18:2) levels	rs111934503	8	130543708	C	T	0.669	0.140	0.005	6075	1.66E-06	0.0038	22.99
Diacylglycerol (16:0_18:2) levels	rs34792804	9	8556190	G	T	-0.173	0.039	0.060	6075	8.70E-06	0.0033	19.80
Diacylglycerol (16:0_18:2) levels	rs148163011	9	71279650	G	C	-0.281	0.060	0.024	6075	2.83E-06	0.0036	21.96
Diacylglycerol (16:0_18:2) levels	rs112122274	9	136257994	G	A	-0.253	0.053	0.036	6075	1.68E-06	0.0038	22.96
Diacylglycerol (16:0_18:2) levels	rs4262724	11	23915220	A	C	0.092	0.020	0.717	6075	6.46E-06	0.0033	20.37
Diacylglycerol (16:0_18:2) levels	rs964184	11	116778201	C	G	-0.225	0.025	0.849	6075	1.64E-19	0.0133	82.15
Diacylglycerol (16:0_18:2) levels	rs57747810	12	19924761	A	C	-0.404	0.084	0.012	6075	1.70E-06	0.0038	22.94
Diacylglycerol (16:0_18:2) levels	rs11182480	12	44386579	C	T	0.341	0.071	0.018	6075	1.78E-06	0.0037	22.86
Diacylglycerol (16:0_18:2) levels	rs7165355	15	38675336	C	G	-0.107	0.023	0.192	6075	3.92E-06	0.0035	21.34
Diacylglycerol (16:0_18:2) levels	rs1032956	15	97687850	C	T	0.083	0.019	0.451	6075	7.88E-06	0.0033	19.99
Diacylglycerol (16:0_18:2) levels	rs2868424	16	11584433	A	T	0.111	0.025	0.165	6075	8.47E-06	0.0033	19.86
Diacylglycerol (16:0_18:2) levels	rs35986054	17	1623049	C	T	0.107	0.023	0.196	6075	4.30E-06	0.0035	21.16
Diacylglycerol (16:0_18:2)	rs480690	19	3057222	C	T	0.12	0.02	0.15	607	4.18E-	0.003	21.21

levels	1					1	6	1	5	06	5	
Diacylglycerol (16:0_18:2) levels	rs7254036	19	41202334	T	C	0.082	0.018	0.532	6075	7.01E-06	0.0033	20.22
Diacylglycerol (16:0_18:2) levels	rs144838649	21	44845081	T	C	-0.580	0.125	0.005	6075	3.52E-06	0.0035	21.54
Diacylglycerol (16:1_18:1) levels	rs143808674	1	81671654	G	A	-0.186	0.042	0.048	6215	8.76E-06	0.0032	19.79
Diacylglycerol (16:1_18:1) levels	rs1260326	2	27508073	C	T	-0.112	0.019	0.651	6215	2.15E-09	0.0057	35.92
Diacylglycerol (16:1_18:1) levels	rs10022344	4	124943164	T	C	0.106	0.024	0.181	6215	8.41E-06	0.0032	19.87
Diacylglycerol (16:1_18:1) levels	rs2524075	6	31276082	T	C	-0.107	0.022	0.208	6215	9.22E-07	0.0039	24.12
Diacylglycerol (16:1_18:1) levels	rs2182380	6	43900875	G	A	0.261	0.054	0.031	6215	1.65E-06	0.0037	23.00
Diacylglycerol (16:1_18:1) levels	rs77134026	7	36495127	A	G	0.258	0.055	0.031	6215	3.20E-06	0.0035	21.72
Diacylglycerol (16:1_18:1) levels	rs11775636	8	142752432	A	G	-0.113	0.024	0.158	6215	3.05E-06	0.0035	21.81
Diacylglycerol (16:1_18:1) levels	rs3829088	9	23713682	C	T	0.102	0.022	0.203	6215	5.65E-06	0.0033	20.63
Diacylglycerol (16:1_18:1) levels	rs112122274	9	136257994	G	A	-0.260	0.053	0.036	6215	7.58E-07	0.0039	24.50
Diacylglycerol (16:1_18:1) levels	rs964184	11	116778201	C	G	-0.164	0.025	0.849	6215	3.17E-11	0.0071	44.21

Diacylglycerol (16:1_18:1) levels	rs11182480	12	44386579	C	T	0.345	0.070	0.018	6215	7.16E-07	0.0039	24.61
Diacylglycerol (16:1_18:1) levels	rs7955732	12	72331215	T	G	-0.089	0.018	0.423	6215	1.24E-06	0.0038	23.54
Diacylglycerol (16:1_18:1) levels	rs111750990	12	96298692	C	A	-0.214	0.046	0.041	6215	2.65E-06	0.0035	22.08
Diacylglycerol (16:1_18:1) levels	rs12050879	15	39983732	A	G	-0.278	0.062	0.021	6215	7.99E-06	0.0032	19.97
Diacylglycerol (16:1_18:1) levels	rs4419068	16	71309806	A	G	-0.093	0.021	0.237	6215	9.89E-06	0.0031	19.56
Diacylglycerol (16:1_18:1) levels	rs908150	17	66540315	C	T	0.081	0.018	0.428	6215	9.56E-06	0.0031	19.62
Diacylglycerol (16:1_18:1) levels	rs10502551	18	30887893	G	C	-0.213	0.042	0.051	6215	3.21E-07	0.0042	26.17
Diacylglycerol (16:1_18:1) levels	rs147782497	18	78352143	A	C	0.267	0.060	0.024	6215	9.11E-06	0.0032	19.71
Diacylglycerol (16:1_18:1) levels	rs140283718	19	30685856	A	T	-0.266	0.057	0.026	6215	3.29E-06	0.0035	21.67
Diacylglycerol (16:1_18:1) levels	rs78263224	19	52961172	C	T	0.206	0.046	0.043	6215	7.61E-06	0.0032	20.06
Diacylglycerol (16:1_18:1) levels	rs117771237	22	40189878	A	C	0.384	0.085	0.012	6215	7.02E-06	0.0032	20.22
Diacylglycerol (18:1_18:2) levels	rs72694990	1	150251390	A	G	-0.121	0.026	0.129	6613	4.00E-06	0.0032	21.29
Diacylglycerol (18:1_18:2)	rs346217	1	220806736	A	C	0.11	0.02	0.18	661	6.40E-	0.003	24.83

levels	09					2	2	5	3	07	7	
Diacylglycerol (18:1_18:2) levels	rs2352723	1	230156121	C	T	-0.112	0.020	0.769	6613	3.76E-08	0.0046	30.33
Diacylglycerol (18:1_18:2) levels	rs1042034	2	21002409	T	C	0.093	0.019	0.728	6613	1.65E-06	0.0035	23.00
Diacylglycerol (18:1_18:2) levels	rs1260326	2	27508073	C	T	-0.129	0.018	0.651	6613	9.98E-13	0.0077	51.03
Diacylglycerol (18:1_18:2) levels	rs11684166	2	111060064	A	G	0.106	0.023	0.177	6613	3.49E-06	0.0032	21.55
Diacylglycerol (18:1_18:2) levels	rs9973939	2	212078809	G	A	-0.082	0.018	0.660	6613	8.26E-06	0.0030	19.90
Diacylglycerol (18:1_18:2) levels	rs116011373	3	87298641	A	G	-0.182	0.041	0.049	6613	7.38E-06	0.0030	20.12
Diacylglycerol (18:1_18:2) levels	rs57500102	3	171536246	A	G	-0.499	0.111	0.007	6613	7.12E-06	0.0030	20.19
Diacylglycerol (18:1_18:2) levels	rs114444157	3	176180291	C	T	0.149	0.033	0.078	6613	7.23E-06	0.0030	20.16
Diacylglycerol (18:1_18:2) levels	rs62291573	4	4999649	C	T	0.100	0.022	0.214	6613	4.75E-06	0.0032	20.96
Diacylglycerol (18:1_18:2) levels	rs17007519	4	141672748	T	C	-0.301	0.060	0.022	6613	6.24E-07	0.0037	24.87
Diacylglycerol (18:1_18:2) levels	rs4691907	4	163302506	T	G	0.078	0.017	0.532	6613	7.60E-06	0.0030	20.06
Diacylglycerol (18:1_18:2) levels	rs7745829	6	14146172	G	A	-0.112	0.025	0.147	6613	5.66E-06	0.0031	20.63

Diacylglycerol (18:1_18:2) levels	rs1233660	6	28292472	A	T	-0.098	0.021	0.221	6613	3.00E-06	0.0033	21.85
Diacylglycerol (18:1_18:2) levels	rs75008187	6	64441454	A	G	0.129	0.026	0.128	6613	9.80E-07	0.0036	24.00
Diacylglycerol (18:1_18:2) levels	rs141116593	6	154880792	G	C	0.155	0.033	0.074	6613	2.82E-06	0.0033	21.96
Diacylglycerol (18:1_18:2) levels	rs7782961	7	6295952	T	A	0.092	0.021	0.232	6613	9.94E-06	0.0029	19.55
Diacylglycerol (18:1_18:2) levels	rs79624003	7	73598455	G	A	-0.172	0.027	0.122	6613	8.98E-11	0.0063	42.15
Diacylglycerol (18:1_18:2) levels	rs268	8	19956018	G	A	0.336	0.058	0.023	6613	8.26E-09	0.0050	33.29
Diacylglycerol (18:1_18:2) levels	rs75596627	8	43650502	A	G	0.128	0.028	0.114	6613	4.62E-06	0.0032	21.01
Diacylglycerol (18:1_18:2) levels	rs117416257	10	5793091	T	C	-0.604	0.131	0.005	6613	3.83E-06	0.0032	21.38
Diacylglycerol (18:1_18:2) levels	rs75082203	10	88606986	A	G	0.349	0.078	0.013	6613	8.40E-06	0.0030	19.87
Diacylglycerol (18:1_18:2) levels	rs72836628	10	112142696	T	C	-0.090	0.019	0.297	6613	2.11E-06	0.0034	22.52
Diacylglycerol (18:1_18:2) levels	rs138578034	11	17897155	T	G	-0.689	0.154	0.003	6613	7.80E-06	0.0030	20.01
Diacylglycerol (18:1_18:2) levels	rs181215046	11	32530936	G	T	-0.477	0.105	0.007	6613	5.32E-06	0.0031	20.75
Diacylglycerol (18:1_18:2) levels	rs142868058	11	62717059	T	C	-0.55	0.125	0.005	6613	8.45E-06	0.0030	19.86

						9						
Diacylglycerol (18:1_18:2) levels	rs12365864	11	116618319	G	A	0.124	0.024	0.166	6613	1.58E-07	0.0041	27.54
Diacylglycerol (18:1_18:2) levels	rs964184	11	116778201	C	G	-0.291	0.024	0.849	6613	3.26E-34	0.0222	150.38
Diacylglycerol (18:1_18:2) levels	rs10773708	12	129796667	A	G	-0.087	0.019	0.683	6613	3.71E-06	0.0032	21.44
Diacylglycerol (18:1_18:2) levels	rs55788915	16	23267056	A	C	-0.251	0.053	0.031	6613	1.92E-06	0.0034	22.70
Diacylglycerol (18:1_18:2) levels	rs7239207	18	62009100	C	T	-0.086	0.019	0.694	6613	4.99E-06	0.0031	20.87
Diacylglycerol (18:1_18:2) levels	rs4608457	19	20106360	T	C	-0.200	0.042	0.045	6613	1.81E-06	0.0034	22.82
Diacylglycerol (18:1_18:2) levels	rs11670344	19	33144357	C	A	0.087	0.019	0.278	6613	5.24E-06	0.0031	20.77
Diacylglycerol (18:1_18:2) levels	rs157582	19	44892962	T	C	0.118	0.021	0.223	6613	1.66E-08	0.0048	31.93
Diacylglycerol (18:1_18:2) levels	rs11668758	19	44971423	T	C	0.096	0.019	0.307	6613	4.54E-07	0.0038	25.49
Phosphatidylcholine (20:4_0:0) levels	rs76469829	1	29962031	T	C	-0.216	0.048	0.037	6235	5.69E-06	0.0033	20.62
Phosphatidylcholine (20:4_0:0) levels	rs76513766	1	172130754	A	G	0.378	0.084	0.012	6235	7.01E-06	0.0032	20.22
Phosphatidylcholine (20:4_0:0) levels	rs9967725	2	56382464	T	A	-0.101	0.019	0.350	6235	6.79E-08	0.0047	29.18

Phosphatidylcholine (20:4_0:0) levels	rs4708901	6	161271441	A	G	-0.099	0.022	0.215	6235	5.37E-06	0.0033	20.73
Phosphatidylcholine (20:4_0:0) levels	rs191895023	7	2289982	A	C	0.805	0.177	0.002	6235	5.63E-06	0.0033	20.64
Phosphatidylcholine (20:4_0:0) levels	rs142999489	7	95974040	G	C	-0.194	0.040	0.056	6235	1.09E-06	0.0038	23.80
Phosphatidylcholine (20:4_0:0) levels	rs62511275	8	116151644	G	C	0.220	0.046	0.042	6235	2.08E-06	0.0036	22.56
Phosphatidylcholine (20:4_0:0) levels	rs10733608	9	114386150	T	G	0.083	0.018	0.485	6235	3.34E-06	0.0035	21.65
Phosphatidylcholine (20:4_0:0) levels	rs10884272	10	106220847	A	G	-0.083	0.018	0.365	6235	6.46E-06	0.0033	20.37
Phosphatidylcholine (20:4_0:0) levels	rs3741252	11	61744026	T	C	0.166	0.026	0.134	6235	3.34E-10	0.0063	39.58
Phosphatidylcholine (20:4_0:0) levels	rs12226389	11	62056158	C	T	-0.176	0.024	0.173	6235	2.15E-13	0.0086	54.07
Phosphatidylcholine (20:4_0:0) levels	rs7936002	11	62451557	G	A	0.134	0.021	0.257	6235	1.65E-10	0.0065	40.97
Phosphatidylcholine (20:4_0:0) levels	rs600626	11	75744264	G	A	-0.121	0.021	0.232	6235	1.23E-08	0.0052	32.51
Phosphatidylcholine (20:4_0:0) levels	rs529446	12	5112153	C	T	0.097	0.022	0.784	6235	7.85E-06	0.0032	20.00
Phosphatidylcholine (20:4_0:0) levels	rs79995028	14	41287117	T	G	-0.653	0.144	0.004	6235	5.86E-06	0.0033	20.56
Phosphatidylcholine (20:4_0:0) levels	rs7179330	15	23837617	T	A	0.313	0.064	0.980	6235	1.15E-06	0.0038	23.70

Phosphatidylcholine (20:4_0:0) levels	rs12905466	15	71480547	T	C	-0.102	0.022	0.204	6235	5.01E-06	0.0033	20.86
Phosphatidylcholine (20:4_0:0) levels	rs6564238	16	75219782	G	C	0.086	0.019	0.329	6235	7.56E-06	0.0032	20.07
Phosphatidylcholine (20:4_0:0) levels	rs150158785	17	49470511	A	G	-0.148	0.033	0.080	6235	7.51E-06	0.0032	20.09
Phosphatidylcholine (20:4_0:0) levels	rs1363011	18	64269741	A	G	0.118	0.027	0.130	6235	1.00E-05	0.0031	19.54
Phosphatidylcholine (20:4_0:0) levels	rs11669519	19	614967	C	T	0.091	0.020	0.341	6235	6.45E-06	0.0033	20.38
Phosphatidylcholine (20:4_0:0) levels	rs182611493	19	19347579	G	A	-0.208	0.040	0.054	6235	2.78E-07	0.0042	26.44
Phosphatidylcholine (20:4_0:0) levels	rs12303	20	64047793	A	G	-0.104	0.023	0.196	6235	4.56E-06	0.0034	21.04
Phosphatidylcholine (20:4_0:0) levels	rs139516883	22	23818551	C	T	-0.353	0.071	0.016	6235	7.61E-07	0.0039	24.50
Phosphatidylcholine (16:0_16:1) levels	rs1260326	2	27508073	C	T	-0.108	0.017	0.651	7172	6.12E-10	0.0053	38.37
Phosphatidylcholine (16:0_16:1) levels	rs80227620	2	167832629	T	C	0.119	0.024	0.144	7172	1.19E-06	0.0033	23.62
Phosphatidylcholine (16:0_16:1) levels	rs28524821	4	17068686	C	A	0.109	0.024	0.146	7172	3.98E-06	0.0030	21.30
Phosphatidylcholine (16:0_16:1) levels	rs2597486	4	162453799	G	A	0.076	0.017	0.554	7172	8.12E-06	0.0028	19.93
Phosphatidylcholine (16:0_16:1) levels	rs116681793	4	180227646	A	G	-0.18	0.041	0.045	7172	7.01E-06	0.0028	20.21

						5						
Phosphatidylcholine (16:0_16:1) levels	rs4629629	5	86296114	T	C	0.088	0.020	0.765	7172	7.25E-06	0.0028	20.15
Phosphatidylcholine (16:0_16:1) levels	rs113366516	6	138148100	T	G	0.308	0.066	0.017	7172	3.14E-06	0.0030	21.76
Phosphatidylcholine (16:0_16:1) levels	rs117726731	8	303655	T	C	0.186	0.042	0.042	7172	9.99E-06	0.0027	19.53
Phosphatidylcholine (16:0_16:1) levels	rs1854434	9	32796762	G	A	-0.110	0.023	0.154	7172	2.15E-06	0.0031	22.49
Phosphatidylcholine (16:0_16:1) levels	rs10760286	9	123419309	C	T	0.079	0.017	0.510	7172	3.32E-06	0.0030	21.65
Phosphatidylcholine (16:0_16:1) levels	rs35235479	10	92767403	G	A	-0.096	0.020	0.214	7172	2.24E-06	0.0031	22.41
Phosphatidylcholine (16:0_16:1) levels	rs62015468	16	6538604	C	G	0.194	0.043	0.042	7172	6.47E-06	0.0028	20.37
Phosphatidylcholine (16:0_16:1) levels	rs11647818	16	11833509	T	C	0.079	0.017	0.575	7172	3.68E-06	0.0030	21.45
Phosphatidylcholine (16:0_16:1) levels	rs77007318	16	59856268	C	G	-0.241	0.052	0.029	7172	3.88E-06	0.0030	21.35
Phosphatidylcholine (16:0_16:1) levels	rs34832413	16	71310208	G	A	-0.119	0.026	0.112	7172	6.08E-06	0.0028	20.49
Phosphatidylcholine (16:0_16:1) levels	rs117571757	17	16458446	A	G	-0.334	0.073	0.013	7172	4.44E-06	0.0029	21.09
Phosphatidylcholine (16:0_16:1) levels	rs62098276	18	63512537	C	T	-0.142	0.031	0.080	7172	3.80E-06	0.0030	21.39
Phosphatidylcholine (16:0_16:1)	rs129606	18	78257046	C	T	0.12	0.02	0.10	717	7.73E-	0.002	20.03

levels	35					4	8	5	2	06	8	
Phosphatidylcholine (16:0_20:2) levels	rs1168041	1	62494579	C	T	0.093	0.019	0.733	7135	9.29E-07	0.0034	24.10
Phosphatidylcholine (16:0_20:2) levels	rs1260326	2	27508073	C	T	-0.097	0.018	0.651	7135	3.00E-08	0.0043	30.77
Phosphatidylcholine (16:0_20:2) levels	rs17268633	2	56194921	G	A	-0.285	0.060	0.020	7135	2.07E-06	0.0032	22.56
Phosphatidylcholine (16:0_20:2) levels	rs78230994	4	26558575	A	G	0.595	0.123	0.006	7135	1.25E-06	0.0033	23.53
Phosphatidylcholine (16:0_20:2) levels	rs142392117	4	176627941	C	T	-0.277	0.057	0.022	7135	1.40E-06	0.0033	23.32
Phosphatidylcholine (16:0_20:2) levels	rs3132935	6	32203298	G	A	-0.097	0.022	0.177	7135	7.80E-06	0.0028	20.01
Phosphatidylcholine (16:0_20:2) levels	rs34516410	6	99968458	T	G	-0.114	0.025	0.133	7135	4.16E-06	0.0030	21.21
Phosphatidylcholine (16:0_20:2) levels	rs12700446	7	23532125	T	C	0.309	0.067	0.016	7135	4.73E-06	0.0029	20.97
Phosphatidylcholine (16:0_20:2) levels	rs2236514	9	136677616	G	C	-0.091	0.018	0.647	7135	3.85E-07	0.0036	25.81
Phosphatidylcholine (16:0_20:2) levels	rs148069564	10	51792352	A	T	-0.777	0.167	0.003	7135	3.28E-06	0.0030	21.67
Phosphatidylcholine (16:0_20:2) levels	rs72800299	10	65395218	A	T	-0.085	0.019	0.274	7135	4.69E-06	0.0029	20.98
Phosphatidylcholine (16:0_20:2)	rs127617	10	90294727	C	T	-	0.02	0.15	713	9.93E-	0.002	19.55

levels	49					0.105	4	0	5	06	7	
Phosphatidylcholine (16:0_20:2) levels	rs174592	11	61851136	G	A	0.262	0.017	0.418	7135	4.96E-54	0.0330	243.53
Phosphatidylcholine (16:0_20:2) levels	rs2317091	11	63193288	A	T	0.218	0.043	0.040	7135	4.11E-07	0.0036	25.68
Phosphatidylcholine (16:0_20:2) levels	rs1815763	11	67075266	A	G	-0.366	0.080	0.011	7135	4.39E-06	0.0030	21.11
Phosphatidylcholine (16:0_20:2) levels	rs10160784	11	75745010	C	T	-0.110	0.020	0.232	7135	3.61E-08	0.0042	30.41
Phosphatidylcholine (16:0_20:2) levels	rs79718024	11	107569619	G	A	-0.113	0.024	0.155	7135	1.76E-06	0.0032	22.87
Phosphatidylcholine (16:0_20:2) levels	rs17128436	11	124146158	C	G	0.309	0.069	0.016	7135	7.29E-06	0.0028	20.14
Phosphatidylcholine (16:0_20:2) levels	rs17126640	12	52311207	A	G	-0.098	0.021	0.203	7135	2.70E-06	0.0031	22.05
Phosphatidylcholine (16:0_20:2) levels	rs1169289	12	120978819	G	C	0.075	0.017	0.470	7135	7.32E-06	0.0028	20.13
Phosphatidylcholine (16:0_20:2) levels	rs113130033	14	89719965	G	A	-0.197	0.041	0.043	7135	1.73E-06	0.0032	22.90
Phosphatidylcholine (16:0_20:2) levels	rs1532085	15	58391167	G	A	-0.094	0.017	0.573	7135	2.69E-08	0.0043	30.98
Phosphatidylcholine (16:0_20:2) levels	rs1800588	15	58431476	T	C	0.107	0.019	0.258	7135	3.85E-08	0.0042	30.28
Phosphatidylcholine (16:0_20:2) levels	rs1640963	16	6506441	C	G	-0.21	0.048	0.032	7135	9.76E-06	0.0027	19.58

						1						
Phosphatidylcholine (16:0_20:2) levels	rs113550786	16	13890509	G	A	0.393	0.083	0.012	7135	2.06E-06	0.0032	22.57
Phosphatidylcholine (16:0_20:2) levels	rs76711488	18	8971803	A	G	-0.260	0.056	0.023	7135	3.60E-06	0.0030	21.49
Phosphatidylcholine (16:0_20:2) levels	rs62081852	18	26802732	T	C	0.389	0.068	0.018	7135	9.35E-09	0.0046	33.04
Phosphatidylcholine (16:0_20:2) levels	rs150057262	19	19210016	G	C	-0.202	0.039	0.052	7135	1.77E-07	0.0038	27.32
Phosphatidylcholine (16:0_20:2) levels	rs138597	22	44572650	A	G	0.081	0.018	0.709	7135	9.82E-06	0.0027	19.57
Phosphatidylcholine (16:0_20:4) levels	rs116643980	1	4847659	T	G	0.167	0.037	0.056	7174	4.84E-06	0.0029	20.92
Phosphatidylcholine (16:0_20:4) levels	rs3820438	1	201955621	C	G	0.089	0.018	0.291	7174	1.15E-06	0.0033	23.70
Phosphatidylcholine (16:0_20:4) levels	rs17366743	3	186854300	C	T	-0.203	0.046	0.034	7174	9.68E-06	0.0027	19.59
Phosphatidylcholine (16:0_20:4) levels	rs17017629	4	143352205	T	C	-0.231	0.051	0.027	7174	6.78E-06	0.0028	20.28
Phosphatidylcholine (16:0_20:4) levels	rs116438525	4	181645150	T	C	-0.470	0.102	0.007	7174	4.05E-06	0.0030	21.27
Phosphatidylcholine (16:0_20:4) levels	rs149130695	4	186464194	C	A	0.154	0.035	0.065	7174	9.36E-06	0.0027	19.66
Phosphatidylcholine (16:0_20:4) levels	rs2966427	7	110566718	G	C	-0.147	0.033	0.932	7174	7.78E-06	0.0028	20.01
Phosphatidylcholine (16:0_20:4)	rs581608	7	156099813	T	C	0.09	0.02	0.22	717	5.72E-	0.002	20.60

levels	59					3	0	1	4	06	9	
Phosphatidylcholine (16:0_20:4) levels	rs1251003	8	8854096	A	T	-0.144	0.030	0.090	7174	1.23E-06	0.0033	23.56
Phosphatidylcholine (16:0_20:4) levels	rs4756323	11	36469520	T	C	-0.078	0.017	0.456	7174	3.22E-06	0.0030	21.71
Phosphatidylcholine (16:0_20:4) levels	rs2943813	11	61490996	C	T	-0.139	0.020	0.783	7174	9.96E-12	0.0064	46.46
Phosphatidylcholine (16:0_20:4) levels	rs174527	11	61770929	C	G	-0.179	0.017	0.444	7174	8.38E-26	0.0153	111.16
Phosphatidylcholine (16:0_20:4) levels	rs174533	11	61781553	A	G	-0.598	0.016	0.409	7174	6.29E-295	0.1711	1480.88
Phosphatidylcholine (16:0_20:4) levels	rs74754540	11	61948885	A	G	-0.179	0.039	0.049	7174	4.19E-06	0.0029	21.20
Phosphatidylcholine (16:0_20:4) levels	rs113394924	11	62314821	G	T	0.214	0.030	0.087	7174	6.09E-13	0.0072	52.00
Phosphatidylcholine (16:0_20:4) levels	rs3018617	11	62435462	G	A	-0.108	0.019	0.302	7174	8.62E-09	0.0046	33.20
Phosphatidylcholine (16:0_20:4) levels	rs499974	11	75743976	A	C	-0.103	0.020	0.232	7174	2.26E-07	0.0037	26.85
Phosphatidylcholine (16:0_20:4) levels	rs11062107	12	2077875	G	A	-0.089	0.020	0.223	7174	9.12E-06	0.0027	19.71
Phosphatidylcholine (16:0_20:4) levels	rs72709529	14	101397558	A	C	-0.27	0.056	0.023	7174	1.13E-06	0.0033	23.73

						2						
Phosphatidylcholine (16:0_20:4) levels	rs147549994	16	1939264	T	G	0.246	0.045	0.036	7174	5.51E-08	0.0041	29.58
Phosphatidylcholine (16:0_20:4) levels	rs6498540	16	15036737	G	A	-0.079	0.018	0.334	7174	8.47E-06	0.0028	19.85
Phosphatidylcholine (16:0_20:4) levels	rs12720922	16	56966973	A	G	-0.096	0.022	0.179	7174	9.09E-06	0.0027	19.72
Phosphatidylcholine (16:0_20:4) levels	rs13329893	16	87672930	C	A	0.158	0.036	0.060	7174	9.16E-06	0.0027	19.70
Phosphatidylcholine (16:0_20:4) levels	rs141391095	17	10763022	G	A	-0.281	0.060	0.021	7174	2.58E-06	0.0031	22.13
Phosphatidylcholine (16:0_20:4) levels	rs187429064	19	19269704	G	A	-0.226	0.038	0.054	7174	2.04E-09	0.0050	36.01
Phosphatidylcholine (16:0_20:5) levels	rs4908782	1	8828055	T	C	-0.080	0.017	0.354	7173	5.17E-06	0.0029	20.80
Phosphatidylcholine (16:0_20:5) levels	rs34822092	2	52123601	A	T	-0.283	0.056	0.023	7173	5.21E-07	0.0035	25.22
Phosphatidylcholine (16:0_20:5) levels	rs3936196	2	239878629	A	G	-0.077	0.017	0.609	7173	9.25E-06	0.0027	19.68
Phosphatidylcholine (16:0_20:5) levels	rs59839417	3	39249420	A	G	-0.099	0.021	0.206	7173	2.02E-06	0.0031	22.60
Phosphatidylcholine (16:0_20:5) levels	rs2733412	3	114428196	C	T	-0.096	0.021	0.189	7173	8.72E-06	0.0028	19.80

Phosphatidylcholine (16:0_20:5) levels	rs10936925	3	177015370	T	G	0.079	0.018	0.340	7173	8.89E-06	0.0027	19.76
Phosphatidylcholine (16:0_20:5) levels	rs3796575	4	155800587	T	C	-0.092	0.020	0.228	7173	6.46E-06	0.0028	20.37
Phosphatidylcholine (16:0_20:5) levels	rs146489454	5	115424684	T	C	-0.217	0.049	0.031	7173	9.32E-06	0.0027	19.67
Phosphatidylcholine (16:0_20:5) levels	rs113917790	6	21142482	T	C	0.350	0.074	0.013	7173	2.55E-06	0.0031	22.16
Phosphatidylcholine (16:0_20:5) levels	rs7780677	7	132693734	A	C	-0.086	0.017	0.409	7173	4.33E-07	0.0036	25.58
Phosphatidylcholine (16:0_20:5) levels	rs7947954	11	42657198	C	T	0.108	0.023	0.155	7173	2.80E-06	0.0031	21.97
Phosphatidylcholine (16:0_20:5) levels	rs174527	11	61770929	C	G	-0.101	0.017	0.444	7173	3.21E-09	0.0049	35.13
Phosphatidylcholine (16:0_20:5) levels	rs102274	11	61790354	C	T	-0.373	0.017	0.409	7173	4.34E-109	0.0664	509.72
Phosphatidylcholine (16:0_20:5) levels	rs74754540	11	61948885	A	G	-0.189	0.039	0.049	7173	1.18E-06	0.0033	23.64
Phosphatidylcholine (16:0_20:5) levels	rs113394924	11	62314821	G	T	0.141	0.030	0.087	7173	2.28E-06	0.0031	22.38
Phosphatidylcholine (16:0_20:5) levels	rs3018617	11	62435462	G	A	-0.093	0.019	0.302	7173	7.08E-07	0.0034	24.62
Phosphatidylcholine (16:0_20:5) levels	rs531117	11	75745089	T	C	-0.088	0.020	0.232	7173	8.73E-06	0.0028	19.80

Phosphatidylcholine (16:0_20:5) levels	rs28569162	11	87975724	G	A	0.115	0.025	0.131	7173	4.68E-06	0.0029	20.99
Phosphatidylcholine (16:0_20:5) levels	rs1481977	11	103598670	G	A	0.109	0.024	0.861	7173	7.66E-06	0.0028	20.04
Phosphatidylcholine (16:0_20:5) levels	rs2041149	12	101716327	G	A	-0.079	0.017	0.477	7173	2.73E-06	0.0031	22.03
Phosphatidylcholine (16:0_20:5) levels	rs137945273	14	71270831	T	C	-0.204	0.045	0.037	7173	7.22E-06	0.0028	20.16
Phosphatidylcholine (16:0_20:5) levels	rs80083325	14	104397326	A	G	-0.214	0.048	0.035	7173	8.69E-06	0.0028	19.80
Phosphatidylcholine (16:0_20:5) levels	rs62025936	16	10434179	G	A	0.121	0.023	0.165	7173	1.18E-07	0.0039	28.10
Phosphatidylcholine (16:0_20:5) levels	rs61653779	16	20568904	C	T	0.274	0.060	0.020	7173	5.99E-06	0.0029	20.52
Phosphatidylcholine (16:0_20:5) levels	rs2833287	21	31127778	G	A	0.460	0.103	0.007	7173	8.62E-06	0.0028	19.82
Phosphatidylcholine (17:0_18:1) levels	rs17030373	1	7149275	G	A	0.083	0.019	0.290	7073	9.60E-06	0.0028	19.61
Phosphatidylcholine (17:0_18:1) levels	rs139070595	1	247024993	A	T	0.592	0.122	0.005	7073	1.35E-06	0.0033	23.38
Phosphatidylcholine (17:0_18:1) levels	rs1257173	2	134188357	C	G	-0.134	0.030	0.088	7073	7.90E-06	0.0028	19.98
Phosphatidylcholine (17:0_18:1) levels	rs145967151	2	241739239	T	C	-0.114	0.026	0.129	7073	9.92E-06	0.0028	19.55
Phosphatidylcholine (17:0_18:1) levels	rs5022521	4	110207431	T	C	0.096	0.021	0.797	7073	6.55E-06	0.0029	20.34
Phosphatidylcholine (17:0_18:1)	rs775347	4	130689035	A	C	-	0.04	0.03	707	4.12E-	0.003	21.23

levels	89					0.208	5	8	3	06	0	
Phosphatidylcholine (17:0_18:1) levels	rs4692808	4	170196755	A	G	0.080	0.017	0.436	7073	2.08E-06	0.0032	22.55
Phosphatidylcholine (17:0_18:1) levels	rs1106933	6	166616136	G	A	0.125	0.024	0.136	7073	3.44E-07	0.0037	26.03
Phosphatidylcholine (17:0_18:1) levels	rs2126263	8	9324101	A	G	0.123	0.024	0.850	7073	1.95E-07	0.0038	27.12
Phosphatidylcholine (17:0_18:1) levels	rs187552376	8	93920504	C	T	-0.318	0.070	0.016	7073	6.09E-06	0.0029	20.48
Phosphatidylcholine (17:0_18:1) levels	rs78308012	9	137586795	G	T	0.153	0.029	0.103	7073	1.11E-07	0.0040	28.22
Phosphatidylcholine (17:0_18:1) levels	rs143506888	12	14115139	T	A	0.296	0.064	0.018	7073	4.13E-06	0.0030	21.23
Phosphatidylcholine (17:0_18:1) levels	rs76157703	13	78203799	T	G	-0.324	0.071	0.015	7073	5.78E-06	0.0029	20.58
Phosphatidylcholine (17:0_18:1) levels	rs7350789	15	58387469	A	G	0.113	0.017	0.393	7073	4.46E-11	0.0061	43.52
Phosphatidylcholine (17:0_18:1) levels	rs17231506	16	56960616	T	C	0.094	0.019	0.278	7073	5.82E-07	0.0035	25.01
Phosphatidylcholine (17:0_18:1) levels	rs12373089	16	79172686	A	G	0.112	0.025	0.135	7073	8.44E-06	0.0028	19.86
Phosphatidylcholine (17:0_18:1) levels	rs61737926	16	87644718	T	C	-0.754	0.164	0.003	7073	4.10E-06	0.0030	21.24
Phosphatidylcholine (17:0_18:1) levels	rs141245990	18	44024688	T	C	-0.247	0.054	0.026	7073	3.97E-06	0.0030	21.31
Phosphatidylcholine (17:0_18:1) levels	rs2003890	20	61233003	G	A	-0.09	0.020	0.223	7073	4.57E-06	0.0030	21.03

						3						
Phosphatidylcholine (17:0_18:1) levels	rs140419225	20	61849802	A	G	0.646	0.139	0.004	7073	3.40E-06	0.0030	21.61
Phosphatidylcholine (17:0_20:4) levels	rs116643980	1	4847659	T	G	0.169	0.037	0.056	7106	4.65E-06	0.0029	21.00
Phosphatidylcholine (17:0_20:4) levels	rs143309746	1	27599934	G	A	0.270	0.059	0.023	7106	4.94E-06	0.0029	20.88
Phosphatidylcholine (17:0_20:4) levels	rs1168041	1	62494579	C	T	0.088	0.019	0.733	7106	2.75E-06	0.0031	22.01
Phosphatidylcholine (17:0_20:4) levels	rs35788378	1	236035822	T	A	0.085	0.019	0.262	7106	5.52E-06	0.0029	20.67
Phosphatidylcholine (17:0_20:4) levels	rs190664666	2	142929648	A	G	-0.158	0.035	0.064	7106	5.01E-06	0.0029	20.86
Phosphatidylcholine (17:0_20:4) levels	rs55725503	3	66040764	A	G	-0.248	0.055	0.025	7106	5.71E-06	0.0029	20.61
Phosphatidylcholine (17:0_20:4) levels	rs3110971	5	38490169	G	A	0.099	0.021	0.208	7106	1.78E-06	0.0032	22.85
Phosphatidylcholine (17:0_20:4) levels	rs56399423	5	132336964	C	T	0.084	0.018	0.323	7106	2.49E-06	0.0031	22.21
Phosphatidylcholine (17:0_20:4) levels	rs142082962	6	158517411	A	C	-0.144	0.031	0.078	7106	3.46E-06	0.0030	21.57
Phosphatidylcholine (17:0_20:4) levels	rs73161879	7	152050140	A	G	-0.103	0.022	0.181	7106	3.77E-06	0.0030	21.41
Phosphatidylcholine (17:0_20:4) levels	rs143060591	8	85171256	T	C	-0.150	0.032	0.074	7106	3.72E-06	0.0030	21.43
Phosphatidylcholine (17:0_20:4) levels	rs585002	9	15293515	C	T	0.115	0.024	0.862	7106	2.33E-06	0.0031	22.33

Phosphatidylcholine (17:0_20:4) levels	rs117580248	9	137477951	A	G	0.131	0.028	0.110	7106	2.78E-06	0.0031	21.99
Phosphatidylcholine (17:0_20:4) levels	rs174527	11	61770929	C	G	-0.176	0.017	0.444	7106	1.14E-24	0.0147	105.86
Phosphatidylcholine (17:0_20:4) levels	rs174533	11	61781553	A	G	-0.543	0.016	0.409	7106	9.69E-236	0.1403	1159.58
Phosphatidylcholine (17:0_20:4) levels	rs57815521	11	61960675	T	C	-0.295	0.053	0.027	7106	2.07E-08	0.0044	31.49
Phosphatidylcholine (17:0_20:4) levels	rs140619100	11	62195501	G	A	0.218	0.035	0.062	7106	3.15E-10	0.0056	39.68
Phosphatidylcholine (17:0_20:4) levels	rs61896563	11	62267340	T	C	-0.188	0.040	0.048	7106	2.47E-06	0.0031	22.21
Phosphatidylcholine (17:0_20:4) levels	rs113394924	11	62314821	G	T	0.194	0.030	0.087	7106	8.01E-11	0.0059	42.38
Phosphatidylcholine (17:0_20:4) levels	rs600518	11	75744328	T	A	-0.113	0.020	0.232	7106	1.42E-08	0.0045	32.23
Phosphatidylcholine (17:0_20:4) levels	rs74452899	12	88630462	C	T	-0.446	0.088	0.011	7106	4.64E-07	0.0036	25.45
Phosphatidylcholine (17:0_20:4) levels	rs2398428	12	128751021	G	A	-0.097	0.020	0.263	7106	8.96E-07	0.0034	24.17
Phosphatidylcholine (17:0_20:4) levels	rs12429208	13	37767109	T	C	0.088	0.019	0.274	7106	3.76E-06	0.0030	21.41
Phosphatidylcholine (17:0_20:4) levels	rs11626405	14	81838369	A	G	-0.081	0.018	0.307	7106	7.26E-06	0.0028	20.15

Phosphatidylcholine (17:0_20:4) levels	rs12928399	16	14611144	C	G	-0.086	0.018	0.361	7106	1.08E-06	0.0033	23.81
Phosphatidylcholine (17:0_20:4) levels	rs635196	18	44179195	C	T	0.100	0.022	0.184	7106	3.91E-06	0.0030	21.33
Phosphatidylcholine (17:0_20:4) levels	rs2469035	18	73095106	G	A	-0.092	0.020	0.787	7106	6.69E-06	0.0029	20.31
Phosphatidylcholine (17:0_20:4) levels	rs187429064	19	19269704	G	A	-0.240	0.038	0.054	7106	3.18E-10	0.0056	39.66
Phosphatidylcholine (17:0_20:4) levels	rs11673120	19	47696062	A	G	0.076	0.017	0.419	7106	7.45E-06	0.0028	20.10
Phosphatidylcholine (17:0_20:4) levels	rs6083838	20	25397682	G	A	-0.078	0.017	0.536	7106	4.51E-06	0.0030	21.06
Phosphatidylcholine (17:0_20:4) levels	rs11088781	21	20132472	A	G	0.145	0.031	0.914	7106	2.27E-06	0.0031	22.38
Phosphatidylcholine (18:0_20:2) levels	rs111595890	1	214966966	A	G	-0.294	0.065	0.022	5778	6.17E-06	0.0035	20.46
Phosphatidylcholine (18:0_20:2) levels	rs6744368	2	68444883	T	C	0.188	0.043	0.948	5778	9.85E-06	0.0034	19.57
Phosphatidylcholine (18:0_20:2) levels	rs12513382	4	43912586	T	C	-0.088	0.019	0.621	5778	3.68E-06	0.0037	21.45
Phosphatidylcholine (18:0_20:2) levels	rs112997491	5	86253956	A	G	-0.199	0.044	0.048	5778	8.03E-06	0.0034	19.96
Phosphatidylcholine (18:0_20:2) levels	rs1422819	5	166671878	G	T	-0.090	0.019	0.593	5778	1.97E-06	0.0039	22.66

Phosphatidylcholine (18:0_20:2) levels	rs67207258	8	5603995	T	C	-0.105	0.024	0.190	5778	9.22E-06	0.0034	19.69
Phosphatidylcholine (18:0_20:2) levels	rs112483795	8	12981507	G	A	-0.290	0.062	0.024	5778	3.37E-06	0.0037	21.63
Phosphatidylcholine (18:0_20:2) levels	rs174527	11	61770929	C	G	0.102	0.019	0.444	5778	8.17E-08	0.0050	28.82
Phosphatidylcholine (18:0_20:2) levels	rs174547	11	61803311	C	T	0.282	0.019	0.407	5778	4.05E-51	0.0384	230.57
Phosphatidylcholine (18:0_20:2) levels	rs7959861	12	90339893	G	A	0.087	0.019	0.348	5778	8.23E-06	0.0034	19.91
Phosphatidylcholine (18:0_20:2) levels	rs7139079	12	120977490	A	G	-0.099	0.019	0.547	5778	1.17E-07	0.0048	28.13
Phosphatidylcholine (18:0_20:2) levels	rs17238104	13	37429471	T	C	-0.123	0.027	0.138	5778	7.01E-06	0.0035	20.22
Phosphatidylcholine (18:0_20:2) levels	rs147220276	13	73360255	G	T	0.427	0.095	0.010	5778	7.84E-06	0.0035	20.01
Phosphatidylcholine (18:0_20:2) levels	rs9671254	14	24946787	A	T	-0.095	0.021	0.711	5778	3.88E-06	0.0037	21.36
Phosphatidylcholine (18:0_20:2) levels	rs34955778	16	15045737	C	T	0.098	0.019	0.436	5778	2.01E-07	0.0047	27.08
Phosphatidylcholine (18:0_20:2) levels	rs8068923	17	54741041	A	G	0.190	0.040	0.940	5778	1.81E-06	0.0039	22.83
Phosphatidylcholine (18:0_20:2) levels	rs8183765	20	6660791	T	C	0.182	0.041	0.059	5778	8.04E-06	0.0034	19.96
Phosphatidylcholine (18:0_20:2) levels	rs6005648	22	27828806	G	A	-0.123	0.026	0.150	5778	3.25E-06	0.0037	21.69

Phosphatidylcholine (18:0_20:3) levels	rs116259186	1	74502464	C	T	0.227	0.051	0.029	7169	7.49E-06	0.0028	20.09
Phosphatidylcholine (18:0_20:3) levels	rs2797620	1	94975736	T	C	-0.116	0.018	0.333	7169	3.27E-11	0.0061	44.13
Phosphatidylcholine (18:0_20:3) levels	rs11577288	1	242606205	A	G	0.089	0.020	0.234	7169	7.14E-06	0.0028	20.18
Phosphatidylcholine (18:0_20:3) levels	rs13001426	2	169055662	A	C	0.335	0.074	0.014	7169	6.79E-06	0.0028	20.27
Phosphatidylcholine (18:0_20:3) levels	rs62239496	3	37451054	G	A	-0.094	0.021	0.201	7169	7.30E-06	0.0028	20.13
Phosphatidylcholine (18:0_20:3) levels	rs114162005	3	148308352	C	A	-0.179	0.039	0.053	7169	5.96E-06	0.0029	20.52
Phosphatidylcholine (18:0_20:3) levels	rs139676091	4	16403106	A	T	0.371	0.083	0.011	7169	7.59E-06	0.0028	20.06
Phosphatidylcholine (18:0_20:3) levels	rs78009561	4	158046658	G	C	-0.127	0.029	0.096	7169	9.94E-06	0.0027	19.54
Phosphatidylcholine (18:0_20:3) levels	rs140123208	5	16863519	G	A	-0.204	0.044	0.038	7169	3.45E-06	0.0030	21.57
Phosphatidylcholine (18:0_20:3) levels	rs192117305	5	33853083	T	C	-0.197	0.043	0.041	7169	4.38E-06	0.0029	21.11
Phosphatidylcholine (18:0_20:3) levels	rs72827678	5	168832803	G	T	0.143	0.030	0.086	7169	2.15E-06	0.0031	22.49
Phosphatidylcholine (18:0_20:3) levels	rs3134937	6	32238762	T	C	-0.095	0.021	0.200	7169	4.78E-06	0.0029	20.95
Phosphatidylcholine (18:0_20:3)	rs151178	9	130575137	A	G	0.28	0.06	0.01	716	2.48E-	0.003	22.21

levels	379					9	1	9	9	06	1	
Phosphatidylcholine (18:0_20:3) levels	rs56205329	9	133667068	A	G	-0.299	0.067	0.016	7169	8.55E-06	0.0028	19.83
Phosphatidylcholine (18:0_20:3) levels	rs7943728	11	61779596	A	G	0.236	0.027	0.112	7169	6.87E-19	0.0109	79.21
Phosphatidylcholine (18:0_20:3) levels	rs140588245	11	124328766	A	G	0.364	0.082	0.011	7169	8.52E-06	0.0028	19.84
Phosphatidylcholine (18:0_20:3) levels	rs112534228	12	6260820	C	A	0.210	0.045	0.038	7169	2.78E-06	0.0031	21.99
Phosphatidylcholine (18:0_20:3) levels	rs1169288	12	120978847	C	A	0.077	0.017	0.377	7169	6.39E-06	0.0028	20.39
Phosphatidylcholine (18:0_20:3) levels	rs11847009	14	33476504	A	T	-0.111	0.022	0.174	7169	6.87E-07	0.0034	24.69
Phosphatidylcholine (18:0_20:3) levels	rs59739041	15	58368884	C	T	0.183	0.039	0.048	7169	2.32E-06	0.0031	22.34
Phosphatidylcholine (18:0_20:3) levels	rs6498540	16	15036737	G	A	-0.199	0.018	0.334	7169	2.09E-29	0.0175	127.86
Phosphatidylcholine (18:0_20:3) levels	rs56374730	16	15773795	G	T	0.089	0.018	0.296	7169	1.44E-06	0.0032	23.25
Phosphatidylcholine (18:0_20:3) levels	rs11648397	16	78589971	C	G	0.152	0.032	0.078	7169	1.59E-06	0.0032	23.06
Phosphatidylcholine (18:0_20:3) levels	rs116917626	17	51561964	T	C	-0.236	0.052	0.028	7169	6.79E-06	0.0028	20.27
Phosphatidylcholine (18:0_20:3) levels	rs4808779	19	18294126	G	A	-0.088	0.018	0.666	7169	5.93E-07	0.0035	24.97
Phosphatidylcholine (18:0_20:3) levels	rs6141277	20	32243354	C	G	-0.07	0.017	0.493	7169	7.41E-06	0.0028	20.11

						5						
Phosphatidylcholine (18:0_20:3) levels	rs1800961	20	44413724	T	C	-0.184	0.037	0.052	7169	7.41E-07	0.0034	24.54
Phosphatidylcholine (18:0_20:3) levels	rs2379129	20	62310086	A	C	-0.081	0.017	0.399	7169	2.15E-06	0.0031	22.48
Phosphatidylcholine (18:0_20:4) levels	rs2797620	1	94975736	T	C	-0.101	0.017	0.333	7174	6.11E-09	0.0047	33.87
Phosphatidylcholine (18:0_20:4) levels	rs938822	3	64950744	C	T	-0.106	0.023	0.146	7174	4.66E-06	0.0029	21.00
Phosphatidylcholine (18:0_20:4) levels	rs3110971	5	38490169	G	A	0.096	0.020	0.208	7174	2.72E-06	0.0031	22.03
Phosphatidylcholine (18:0_20:4) levels	rs6860806	5	132304843	G	A	0.079	0.017	0.420	7174	2.78E-06	0.0031	21.99
Phosphatidylcholine (18:0_20:4) levels	rs9462860	6	42979275	C	G	0.076	0.017	0.458	7174	4.66E-06	0.0029	21.00
Phosphatidylcholine (18:0_20:4) levels	rs2990536	10	36620136	T	C	-0.078	0.017	0.578	7174	3.48E-06	0.0030	21.56
Phosphatidylcholine (18:0_20:4) levels	rs61873274	10	124924165	G	A	0.146	0.032	0.072	7174	5.07E-06	0.0029	20.83
Phosphatidylcholine (18:0_20:4) levels	rs12275418	11	61012351	A	G	0.124	0.025	0.134	7174	4.66E-07	0.0035	25.44
Phosphatidylcholine (18:0_20:4) levels	rs3019200	11	61481911	A	C	-0.163	0.021	0.803	7174	2.57E-14	0.0081	58.26
Phosphatidylcholine (18:0_20:4) levels	rs79136768	11	61594967	G	A	-0.249	0.045	0.037	7174	2.71E-08	0.0043	30.97

Phosphatidylcholine (18:0_20:4) levels	rs3741252	11	61744026	T	C	0.243	0.024	0.134	7174	5.54E-23	0.0135	98.08
Phosphatidylcholine (18:0_20:4) levels	rs174528	11	61776027	C	T	-0.666	0.015	0.414	7174	0	0.2134	1945.60
Phosphatidylcholine (18:0_20:4) levels	rs147981159	11	62056826	A	G	-0.346	0.042	0.043	7174	1.54E-16	0.0095	68.42
Phosphatidylcholine (18:0_20:4) levels	rs4382917	11	62454004	A	G	0.149	0.020	0.256	7174	4.68E-14	0.0079	57.06
Phosphatidylcholine (18:0_20:4) levels	rs1219539	11	75722099	A	G	-0.088	0.017	0.493	7174	1.74E-07	0.0038	27.35
Phosphatidylcholine (18:0_20:4) levels	rs600518	11	75744328	T	A	-0.101	0.020	0.232	7174	3.49E-07	0.0036	26.00
Phosphatidylcholine (18:0_20:4) levels	rs2802390	13	98690376	T	C	-0.075	0.017	0.465	7174	7.51E-06	0.0028	20.08
Phosphatidylcholine (18:0_20:4) levels	rs139102782	14	83647468	A	G	-0.206	0.046	0.034	7174	9.06E-06	0.0027	19.72
Phosphatidylcholine (18:0_20:4) levels	rs2070895	15	58431740	A	G	0.094	0.019	0.267	7174	8.73E-07	0.0034	24.23
Phosphatidylcholine (18:0_20:4) levels	rs147549994	16	1939264	T	G	0.256	0.045	0.036	7174	1.35E-08	0.0045	32.31
Phosphatidylcholine (18:0_20:4) levels	rs72789541	16	15033677	A	T	-0.097	0.018	0.315	7174	6.36E-08	0.0041	29.30
Phosphatidylcholine (18:0_20:4) levels	rs7499892	16	56972678	T	C	-0.096	0.022	0.177	7174	9.21E-06	0.0027	19.70

Phosphatidylcholine (18:0_20:4) levels	rs223841	16	57423359	G	A	-0.103	0.022	0.172	7174	2.89E-06	0.0030	21.92
Phosphatidylcholine (18:0_20:4) levels	rs1477491	18	73092300	G	A	-0.102	0.020	0.789	7174	5.06E-07	0.0035	25.28
Phosphatidylcholine (18:0_20:4) levels	rs793864	19	7552489	C	G	-0.104	0.022	0.818	7174	1.50E-06	0.0032	23.17
Phosphatidylcholine (18:0_20:4) levels	rs73005469	19	15845766	A	G	-0.077	0.017	0.357	7174	9.40E-06	0.0027	19.65
Phosphatidylcholine (18:0_20:4) levels	rs187429064	19	19269704	G	A	-0.202	0.037	0.054	7174	7.42E-08	0.0040	29.00
Phosphatidylcholine (18:1_18:1) levels	rs10889352	1	62633352	C	T	-0.112	0.019	0.262	7174	2.61E-09	0.0049	35.53
Phosphatidylcholine (18:1_18:1) levels	rs41391244	1	111314654	C	T	-0.093	0.020	0.220	7174	3.80E-06	0.0030	21.39
Phosphatidylcholine (18:1_18:1) levels	rs10779836	1	230167404	T	C	0.092	0.020	0.777	7174	4.12E-06	0.0030	21.24
Phosphatidylcholine (18:1_18:1) levels	rs4848460	2	117641775	T	C	0.175	0.038	0.050	7174	5.05E-06	0.0029	20.84
Phosphatidylcholine (18:1_18:1) levels	rs56057593	2	204579749	T	C	-0.227	0.049	0.032	7174	4.30E-06	0.0029	21.15
Phosphatidylcholine (18:1_18:1) levels	rs140268092	4	29977517	T	C	0.582	0.128	0.005	7174	5.51E-06	0.0029	20.67
Phosphatidylcholine (18:1_18:1) levels	rs73241068	4	38036537	T	C	-0.21	0.046	0.034	7174	1.85E-06	0.0032	22.78

						8						
Phosphatidylcholine (18:1_18:1) levels	rs79008894	4	140097358	T	C	0.209	0.046	0.039	7174	5.56E-06	0.0029	20.66
Phosphatidylcholine (18:1_18:1) levels	rs41441450	5	86717236	T	C	-0.394	0.085	0.010	7174	3.92E-06	0.0030	21.33
Phosphatidylcholine (18:1_18:1) levels	rs113277188	5	151601186	C	G	-0.291	0.066	0.018	7174	9.15E-06	0.0027	19.70
Phosphatidylcholine (18:1_18:1) levels	rs144512980	6	18685527	C	T	0.370	0.069	0.015	7174	8.17E-08	0.0040	28.82
Phosphatidylcholine (18:1_18:1) levels	rs2126263	8	9324101	A	G	0.112	0.023	0.850	7174	1.93E-06	0.0032	22.69
Phosphatidylcholine (18:1_18:1) levels	rs13278111	8	22702678	T	C	-0.077	0.017	0.430	7174	7.00E-06	0.0028	20.22
Phosphatidylcholine (18:1_18:1) levels	rs174574	11	61832870	C	A	-0.202	0.017	0.591	7174	1.28E-32	0.0195	142.82
Phosphatidylcholine (18:1_18:1) levels	rs3741298	11	116786845	T	C	-0.089	0.020	0.765	7174	5.19E-06	0.0029	20.79
Phosphatidylcholine (18:1_18:1) levels	rs10850057	12	112553648	T	C	-0.125	0.028	0.100	7174	8.27E-06	0.0028	19.90
Phosphatidylcholine (18:1_18:1) levels	rs7300883	12	121021637	G	C	-0.076	0.017	0.438	7174	6.70E-06	0.0028	20.30
Phosphatidylcholine (18:1_18:1) levels	rs10145681	14	68688212	G	A	-0.267	0.057	0.976	7174	2.32E-06	0.0031	22.34
Phosphatidylcholine (18:1_18:1)	rs153208	15	58391167	G	A	-	0.01	0.57	717	7.42E-	0.005	42.52

levels	5					0.110	7	3	4	11	9	
Phosphatidylcholine (18:1_18:1) levels	rs1077834	15	58431280	C	T	0.093	0.019	0.266	7174	1.56E-06	0.0032	23.10
Phosphatidylcholine (18:1_18:1) levels	rs17231506	16	56960616	T	C	0.109	0.019	0.278	7174	4.04E-09	0.0048	34.68
Phosphatidylcholine (18:1_18:1) levels	rs2000827	18	49695288	A	T	0.122	0.027	0.894	7174	7.27E-06	0.0028	20.14
Phosphatidylcholine (18:1_18:1) levels	rs136061	22	46945349	G	C	0.089	0.019	0.263	7174	2.41E-06	0.0031	22.26
Phosphatidylcholine (18:1_18:2) levels	rs10889352	1	62633352	C	T	-0.126	0.019	0.262	7174	1.75E-11	0.0063	45.37
Phosphatidylcholine (18:1_18:2) levels	rs9789400	2	57690087	T	C	-0.089	0.019	0.265	7174	2.74E-06	0.0031	22.02
Phosphatidylcholine (18:1_18:2) levels	rs28844909	4	145707221	A	G	-0.153	0.034	0.067	7174	7.07E-06	0.0028	20.20
Phosphatidylcholine (18:1_18:2) levels	rs13128807	4	169311729	A	G	-0.101	0.021	0.196	7174	1.81E-06	0.0032	22.81
Phosphatidylcholine (18:1_18:2) levels	rs78882831	5	96128576	T	C	0.243	0.054	0.025	7174	8.43E-06	0.0028	19.86
Phosphatidylcholine (18:1_18:2) levels	rs142689129	6	18587903	T	C	0.580	0.116	0.005	7174	6.42E-07	0.0034	24.82
Phosphatidylcholine (18:1_18:2) levels	rs11757990	6	29796264	T	C	0.457	0.103	0.007	7174	9.55E-06	0.0027	19.62
Phosphatidylcholine (18:1_18:2) levels	rs147761802	7	5810475	C	G	-0.181	0.039	0.050	7174	4.04E-06	0.0030	21.27
Phosphatidylcholine (18:1_18:2)	rs703655	9	14382621	C	T	0.09	0.01	0.35	717	2.43E-	0.003	26.70

levels	4					1	8	0	4	07	7	
Phosphatidylcholine (18:1_18:2) levels	rs174527	11	61770929	C	G	0.091	0.017	0.444	7174	1.11E-07	0.0039	28.21
Phosphatidylcholine (18:1_18:2) levels	rs174574	11	61832870	C	A	-0.380	0.016	0.591	7174	2.79E-114	0.0694	535.12
Phosphatidylcholine (18:1_18:2) levels	rs118108980	11	62211023	G	A	-0.100	0.021	0.196	7174	1.93E-06	0.0032	22.70
Phosphatidylcholine (18:1_18:2) levels	rs4944732	11	72735137	T	C	0.109	0.024	0.135	7174	8.27E-06	0.0028	19.90
Phosphatidylcholine (18:1_18:2) levels	rs12798268	11	107869185	C	A	-0.107	0.024	0.859	7174	7.19E-06	0.0028	20.16
Phosphatidylcholine (18:1_18:2) levels	rs613808	11	116840252	G	A	-0.091	0.017	0.619	7174	1.43E-07	0.0039	27.73
Phosphatidylcholine (18:1_18:2) levels	rs564216	11	126095437	C	T	-0.168	0.037	0.055	7174	4.51E-06	0.0029	21.06
Phosphatidylcholine (18:1_18:2) levels	rs1601935	15	58379566	T	G	-0.106	0.017	0.611	7174	5.31E-10	0.0054	38.66
Phosphatidylcholine (18:1_18:2) levels	rs1077835	15	58431227	G	A	0.125	0.019	0.266	7174	7.94E-11	0.0059	42.39
Phosphatidylcholine (18:1_18:2) levels	rs62004153	15	59198971	A	G	-0.131	0.030	0.913	7174	9.20E-06	0.0027	19.69
Phosphatidylcholine (18:1_18:2) levels	rs6498540	16	15036737	G	A	0.079	0.018	0.334	7174	7.68E-06	0.0028	20.04
Phosphatidylcholine (18:1_18:2) levels	rs17231506	16	56960616	T	C	0.120	0.018	0.278	7174	1.06E-10	0.0058	41.82

Phosphatidylcholine (18:1_18:2) levels	rs948727	18	13715335	G	A	-0.082	0.017	0.325	7174	2.78E-06	0.0031	21.99
Phosphatidylcholine (18:1_18:2) levels	rs6023985	20	55260213	C	T	-0.333	0.072	0.014	7174	4.10E-06	0.0030	21.24
Phosphatidylcholine (18:1_18:2) levels	rs2236605	21	37563977	C	G	0.084	0.017	0.432	7174	5.14E-07	0.0035	25.25
Phosphatidylcholine (O-16:0_18:1) levels	rs606587	1	230180443	G	A	-0.082	0.017	0.374	6956	2.49E-06	0.0032	22.21
Phosphatidylcholine (O-16:0_18:1) levels	rs876969	2	48551738	T	C	-0.111	0.024	0.150	6956	2.40E-06	0.0032	22.28
Phosphatidylcholine (O-16:0_18:1) levels	rs9682490	3	4949091	C	T	-0.080	0.017	0.435	6956	3.82E-06	0.0031	21.38
Phosphatidylcholine (O-16:0_18:1) levels	rs7677028	4	137022381	G	C	0.393	0.072	0.014	6956	5.29E-08	0.0042	29.66
Phosphatidylcholine (O-16:0_18:1) levels	rs2963775	5	115413799	C	G	0.079	0.018	0.581	6956	9.87E-06	0.0028	19.56
Phosphatidylcholine (O-16:0_18:1) levels	rs13194791	6	31307500	G	A	0.112	0.024	0.145	6956	3.55E-06	0.0031	21.52
Phosphatidylcholine (O-16:0_18:1) levels	rs7845596	8	2845634	G	C	0.154	0.032	0.077	6956	1.59E-06	0.0033	23.06
Phosphatidylcholine (O-16:0_18:1) levels	rs34861396	8	31640951	A	G	0.087	0.018	0.299	6956	2.52E-06	0.0032	22.18
Phosphatidylcholine (O-16:0_18:1) levels	rs138038919	8	118920650	T	C	0.089	0.020	0.248	6956	7.10E-06	0.0029	20.19
Phosphatidylcholine (O-16:0_18:1) levels	rs10120342	9	26919609	G	T	0.092	0.021	0.208	6956	8.42E-06	0.0028	19.86
Phosphatidylcholine (O-	rs772118	11	12450600	C	T	0.18	0.04	0.04	695	4.83E-	0.003	20.93

16:0_18:1) levels	23					9	1	9	6	06	0	
Phosphatidylcholine (O-16:0_18:1) levels	rs72923423	11	35044759	T	G	-0.152	0.033	0.074	6956	4.17E-06	0.0030	21.21
Phosphatidylcholine (O-16:0_18:1) levels	rs11057853	12	124844767	C	T	-0.077	0.017	0.454	6956	6.20E-06	0.0029	20.45
Phosphatidylcholine (O-16:0_18:1) levels	rs9538944	13	60820833	A	C	0.143	0.031	0.082	6956	5.62E-06	0.0030	20.64
Phosphatidylcholine (O-16:0_18:1) levels	rs1532085	15	58391167	G	A	-0.098	0.017	0.573	6956	9.47E-09	0.0047	33.01
Phosphatidylcholine (O-16:0_18:1) levels	rs1800588	15	58431476	T	C	0.119	0.020	0.258	6956	1.29E-09	0.0053	36.92
Phosphatidylcholine (O-16:0_18:1) levels	rs17231506	16	56960616	T	C	0.129	0.019	0.278	6956	6.16E-12	0.0068	47.43
Phosphatidylcholine (O-16:0_18:1) levels	rs117449635	16	71063316	A	G	0.333	0.072	0.015	6956	3.83E-06	0.0031	21.37
Phosphatidylcholine (O-16:0_18:1) levels	rs79037957	16	72797212	C	G	0.219	0.048	0.035	6956	4.45E-06	0.0030	21.08
Phosphatidylcholine (O-16:0_18:1) levels	rs113746682	16	85344145	T	C	0.273	0.059	0.023	6956	4.12E-06	0.0030	21.24
Phosphatidylcholine (O-16:0_18:1) levels	rs73015021	19	11082239	G	A	-0.137	0.029	0.092	6956	2.92E-06	0.0031	21.89
Phosphatidylcholine (O-16:0_18:1) levels	rs7260891	20	11603340	A	G	-0.079	0.017	0.394	6956	5.06E-06	0.0030	20.84
Phosphatidylcholine (O-16:0_18:1) levels	rs6023985	20	55260213	C	T	-0.326	0.073	0.014	6956	8.23E-06	0.0029	19.91
Phosphatidylcholine (O-	rs183335	2	71103693	T	C	0.09	0.02	0.25	670	7.62E-	0.003	20.05

16:0_20:3) levels	0					1	0	2	9	06	0	
Phosphatidylcholine (O-16:0_20:3) levels	rs75111872	3	134061016	A	G	-0.109	0.023	0.162	6709	2.93E-06	0.0033	21.89
Phosphatidylcholine (O-16:0_20:3) levels	rs10951096	7	3168526	G	A	-0.087	0.020	0.725	6709	8.90E-06	0.0029	19.76
Phosphatidylcholine (O-16:0_20:3) levels	rs12671763	7	28483085	A	C	0.311	0.069	0.017	6709	6.85E-06	0.0030	20.26
Phosphatidylcholine (O-16:0_20:3) levels	rs968742	7	29412521	G	A	0.081	0.017	0.519	6709	2.68E-06	0.0033	22.06
Phosphatidylcholine (O-16:0_20:3) levels	rs62501991	8	15196227	T	C	-0.101	0.022	0.180	6709	7.35E-06	0.0030	20.12
Phosphatidylcholine (O-16:0_20:3) levels	rs2360706	9	420203	C	G	0.091	0.020	0.766	6709	7.69E-06	0.0030	20.04
Phosphatidylcholine (O-16:0_20:3) levels	rs117435540	10	124011070	A	G	0.529	0.114	0.007	6709	3.69E-06	0.0032	21.45
Phosphatidylcholine (O-16:0_20:3) levels	rs968567	11	61828092	T	C	0.271	0.027	0.113	6709	2.17E-23	0.0147	100.02
Phosphatidylcholine (O-16:0_20:3) levels	rs75085081	11	118930336	A	G	0.238	0.053	0.028	6709	8.47E-06	0.0030	19.85
Phosphatidylcholine (O-16:0_20:3) levels	rs9552958	13	23426939	T	C	-0.136	0.030	0.095	6709	5.66E-06	0.0031	20.62
Phosphatidylcholine (O-16:0_20:3) levels	rs79036443	13	52780240	C	A	-0.197	0.043	0.042	6709	5.65E-06	0.0031	20.63
Phosphatidylcholine (O-16:0_20:3) levels	rs1800588	15	58431476	T	C	0.096	0.020	0.258	6709	1.73E-06	0.0034	22.91
Phosphatidylcholine (O-16:0_20:3) levels	rs6498540	16	15036737	G	A	-0.15	0.018	0.334	6709	5.73E-17	0.0104	70.41

						4						
Phosphatidylcholine (O-16:0_20:3) levels	rs7412	19	44908822	T	C	0.225	0.038	0.053	6709	4.52E-09	0.0051	34.46
Phosphatidylcholine (O-16:0_20:3) levels	rs6015087	20	57822739	C	T	0.078	0.018	0.421	6709	9.71E-06	0.0029	19.59
Phosphatidylcholine (O-16:1_18:0) levels	rs115012576	3	150051286	G	A	-0.507	0.114	0.006	6751	8.66E-06	0.0029	19.81
Phosphatidylcholine (O-16:1_18:0) levels	rs9997698	4	141036586	C	T	-0.177	0.038	0.056	6751	2.73E-06	0.0033	22.02
Phosphatidylcholine (O-16:1_18:0) levels	rs74997551	6	109308440	C	T	-0.131	0.029	0.097	6751	5.75E-06	0.0030	20.59
Phosphatidylcholine (O-16:1_18:0) levels	rs143594930	8	26457702	T	C	-0.402	0.091	0.010	6751	9.56E-06	0.0029	19.62
Phosphatidylcholine (O-16:1_18:0) levels	rs1397106	8	77676794	G	T	-0.078	0.017	0.411	6751	6.65E-06	0.0030	20.32
Phosphatidylcholine (O-16:1_18:0) levels	rs11187130	10	80809816	T	C	-0.452	0.094	0.009	6751	1.78E-06	0.0034	22.85
Phosphatidylcholine (O-16:1_18:0) levels	rs1093316	10	94349017	C	T	-0.147	0.029	0.098	6751	4.68E-07	0.0038	25.43
Phosphatidylcholine (O-16:1_18:0) levels	rs4565878	11	3204472	A	G	-0.087	0.019	0.259	6751	8.27E-06	0.0029	19.90
Phosphatidylcholine (O-16:1_18:0) levels	rs12270739	11	36410769	A	G	-0.123	0.026	0.139	6751	1.60E-06	0.0034	23.05

Phosphatidylcholine (O-16:1_18:0) levels	rs77512406	11	66971292	T	C	-0.134	0.026	0.130	6751	2.65E-07	0.0039	26.53
Phosphatidylcholine (O-16:1_18:0) levels	rs181154147	12	69626170	T	C	0.221	0.049	0.032	6751	7.02E-06	0.0030	20.22
Phosphatidylcholine (O-16:1_18:0) levels	rs116883951	13	102563170	A	G	0.439	0.098	0.008	6751	8.38E-06	0.0029	19.88
Phosphatidylcholine (O-16:1_18:0) levels	rs1440391	14	48425602	A	G	0.080	0.018	0.630	6751	9.17E-06	0.0029	19.70
Phosphatidylcholine (O-16:1_18:0) levels	rs1667515	14	85227560	A	G	-0.087	0.019	0.701	6751	3.27E-06	0.0032	21.68
Phosphatidylcholine (O-16:1_18:0) levels	rs1355105	17	53585624	C	T	0.081	0.018	0.595	6751	4.95E-06	0.0031	20.88
Phosphatidylcholine (O-16:1_18:0) levels	rs1941562	18	41193890	G	A	-0.086	0.019	0.282	6751	6.51E-06	0.0030	20.35
Phosphatidylcholine (O-16:1_18:0) levels	rs448313	22	22014247	C	T	0.172	0.034	0.082	6751	6.67E-07	0.0037	24.74
Phosphatidylcholine (O-16:1_20:3) levels	rs1203148	1	35503298	G	A	0.093	0.021	0.240	6770	8.17E-06	0.0029	19.92
Phosphatidylcholine (O-16:1_20:3) levels	rs686843	1	60908464	A	G	0.082	0.018	0.480	6770	3.83E-06	0.0031	21.38
Phosphatidylcholine (O-16:1_20:3) levels	rs11883812	2	215392258	C	T	-0.220	0.041	0.049	6770	6.86E-08	0.0043	29.16
Phosphatidylcholine (O-16:1_20:3) levels	rs9872756	3	87569181	A	G	0.099	0.020	0.749	6770	6.28E-07	0.0037	24.87
Phosphatidylcholine (O-16:1_20:3) levels	rs9880183	3	188687875	C	T	-0.078	0.018	0.390	6770	9.86E-06	0.0029	19.56
Phosphatidylcholine (O-	rs379516	4	37858054	T	C	0.11	0.02	0.14	677	1.51E-	0.003	23.17

16:1_20:3) levels	9					9	5	4	0	06	4	
Phosphatidylcholine (O-16:1_20:3) levels	rs6817929	4	56204082	G	A	0.092	0.018	0.588	6770	1.90E-07	0.0040	27.18
Phosphatidylcholine (O-16:1_20:3) levels	rs6815866	4	95119717	C	G	-0.134	0.027	0.891	6770	1.03E-06	0.0035	23.91
Phosphatidylcholine (O-16:1_20:3) levels	rs58105955	7	74470522	G	A	0.134	0.029	0.095	6770	6.06E-06	0.0030	20.49
Phosphatidylcholine (O-16:1_20:3) levels	rs254868	7	112024749	G	T	-0.102	0.023	0.172	6770	8.91E-06	0.0029	19.75
Phosphatidylcholine (O-16:1_20:3) levels	rs34455876	7	130154739	A	G	-0.087	0.019	0.313	6770	3.40E-06	0.0032	21.60
Phosphatidylcholine (O-16:1_20:3) levels	rs7874842	9	16476973	A	G	0.099	0.022	0.190	6770	5.08E-06	0.0031	20.83
Phosphatidylcholine (O-16:1_20:3) levels	rs61896141	11	61788567	C	A	0.220	0.027	0.113	6770	3.61E-16	0.0098	66.75
Phosphatidylcholine (O-16:1_20:3) levels	rs55665216	12	7036085	T	C	0.152	0.032	0.080	6770	1.59E-06	0.0034	23.07
Phosphatidylcholine (O-16:1_20:3) levels	rs35556451	12	28686870	T	C	-0.226	0.047	0.036	6770	1.28E-06	0.0035	23.48
Phosphatidylcholine (O-16:1_20:3) levels	rs80320850	12	61219406	A	G	-0.179	0.039	0.052	6770	3.99E-06	0.0031	21.30
Phosphatidylcholine (O-16:1_20:3) levels	rs112516243	13	27037828	G	T	0.759	0.170	0.003	6770	8.27E-06	0.0029	19.90
Phosphatidylcholine (O-16:1_20:3) levels	rs9602749	13	85434789	G	T	0.114	0.025	0.147	6770	3.30E-06	0.0032	21.66
Phosphatidylcholine (O-16:1_20:3) levels	rs56142013	14	56431229	T	C	-0.34	0.076	0.014	6770	4.31E-06	0.0031	21.15

						9						
Phosphatidylcholine (O-16:1_20:3) levels	rs1077989	14	67509105	C	A	-0.121	0.017	0.468	6770	1.70E-12	0.0073	49.96
Phosphatidylcholine (O-16:1_20:3) levels	rs8033940	15	58432643	A	G	0.089	0.019	0.337	6770	1.62E-06	0.0034	23.04
Phosphatidylcholine (O-16:1_20:3) levels	rs6498540	16	15036737	G	A	-0.147	0.018	0.334	6770	8.85E-16	0.0095	64.96
Phosphatidylcholine (O-16:1_20:3) levels	rs144750125	17	15541920	T	C	0.259	0.055	0.024	6770	3.17E-06	0.0032	21.74
Phosphatidylcholine (O-16:1_20:3) levels	rs28399664	19	44821499	G	C	-0.214	0.046	0.038	6770	3.68E-06	0.0032	21.46
Phosphatidylcholine (O-16:1_20:3) levels	rs1800961	20	44413724	T	C	-0.174	0.038	0.052	6770	6.40E-06	0.0030	20.39
Phosphatidylcholine (O-16:1_20:4) levels	rs114325590	1	107442367	T	C	-0.458	0.091	0.011	6025	4.70E-07	0.0042	25.43
Phosphatidylcholine (O-16:1_20:4) levels	rs17268903	2	56518805	G	A	-0.126	0.028	0.121	6025	8.32E-06	0.0033	19.89
Phosphatidylcholine (O-16:1_20:4) levels	rs56303140	2	157798940	A	G	0.233	0.051	0.035	6025	4.65E-06	0.0035	21.01
Phosphatidylcholine (O-16:1_20:4) levels	rs6752137	2	181604439	A	G	-0.129	0.028	0.120	6025	3.46E-06	0.0036	21.58
Phosphatidylcholine (O-16:1_20:4) levels	rs5028238	2	217342965	A	G	-0.096	0.022	0.765	6025	7.68E-06	0.0033	20.04
Phosphatidylcholine (O-	rs138018	3	19181916	G	A	-	0.06	0.02	602	4.74E-	0.003	20.97

16:1_20:4) levels	897					0.299	5	2	5	06	5	
Phosphatidylcholine (O-16:1_20:4) levels	rs6776300	3	153579259	A	C	0.082	0.018	0.534	6025	8.20E-06	0.0033	19.92
Phosphatidylcholine (O-16:1_20:4) levels	rs10487877	7	80837985	G	C	0.209	0.043	0.047	6025	1.29E-06	0.0039	23.47
Phosphatidylcholine (O-16:1_20:4) levels	rs74854572	8	9367590	G	C	-0.394	0.087	0.012	6025	6.39E-06	0.0034	20.39
Phosphatidylcholine (O-16:1_20:4) levels	rs174527	11	61770929	C	G	-0.121	0.019	0.444	6025	7.84E-11	0.0070	42.43
Phosphatidylcholine (O-16:1_20:4) levels	rs174535	11	61783884	C	T	-0.404	0.018	0.409	6025	1.18E-107	0.0775	505.75
Phosphatidylcholine (O-16:1_20:4) levels	rs2015950	11	62058058	A	G	-0.134	0.027	0.137	6025	5.32E-07	0.0042	25.19
Phosphatidylcholine (O-16:1_20:4) levels	rs180360	11	116728272	G	A	0.088	0.019	0.324	6025	6.34E-06	0.0034	20.41
Phosphatidylcholine (O-16:1_20:4) levels	rs12579775	12	6976009	A	G	0.160	0.033	0.082	6025	1.05E-06	0.0039	23.88
Phosphatidylcholine (O-16:1_20:4) levels	rs71452092	12	28633010	T	C	-0.222	0.050	0.036	6025	8.89E-06	0.0033	19.76
Phosphatidylcholine (O-16:1_20:4) levels	rs7963111	12	124935624	T	G	-0.193	0.043	0.049	6025	5.86E-06	0.0034	20.56
Phosphatidylcholine (O-16:1_20:4) levels	rs1316133	14	67471921	A	G	-0.090	0.020	0.299	6025	8.71E-06	0.0033	19.80
Phosphatidylcholine (O-	rs107798	14	67509105	C	A	-	0.01	0.46	602	3.43E-	0.018	113.0

16:1_20:4) levels	9					0.192	8	8	5	26	4	9
Phosphatidylcholine (O-16:1_20:4) levels	rs1037782	15	29614744	C	T	-0.173	0.038	0.061	6025	5.26E-06	0.0034	20.77
Phosphatidylcholine (O-16:1_20:4) levels	rs2043085	15	58388755	C	T	-0.100	0.018	0.576	6025	4.75E-08	0.0049	29.88
Phosphatidylcholine (O-16:1_20:4) levels	rs12920974	16	56959113	T	G	-0.104	0.020	0.330	6025	1.16E-07	0.0047	28.15
Phosphatidylcholine (O-16:1_20:4) levels	rs71366199	17	15393870	G	A	0.118	0.026	0.148	6025	6.50E-06	0.0034	20.36
Phosphatidylcholine (O-16:1_20:4) levels	rs2431867	19	7570522	A	G	0.105	0.023	0.199	6025	7.79E-06	0.0033	20.02
Phosphatidylcholine (O-16:1_20:4) levels	rs73531625	19	18613744	T	C	-0.091	0.020	0.289	6025	4.83E-06	0.0035	20.94
Phosphatidylcholine (O-16:1_20:4) levels	rs79740876	20	1463662	A	G	0.197	0.043	0.048	6025	4.27E-06	0.0035	21.17
Phosphatidylcholine (O-18:0_14:0) levels	rs7566488	2	11801863	G	A	-0.118	0.026	0.140	6462	7.91E-06	0.0031	19.98
Phosphatidylcholine (O-18:0_14:0) levels	rs77444850	3	127976198	C	A	-0.271	0.059	0.024	6462	3.83E-06	0.0033	21.38
Phosphatidylcholine (O-18:0_14:0) levels	rs60635506	4	121735493	C	A	-0.321	0.072	0.017	6462	7.54E-06	0.0031	20.07
Phosphatidylcholine (O-18:0_14:0) levels	rs116304326	4	158808075	T	C	0.308	0.069	0.017	6462	7.93E-06	0.0031	19.98
Phosphatidylcholine (O-	rs105161	5	174236186	G	A	-	0.02	0.73	646	8.99E-	0.003	19.74

18:0_14:0) levels	13					0.089	0	3	2	06	0	
Phosphatidylcholine (O-18:0_14:0) levels	rs9365258	6	161187390	C	G	-0.120	0.024	0.837	6462	5.76E-07	0.0039	25.03
Phosphatidylcholine (O-18:0_14:0) levels	rs117246644	10	58404019	T	C	-0.442	0.090	0.010	6462	9.60E-07	0.0037	24.04
Phosphatidylcholine (O-18:0_14:0) levels	rs72835302	10	86168949	A	C	0.142	0.030	0.102	6462	3.09E-06	0.0034	21.79
Phosphatidylcholine (O-18:0_14:0) levels	rs79139024	14	95941109	C	G	-0.487	0.107	0.008	6462	4.96E-06	0.0032	20.88
Phosphatidylcholine (O-18:0_14:0) levels	rs111316958	15	76487930	T	C	-0.303	0.068	0.016	6462	8.95E-06	0.0030	19.75
Phosphatidylcholine (O-18:0_14:0) levels	rs146726414	16	78809113	T	G	0.312	0.063	0.020	6462	8.16E-07	0.0038	24.36
Phosphatidylcholine (O-18:0_14:0) levels	rs73975572	17	851904	G	A	-0.200	0.045	0.042	6462	7.98E-06	0.0031	19.97
Phosphatidylcholine (O-18:0_14:0) levels	rs72637380	17	16925824	T	C	-0.169	0.035	0.067	6462	1.84E-06	0.0035	22.79
Phosphatidylcholine (O-18:0_14:0) levels	rs116907632	17	81307290	A	G	0.706	0.150	0.004	6462	2.58E-06	0.0034	22.14
Phosphatidylcholine (O-18:0_14:0) levels	rs2115108	19	7899091	C	T	0.098	0.021	0.243	6462	1.73E-06	0.0035	22.90
Phosphatidylcholine (O-18:0_14:0) levels	rs35728084	20	17196017	T	C	-0.174	0.039	0.053	6462	7.53E-06	0.0031	20.08
Phosphatidylcholine (O-	rs812904	21	38363308	G	A	0.09	0.02	0.25	646	2.40E-	0.003	22.27

18:0_14:0) levels	9					6	0	2	2	06	4	
Phosphatidylcholine (O-18:0_16:1) levels	rs79014169	1	65877338	A	G	0.114	0.026	0.155	5959	9.29E-06	0.0033	19.68
Phosphatidylcholine (O-18:0_16:1) levels	rs55969585	1	117639417	T	G	0.209	0.046	0.046	5959	4.75E-06	0.0035	20.97
Phosphatidylcholine (O-18:0_16:1) levels	rs149012657	2	229375003	C	T	-0.188	0.037	0.065	5959	3.82E-07	0.0043	25.83
Phosphatidylcholine (O-18:0_16:1) levels	rs4547920	5	124738074	A	G	0.126	0.025	0.166	5959	4.26E-07	0.0043	25.62
Phosphatidylcholine (O-18:0_16:1) levels	rs10243115	7	49798317	T	C	0.088	0.019	0.392	5959	3.01E-06	0.0037	21.84
Phosphatidylcholine (O-18:0_16:1) levels	rs11009204	10	33061164	A	G	0.143	0.031	0.098	5959	4.56E-06	0.0035	21.05
Phosphatidylcholine (O-18:0_16:1) levels	rs603424	10	100315722	A	G	-0.181	0.029	0.121	5959	5.21E-10	0.0065	38.70
Phosphatidylcholine (O-18:0_16:1) levels	rs639060	10	100348766	G	A	0.219	0.049	0.037	5959	7.25E-06	0.0034	20.15
Phosphatidylcholine (O-18:0_16:1) levels	rs12575589	11	96017012	T	C	0.112	0.023	0.205	5959	1.17E-06	0.0040	23.67
Phosphatidylcholine (O-18:0_16:1) levels	rs10859795	12	94924181	G	A	-0.084	0.018	0.494	5959	5.50E-06	0.0035	20.69
Phosphatidylcholine (O-18:0_16:1) levels	rs74644051	13	27642024	A	G	-0.344	0.072	0.017	5959	2.04E-06	0.0038	22.60
Phosphatidylcholine (O-18:0_16:1) levels	rs1257588	14	99061905	G	A	-0.084	0.019	0.538	5959	6.86E-06	0.0034	20.26
Phosphatidylcholine (O-18:0_16:1) levels	rs12903358	15	97398226	A	G	-0.11	0.026	0.146	5959	9.03E-06	0.0033	19.73

						7						
Phosphatidylcholine (O-18:0_16:1) levels	rs2911458	16	88778601	T	C	-0.090	0.019	0.389	5959	1.54E-06	0.0039	23.13
Phosphatidylcholine (O-18:0_16:1) levels	rs13044736	20	48803368	G	A	0.085	0.019	0.398	5959	5.96E-06	0.0034	20.53
Phosphatidylcholine (O-18:0_20:4) levels	rs34737685	1	164956995	C	A	-0.078	0.017	0.538	6956	5.63E-06	0.0030	20.63
Phosphatidylcholine (O-18:0_20:4) levels	rs138202451	2	66991489	A	G	-0.576	0.111	0.006	6956	2.32E-07	0.0038	26.79
Phosphatidylcholine (O-18:0_20:4) levels	rs148462060	2	130169813	G	A	-0.261	0.056	0.024	6956	3.30E-06	0.0031	21.66
Phosphatidylcholine (O-18:0_20:4) levels	rs79856406	2	163582301	G	T	-0.125	0.028	0.102	6956	9.96E-06	0.0028	19.54
Phosphatidylcholine (O-18:0_20:4) levels	rs148840717	3	16036318	T	G	-0.198	0.045	0.037	6956	8.98E-06	0.0028	19.74
Phosphatidylcholine (O-18:0_20:4) levels	rs11954170	5	106783090	G	A	0.083	0.018	0.324	6956	6.07E-06	0.0029	20.49
Phosphatidylcholine (O-18:0_20:4) levels	rs4706511	6	72738363	G	A	-0.098	0.022	0.191	6956	9.96E-06	0.0028	19.54
Phosphatidylcholine (O-18:0_20:4) levels	rs143233558	7	64330243	A	G	0.149	0.028	0.102	6956	1.55E-07	0.0040	27.58
Phosphatidylcholine (O-18:0_20:4) levels	rs6992951	8	18172468	A	C	-0.082	0.017	0.390	6956	2.05E-06	0.0032	22.58
Phosphatidylcholine (O-	rs787111	9	79743146	A	G	0.18	0.04	0.05	695	4.51E-	0.003	21.06

18:0_20:4) levels	60					2	0	0	6	06	0	
Phosphatidylcholine (O-18:0_20:4) levels	rs3741252	11	61744026	T	C	0.150	0.025	0.134	6956	1.99E-09	0.0052	36.06
Phosphatidylcholine (O-18:0_20:4) levels	rs174536	11	61784455	C	A	-0.308	0.017	0.408	6956	2.28E-72	0.0455	331.18
Phosphatidylcholine (O-18:0_20:4) levels	rs117870830	11	120590088	G	A	-0.306	0.067	0.017	6956	5.47E-06	0.0030	20.69
Phosphatidylcholine (O-18:0_20:4) levels	rs34382810	12	2231632	C	A	0.086	0.018	0.365	6956	1.12E-06	0.0034	23.75
Phosphatidylcholine (O-18:0_20:4) levels	rs73074943	12	21873844	T	C	0.251	0.055	0.024	6956	5.54E-06	0.0030	20.67
Phosphatidylcholine (O-18:0_20:4) levels	rs11057853	12	124844767	C	T	-0.077	0.017	0.454	6956	6.11E-06	0.0029	20.48
Phosphatidylcholine (O-18:0_20:4) levels	rs9591400	13	51261013	T	A	0.120	0.026	0.118	6956	5.67E-06	0.0030	20.62
Phosphatidylcholine (O-18:0_20:4) levels	rs1800588	15	58431476	T	C	0.103	0.020	0.258	6956	1.56E-07	0.0039	27.56
Phosphatidylcholine (O-18:0_20:4) levels	rs4782783	16	83381927	C	T	0.125	0.027	0.888	6956	3.64E-06	0.0031	21.47
Phosphatidylcholine (O-18:0_20:4) levels	rs376516	17	4978481	G	C	-0.075	0.017	0.473	6956	8.56E-06	0.0028	19.83
Phosphatidylcholine (O-18:0_20:4) levels	rs145755646	17	59341291	G	C	0.160	0.035	0.065	6956	4.21E-06	0.0030	21.19
Phosphatidylcholine (O-18:0_20:4) levels	rs7412	19	44908822	T	C	0.194	0.038	0.053	6956	2.93E-07	0.0038	26.34
Phosphatidylcholine (O-18:1_20:3) levels	rs1925669	1	20394196	A	G	-0.113	0.025	0.128	6836	9.76E-06	0.0029	19.58

Phosphatidylcholine (O-18:1_20:3) levels	rs149022879	1	212057557	A	G	-0.228	0.051	0.030	6836	6.44E-06	0.0030	20.38
Phosphatidylcholine (O-18:1_20:3) levels	rs12239821	1	245438211	A	G	-0.130	0.028	0.108	6836	4.62E-06	0.0031	21.01
Phosphatidylcholine (O-18:1_20:3) levels	rs9843297	3	22449600	G	A	0.100	0.022	0.196	6836	4.75E-06	0.0031	20.96
Phosphatidylcholine (O-18:1_20:3) levels	rs2170790	3	25418634	C	T	0.628	0.130	0.005	6836	1.33E-06	0.0034	23.41
Phosphatidylcholine (O-18:1_20:3) levels	rs12659734	5	9303860	C	T	0.107	0.023	0.155	6836	5.21E-06	0.0030	20.79
Phosphatidylcholine (O-18:1_20:3) levels	rs1540881	7	18082952	T	C	0.083	0.018	0.540	6836	3.73E-06	0.0031	21.42
Phosphatidylcholine (O-18:1_20:3) levels	rs62493656	7	158353254	T	C	-0.214	0.046	0.036	6836	3.89E-06	0.0031	21.35
Phosphatidylcholine (O-18:1_20:3) levels	rs139594872	8	140933062	A	G	-0.206	0.046	0.035	6836	9.50E-06	0.0029	19.63
Phosphatidylcholine (O-18:1_20:3) levels	rs78183891	9	70856641	T	C	0.223	0.050	0.030	6836	7.43E-06	0.0029	20.10
Phosphatidylcholine (O-18:1_20:3) levels	rs1978125	11	2436436	A	G	0.103	0.023	0.196	6836	9.23E-06	0.0029	19.69
Phosphatidylcholine (O-18:1_20:3) levels	rs174548	11	61803876	G	C	0.259	0.017	0.384	6836	2.68E-49	0.0314	221.26
Phosphatidylcholine (O-18:1_20:3) levels	rs11236557	11	75841084	T	C	0.092	0.018	0.582	6836	2.63E-07	0.0039	26.55
Phosphatidylcholine (O-18:1_20:3) levels	rs188004353	15	68561088	G	C	-0.230	0.051	0.032	6836	6.36E-06	0.0030	20.40
Phosphatidylcholine (O-	rs649854	16	15036737	G	A	-	0.01	0.33	683	1.93E-	0.005	36.13

18:1_20:3) levels	0					0.109	8	4	6	09	3	
Phosphatidylcholine (O-18:1_20:3) levels	rs12444786	16	82643141	G	A	0.108	0.023	0.821	6836	3.29E-06	0.0032	21.67
Phosphatidylcholine (O-18:1_20:3) levels	rs62131325	19	770714	T	C	-0.102	0.022	0.209	6836	5.29E-06	0.0030	20.75
Phosphatidylcholine (O-18:1_20:3) levels	rs71351740	19	33752643	G	A	-0.085	0.019	0.297	6836	6.11E-06	0.0030	20.48
Phosphatidylcholine (O-18:1_20:3) levels	rs7412	19	44908822	T	C	0.205	0.038	0.053	6836	9.45E-08	0.0042	28.54
Phosphatidylcholine (O-18:1_20:3) levels	rs111260004	20	35695325	C	G	-0.256	0.056	0.026	6836	4.57E-06	0.0031	21.03
Phosphatidylcholine (O-18:2_20:4) levels	rs12046045	1	47591044	A	G	-0.156	0.032	0.089	6127	1.12E-06	0.0039	23.75
Phosphatidylcholine (O-18:2_20:4) levels	rs1936090	1	164952845	A	G	-0.082	0.018	0.534	6127	6.91E-06	0.0033	20.24
Phosphatidylcholine (O-18:2_20:4) levels	rs3820438	1	201955621	C	G	0.088	0.020	0.291	6127	9.13E-06	0.0032	19.71
Phosphatidylcholine (O-18:2_20:4) levels	rs13401553	2	123610457	C	T	0.087	0.018	0.428	6127	2.18E-06	0.0037	22.46
Phosphatidylcholine (O-18:2_20:4) levels	rs6722606	2	191752537	C	A	-0.452	0.096	0.009	6127	2.52E-06	0.0036	22.18
Phosphatidylcholine (O-18:2_20:4) levels	rs7660654	4	179908526	T	A	-0.092	0.021	0.751	6127	9.70E-06	0.0032	19.59
Phosphatidylcholine (O-	rs112718	7	41177033	C	T	0.51	0.11	0.00	612	5.32E-	0.003	20.75

18:2_20:4) levels	171					7	3	7	7	06	4	
Phosphatidylcholine (O-18:2_20:4) levels	rs2189991	7	82556403	A	G	0.087	0.018	0.587	6127	1.53E-06	0.0038	23.14
Phosphatidylcholine (O-18:2_20:4) levels	rs6467318	7	130802167	G	A	-0.088	0.019	0.574	6127	2.82E-06	0.0036	21.97
Phosphatidylcholine (O-18:2_20:4) levels	rs73691232	8	76925244	A	C	-0.154	0.034	0.075	6127	7.59E-06	0.0033	20.07
Phosphatidylcholine (O-18:2_20:4) levels	rs76375697	9	32337256	G	T	0.340	0.071	0.018	6127	1.77E-06	0.0037	22.87
Phosphatidylcholine (O-18:2_20:4) levels	rs174537	11	61785208	T	G	-0.176	0.018	0.408	6127	1.97E-21	0.0146	91.01
Phosphatidylcholine (O-18:2_20:4) levels	rs7118021	11	89058340	A	G	-0.117	0.025	0.159	6127	1.95E-06	0.0037	22.67
Phosphatidylcholine (O-18:2_20:4) levels	rs113023745	13	66979217	A	G	-0.254	0.047	0.037	6127	9.21E-08	0.0046	28.59
Phosphatidylcholine (O-18:2_20:4) levels	rs1980615	14	67491655	C	A	-0.103	0.018	0.455	6127	1.12E-08	0.0053	32.71
Phosphatidylcholine (O-18:2_20:4) levels	rs17778967	15	43258624	A	G	0.250	0.053	0.032	6127	2.46E-06	0.0036	22.23
Phosphatidylcholine (O-18:2_20:4) levels	rs7203057	16	57439704	G	T	-0.200	0.044	0.041	6127	6.77E-06	0.0033	20.28
Phosphatidylcholine (O-18:2_20:4) levels	rs16975847	16	75555692	A	G	-0.124	0.026	0.134	6127	2.76E-06	0.0036	22.01
Phosphatidylcholine (O-	rs145525	17	13263077	T	C	0.17	0.03	0.06	612	5.59E-	0.003	20.65

18:2_20:4) levels	514					7	9	1	7	06	4	
Phosphatidylcholine (O-18:2_20:4) levels	rs141198865	17	17676043	T	C	-0.349	0.076	0.018	6127	4.02E-06	0.0035	21.29
Phosphatidylcholine (O-18:2_20:4) levels	rs117215929	18	27258198	C	T	-0.229	0.047	0.042	6127	1.00E-06	0.0039	23.96
Phosphatidylethanolamine (18:0_18:2)	rs1260326	2	27508073	C	T	-0.122	0.017	0.651	7128	2.57E-12	0.0068	49.14
Phosphatidylethanolamine (18:0_18:2)	rs4296402	2	174696563	A	G	0.106	0.023	0.160	7128	4.49E-06	0.0029	21.07
Phosphatidylethanolamine (18:0_18:2)	rs62291572	4	4996200	T	C	0.100	0.021	0.214	7128	1.96E-06	0.0032	22.67
Phosphatidylethanolamine (18:0_18:2)	rs13110318	4	38137235	A	G	-0.121	0.026	0.114	7128	3.74E-06	0.0030	21.42
Phosphatidylethanolamine (18:0_18:2)	rs72679383	4	100929253	T	G	0.286	0.059	0.022	7128	1.04E-06	0.0033	23.88
Phosphatidylethanolamine (18:0_18:2)	rs149431055	4	115874677	T	C	-0.243	0.055	0.025	7128	9.10E-06	0.0028	19.71
Phosphatidylethanolamine (18:0_18:2)	rs10069895	5	86297284	C	T	0.097	0.020	0.764	7128	7.18E-07	0.0034	24.60
Phosphatidylethanolamine (18:0_18:2)	rs141883259	5	120061007	G	A	0.373	0.083	0.010	7128	6.59E-06	0.0028	20.33
Phosphatidylethanolamine (18:0_18:2)	rs805292	6	31722232	A	G	0.085	0.017	0.378	7128	1.10E-06	0.0033	23.78
Phosphatidylethanolamine (18:0_18:2)	rs419482	6	94336592	T	A	-0.078	0.017	0.408	7128	4.12E-06	0.0030	21.23
Phosphatidylethanolamine	rs624342	6	154681357	A	G	0.10	0.02	0.16	712	2.35E-	0.003	22.31

(18:0_18:2)	28					9	3	0	8	06	1	
Phosphatidylethanolamine (18:0_18:2)	rs10276778	7	146994417	C	T	-0.182	0.040	0.044	7128	6.58E-06	0.0028	20.34
Phosphatidylethanolamine (18:0_18:2)	rs2946493	8	12966162	C	T	0.105	0.021	0.206	7128	4.35E-07	0.0036	25.57
Phosphatidylethanolamine (18:0_18:2)	rs10761714	10	63110944	A	G	-0.104	0.023	0.845	7128	6.47E-06	0.0029	20.37
Phosphatidylethanolamine (18:0_18:2)	rs174527	11	61770929	C	G	0.102	0.017	0.444	7128	2.73E-09	0.0049	35.45
Phosphatidylethanolamine (18:0_18:2)	rs174567	11	61825533	G	A	0.343	0.017	0.409	7128	1.25E-92	0.0568	429.03
Phosphatidylethanolamine (18:0_18:2)	rs117900629	11	62205328	A	G	-0.213	0.034	0.062	7128	6.25E-10	0.0054	38.34
Phosphatidylethanolamine (18:0_18:2)	rs964184	11	116778201	C	G	-0.154	0.023	0.849	7128	2.75E-11	0.0062	44.47
Phosphatidylethanolamine (18:0_18:2)	rs35828755	12	17547405	C	T	-0.092	0.021	0.195	7128	9.29E-06	0.0028	19.67
Phosphatidylethanolamine (18:0_18:2)	rs34733845	12	60861641	T	C	0.165	0.036	0.059	7128	3.86E-06	0.0030	21.36
Phosphatidylethanolamine (18:0_18:2)	rs138283783	15	58244328	C	T	0.352	0.054	0.027	7128	5.56E-11	0.0060	43.09
Phosphatidylethanolamine (18:0_18:2)	rs10468017	15	58386313	T	C	0.298	0.017	0.338	7128	9.03E-66	0.0403	299.56
Phosphatidylethanolamine (18:0_18:2)	rs633695	15	58433640	G	A	0.255	0.018	0.314	7128	5.56E-45	0.0274	200.79
Phosphatidylethanolamine (18:0_18:2)	rs117376818	15	58506762	A	G	0.630	0.070	0.016	7128	4.16E-19	0.0111	80.21

Phosphatidylethanolamine (18:0_18:2)	rs73424597	15	58607410	C	T	0.355	0.045	0.035	7128	5.88E-15	0.0085	61.18
Phosphatidylethanolamine (18:0_18:2)	rs112129861	15	59047450	C	G	0.156	0.034	0.065	7128	3.91E-06	0.0030	21.33
Phosphatidylethanolamine (18:0_18:2)	rs111543310	15	59239619	C	T	0.280	0.060	0.022	7128	3.55E-06	0.0030	21.52
Phosphatidylethanolamine (18:0_18:2)	rs12928099	16	15056648	A	C	-0.113	0.019	0.274	7128	1.50E-09	0.0051	36.62
Phosphatidylethanolamine (18:0_18:2)	rs449480	16	84499954	C	G	0.076	0.017	0.541	7128	5.89E-06	0.0029	20.55
Phosphatidylethanolamine (18:0_18:2)	rs7226158	17	79394643	C	T	-0.081	0.018	0.684	7128	6.06E-06	0.0029	20.49
Phosphatidylethanolamine (18:0_18:2)	rs2840005	19	55803533	C	T	-0.111	0.025	0.133	7128	8.44E-06	0.0028	19.86
Phosphatidylethanolamine (O-18:1_20:4)	rs1203148	1	35503298	G	A	0.091	0.020	0.240	7172	7.34E-06	0.0028	20.12
Phosphatidylethanolamine (O-18:1_20:4)	rs114325590	1	107442367	T	C	-0.379	0.085	0.011	7172	9.41E-06	0.0027	19.65
Phosphatidylethanolamine (O-18:1_20:4)	rs3820438	1	201955621	C	G	0.082	0.018	0.291	7172	8.42E-06	0.0028	19.86
Phosphatidylethanolamine (O-18:1_20:4)	rs72635955	1	244982806	T	C	0.126	0.025	0.125	7172	5.16E-07	0.0035	25.24
Phosphatidylethanolamine (O-18:1_20:4)	rs71420341	2	59638953	G	A	-0.146	0.030	0.089	7172	7.54E-07	0.0034	24.51
Phosphatidylethanolamine (O-18:1_20:4)	rs1513795	2	123647385	C	T	0.080	0.017	0.429	7172	2.47E-06	0.0031	22.21
Phosphatidylethanolamine (O-	rs117252	4	52717255	C	T	0.25	0.05	0.02	717	6.50E-	0.002	20.36

18:1_20:4)	98					6	7	2	2	06	8	
Phosphatidylethanolamine (O-18:1_20:4)	rs10520189	4	170720084	A	G	0.122	0.027	0.114	7172	7.55E-06	0.0028	20.07
Phosphatidylethanolamine (O-18:1_20:4)	rs10234951	7	94419222	G	T	-0.149	0.031	0.080	7172	1.69E-06	0.0032	22.95
Phosphatidylethanolamine (O-18:1_20:4)	rs2705007	7	106360522	G	C	-0.361	0.079	0.987	7172	5.12E-06	0.0029	20.82
Phosphatidylethanolamine (O-18:1_20:4)	rs6601694	8	8390548	A	G	0.088	0.018	0.334	7172	8.85E-07	0.0034	24.20
Phosphatidylethanolamine (O-18:1_20:4)	rs311416	8	54198249	T	C	-0.079	0.018	0.649	7172	6.58E-06	0.0028	20.34
Phosphatidylethanolamine (O-18:1_20:4)	rs13269712	8	69966863	A	G	0.105	0.023	0.160	7172	6.82E-06	0.0028	20.27
Phosphatidylethanolamine (O-18:1_20:4)	rs10756627	9	14928596	C	T	0.075	0.017	0.551	7172	8.04E-06	0.0028	19.95
Phosphatidylethanolamine (O-18:1_20:4)	rs117002537	9	130943915	T	C	0.291	0.065	0.018	7172	8.52E-06	0.0028	19.84
Phosphatidylethanolamine (O-18:1_20:4)	rs174527	11	61770929	C	G	-0.079	0.017	0.444	7172	3.97E-06	0.0030	21.31
Phosphatidylethanolamine (O-18:1_20:4)	rs174536	11	61784455	C	A	-0.251	0.017	0.408	7172	1.09E-49	0.0302	222.97
Phosphatidylethanolamine (O-18:1_20:4)	rs2277289	11	118311945	C	T	0.091	0.018	0.326	7172	3.31E-07	0.0036	26.10
Phosphatidylethanolamine (O-18:1_20:4)	rs11062287	12	2630538	A	G	0.255	0.050	0.034	7172	4.45E-07	0.0035	25.53
Phosphatidylethanolamine (O-18:1_20:4)	rs113913626	12	76255614	T	C	-0.22	0.048	0.032	7172	3.27E-06	0.0030	21.68

						5						
Phosphatidylethanolamine (O-18:1_20:4)	rs17703316	13	75596558	T	G	0.179	0.039	0.050	7172	4.25E-06	0.0029	21.18
Phosphatidylethanolamine (O-18:1_20:4)	rs4525452	15	96625932	A	G	0.077	0.017	0.393	7172	7.24E-06	0.0028	20.15
Phosphatidylethanolamine (O-18:1_20:4)	rs117565387	16	5924670	T	C	0.175	0.038	0.053	7172	3.51E-06	0.0030	21.54
Phosphatidylethanolamine (O-18:1_20:4)	rs35427954	16	76463097	C	T	-0.140	0.030	0.087	7172	2.30E-06	0.0031	22.36
Phosphatidylethanolamine (O-18:1_20:4)	rs1456608	18	44272134	G	T	-0.087	0.019	0.755	7172	5.93E-06	0.0029	20.53
Phosphatidylethanolamine (O-18:1_20:4)	rs141622900	19	44923535	A	G	0.250	0.050	0.030	7172	6.52E-07	0.0034	24.79
Phosphatidylethanolamine (O-18:1_20:4)	rs204468	19	44987378	C	T	-0.095	0.017	0.595	7172	2.35E-08	0.0043	31.24
Phosphatidylethanolamine (O-18:1_20:4)	rs41376249	22	36714714	A	G	-0.112	0.024	0.146	7172	2.08E-06	0.0031	22.55
Phosphatidylethanolamine (O-18:2_18:1)	rs17368528	1	9264154	T	C	0.161	0.031	0.084	6717	2.04E-07	0.0040	27.04
Phosphatidylethanolamine (O-18:2_18:1)	rs148727465	1	167181238	A	G	0.209	0.046	0.040	6717	6.67E-06	0.0030	20.31
Phosphatidylethanolamine (O-18:2_18:1)	rs112243303	1	224192928	T	C	0.138	0.028	0.112	6717	5.86E-07	0.0037	24.99
Phosphatidylethanolamine (O-18:2_18:1)	rs116075306	1	241342846	G	C	-0.371	0.084	0.011	6717	9.30E-06	0.0029	19.67
Phosphatidylethanolamine (O-18:2_18:1)	rs79878108	1	245122916	T	C	-0.26	0.052	0.029	6717	5.08E-07	0.0037	25.27

						1						
Phosphatidylethanolamine (O-18:2_18:1)	rs35253870	2	12232463	G	A	0.118	0.024	0.151	6717	1.39E-06	0.0035	23.32
Phosphatidylethanolamine (O-18:2_18:1)	rs112779958	2	14315428	C	T	-0.234	0.052	0.030	6717	6.07E-06	0.0030	20.49
Phosphatidylethanolamine (O-18:2_18:1)	rs192913427	2	63521077	A	G	0.718	0.152	0.003	6717	2.42E-06	0.0033	22.26
Phosphatidylethanolamine (O-18:2_18:1)	rs6749797	2	120398486	A	G	-0.098	0.020	0.229	6717	1.74E-06	0.0034	22.90
Phosphatidylethanolamine (O-18:2_18:1)	rs4674246	2	218075695	T	C	0.102	0.023	0.186	6717	7.63E-06	0.0030	20.05
Phosphatidylethanolamine (O-18:2_18:1)	rs12496575	3	236587	G	A	-0.090	0.019	0.693	6717	1.65E-06	0.0034	23.00
Phosphatidylethanolamine (O-18:2_18:1)	rs2271489	3	39235168	G	C	-0.172	0.032	0.081	6717	6.67E-08	0.0043	29.21
Phosphatidylethanolamine (O-18:2_18:1)	rs116140603	5	180049450	C	T	0.576	0.130	0.004	6717	9.21E-06	0.0029	19.69
Phosphatidylethanolamine (O-18:2_18:1)	rs3752651	7	55161850	C	T	0.115	0.025	0.136	6717	5.07E-06	0.0031	20.84
Phosphatidylethanolamine (O-18:2_18:1)	rs13257713	8	112417407	T	C	-0.119	0.025	0.139	6717	2.06E-06	0.0033	22.57
Phosphatidylethanolamine (O-18:2_18:1)	rs117049823	9	2495519	G	A	0.489	0.110	0.006	6717	8.55E-06	0.0029	19.83
Phosphatidylethanolamine (O-18:2_18:1)	rs140484398	9	102668496	A	G	0.519	0.106	0.007	6717	1.02E-06	0.0036	23.93
Phosphatidylethanolamine (O-18:2_18:1)	rs150046733	11	732354	C	T	-0.18	0.041	0.049	6717	8.64E-06	0.0029	19.81

						1						
Phosphatidylethanolamine (O-18:2_18:1)	rs11174276	12	62005843	A	G	0.083	0.019	0.318	6717	7.22E-06	0.0030	20.16
Phosphatidylethanolamine (O-18:2_18:1)	rs1800588	15	58431476	T	C	0.095	0.020	0.258	6717	1.94E-06	0.0034	22.68
Phosphatidylethanolamine (O-18:2_18:1)	rs28606541	19	6934007	T	C	-0.207	0.043	0.043	6717	1.71E-06	0.0034	22.93
Phosphatidylethanolamine (O-18:2_18:1)	rs8108277	19	44702607	C	T	0.106	0.022	0.189	6717	1.13E-06	0.0035	23.73
Phosphatidylinositol (18:1_20:4) levels	rs1168127	1	62670407	A	C	0.105	0.021	0.737	5882	5.26E-07	0.0043	25.21
Phosphatidylinositol (18:1_20:4) levels	rs117960039	1	97483748	G	A	0.250	0.056	0.029	5882	7.25E-06	0.0034	20.15
Phosphatidylinositol (18:1_20:4) levels	rs4920209	1	234028770	A	G	0.088	0.018	0.475	5882	1.63E-06	0.0039	23.03
Phosphatidylinositol (18:1_20:4) levels	rs12467458	2	213973289	G	T	0.126	0.028	0.126	5882	6.52E-06	0.0035	20.36
Phosphatidylinositol (18:1_20:4) levels	rs140854080	4	87407252	A	G	0.370	0.078	0.014	5882	2.47E-06	0.0038	22.23
Phosphatidylinositol (18:1_20:4) levels	rs2379249	5	17631738	T	G	-0.162	0.036	0.927	5882	8.22E-06	0.0034	19.91
Phosphatidylinositol (18:1_20:4) levels	rs114918906	5	174699125	T	C	0.193	0.044	0.049	5882	9.38E-06	0.0033	19.66
Phosphatidylinositol (18:1_20:4) levels	rs55976852	6	94237667	C	A	-0.099	0.021	0.274	5882	1.92E-06	0.0038	22.71
Phosphatidylinositol (18:1_20:4) levels	rs2819083	6	156379682	A	G	-0.092	0.018	0.486	5882	4.92E-07	0.0043	25.35
Phosphatidylinositol (18:1_20:4)	rs360613	8	100795002	T	C	0.19	0.04	0.05	588	2.94E-	0.003	21.89

levels	40					4	1	2	2	06	7	
Phosphatidylinositol (18:1_20:4) levels	rs12554791	9	31860328	G	T	0.136	0.030	0.110	5882	6.69E-06	0.0034	20.31
Phosphatidylinositol (18:1_20:4) levels	rs13291383	9	133897690	C	T	0.096	0.021	0.276	5882	7.48E-06	0.0034	20.09
Phosphatidylinositol (18:1_20:4) levels	rs74559802	10	6185479	A	G	-0.432	0.097	0.011	5882	9.56E-06	0.0033	19.62
Phosphatidylinositol (18:1_20:4) levels	rs670975	11	76222144	T	A	0.173	0.038	0.067	5882	4.20E-06	0.0036	21.20
Phosphatidylinositol (18:1_20:4) levels	rs3741298	11	116786845	T	C	-0.127	0.022	0.765	5882	4.35E-09	0.0058	34.55
Phosphatidylinositol (18:1_20:4) levels	rs61915733	12	18171023	A	G	0.152	0.032	0.090	5882	2.09E-06	0.0038	22.55
Phosphatidylinositol (18:1_20:4) levels	rs111645166	13	31552770	A	G	-0.465	0.105	0.009	5882	9.76E-06	0.0033	19.58
Phosphatidylinositol (18:1_20:4) levels	rs74572381	14	35052659	A	G	0.173	0.039	0.062	5882	8.84E-06	0.0034	19.77
Phosphatidylinositol (18:1_20:4) levels	rs117598891	14	95031978	C	T	-0.482	0.105	0.008	5882	4.91E-06	0.0035	20.91
Phosphatidylinositol (18:1_20:4) levels	rs10775270	16	10136125	C	A	-0.096	0.022	0.753	5882	9.48E-06	0.0033	19.64
Phosphatidylinositol (18:1_20:4) levels	rs117210444	16	73859072	G	A	-0.287	0.050	0.034	5882	9.19E-09	0.0056	33.09
Phosphatidylinositol (18:1_20:4) levels	rs12938892	17	10103775	G	A	-0.087	0.019	0.437	5882	6.54E-06	0.0034	20.35

Phosphatidylinositol (18:1_20:4) levels	rs957142	18	49619347	A	G	0.152	0.032	0.094	5882	2.24E-06	0.0038	22.42
Phosphatidylinositol (18:1_20:4) levels	rs75166839	18	73825578	G	A	0.301	0.065	0.022	5882	3.70E-06	0.0036	21.44
Phosphatidylinositol (18:1_20:4) levels	rs146290629	19	2402297	T	C	-0.302	0.068	0.021	5882	8.49E-06	0.0034	19.85
Phosphatidylinositol (18:1_20:4) levels	rs599859	19	52584863	C	A	-0.089	0.019	0.394	5882	4.86E-06	0.0035	20.92
Phosphatidylinositol (18:1_20:4) levels	rs4822502	22	24485813	T	C	0.084	0.019	0.545	5882	5.54E-06	0.0035	20.67
Sphingomyelin (d34:0) levels	rs11591147	1	55039974	T	G	-0.256	0.053	0.033	6207	1.33E-06	0.0038	23.42
Sphingomyelin (d34:0) levels	rs6683774	1	198625465	T	C	0.178	0.037	0.934	6207	1.17E-06	0.0038	23.66
Sphingomyelin (d34:0) levels	rs17497891	1	222184468	G	A	0.119	0.025	0.150	6207	2.51E-06	0.0036	22.19
Sphingomyelin (d34:0) levels	rs934198	2	21055901	T	G	0.098	0.020	0.297	6207	8.40E-07	0.0039	24.30
Sphingomyelin (d34:0) levels	rs114372832	2	75724208	C	G	-0.300	0.067	0.020	6207	7.69E-06	0.0032	20.04
Sphingomyelin (d34:0) levels	rs55811539	3	113313552	C	T	-0.399	0.084	0.012	6207	1.83E-06	0.0037	22.80
Sphingomyelin (d34:0) levels	rs112585713	4	72876701	A	G	0.262	0.051	0.032	6207	2.96E-07	0.0042	26.32
Sphingomyelin (d34:0) levels	rs16850360	4	74006728	G	A	0.227	0.050	0.033	6207	6.51E-06	0.0033	20.36
Sphingomyelin (d34:0) levels	rs140560	7	11194365	G	A	-	0.08	0.01	620	9.31E-	0.003	19.67

	087					0.377	5	2	7	06	2	
Sphingomyelin (d34:0) levels	rs7814780	8	122227701	T	G	-0.087	0.019	0.328	6207	6.75E-06	0.0033	20.29
Sphingomyelin (d34:0) levels	rs35275531	8	142178314	A	C	-0.269	0.059	0.026	6207	5.38E-06	0.0033	20.73
Sphingomyelin (d34:0) levels	rs1324162	9	80858018	G	T	0.090	0.020	0.271	6207	7.81E-06	0.0032	20.01
Sphingomyelin (d34:0) levels	rs2808569	9	98537277	C	A	-0.098	0.021	0.242	6207	3.51E-06	0.0035	21.55
Sphingomyelin (d34:0) levels	rs80130206	10	106229406	T	C	-0.182	0.039	0.055	6207	2.99E-06	0.0035	21.85
Sphingomyelin (d34:0) levels	rs113312978	11	59731240	A	G	-0.645	0.146	0.004	6207	9.79E-06	0.0031	19.58
Sphingomyelin (d34:0) levels	rs174535	11	61783884	C	T	-0.098	0.018	0.409	6207	1.14E-07	0.0045	28.18
Sphingomyelin (d34:0) levels	rs12880341	14	63769473	C	T	-0.327	0.027	0.125	6207	3.94E-33	0.0229	145.43
Sphingomyelin (d34:0) levels	rs11631073	15	57322347	T	C	-0.091	0.019	0.618	6207	1.11E-06	0.0038	23.77
Sphingomyelin (d34:0) levels	rs55985238	17	3769209	A	T	-0.560	0.101	0.009	6207	2.72E-08	0.0050	30.96
Sphingomyelin (d34:0) levels	rs756796	17	4764677	A	C	-	0.06	0.02	620	3.31E-	0.019	122.4

	63					0.689	2	6	7	28	4	6
Sphingomyelin (d34:0) levels	rs3026120	17	5384095	A	G	-0.207	0.034	0.075	6207	1.44E-09	0.0059	36.71
Sphingomyelin (d34:0) levels	rs8071514	17	80990283	A	C	0.085	0.018	0.569	6207	2.80E-06	0.0035	21.98
Sphingomyelin (d34:0) levels	rs73015021	19	11082239	G	A	-0.153	0.032	0.092	6207	1.32E-06	0.0038	23.42
Sphingomyelin (d34:0) levels	rs61751862	19	48629610	C	G	-0.241	0.053	0.031	6207	5.67E-06	0.0033	20.63
Sphingomyelin (d34:0) levels	rs1073042	20	11012603	G	A	-0.082	0.018	0.397	6207	6.87E-06	0.0033	20.25
Sphingomyelin (d34:0) levels	rs595587	21	43371358	C	T	0.090	0.019	0.340	6207	2.56E-06	0.0036	22.16
Sphingomyelin (d34:0) levels	rs1989780	22	19694202	A	G	-0.301	0.068	0.020	6207	9.27E-06	0.0032	19.68
Sphingomyelin (d34:2) levels	rs56163454	1	44173695	G	C	0.267	0.059	0.020	7174	5.58E-06	0.0029	20.65
Sphingomyelin (d34:2) levels	rs11591147	1	55039974	T	G	-0.246	0.047	0.033	7174	1.27E-07	0.0039	27.97
Sphingomyelin (d34:2) levels	rs9835223	3	186868495	G	C	-0.095	0.021	0.801	7174	6.54E-06	0.0028	20.35
Sphingomyelin (d34:2) levels	rs2531191	4	10615596	T	C	0.371	0.083	0.011	7174	8.23E-06	0.0028	19.91
Sphingomyelin (d34:2) levels	rs749154	4	72899760	A	G	0.19	0.04	0.03	717	9.99E-	0.002	19.53

	47					6	4	8	4	06	7	
Sphingomyelin (d34:2) levels	rs116302332	4	74505174	T	C	0.298	0.051	0.028	7174	4.74E-09	0.0048	34.36
Sphingomyelin (d34:2) levels	rs10471676	5	66284563	G	C	-0.092	0.020	0.226	7174	6.75E-06	0.0028	20.29
Sphingomyelin (d34:2) levels	rs146136464	5	147384423	T	C	0.793	0.176	0.003	7174	6.79E-06	0.0028	20.27
Sphingomyelin (d34:2) levels	rs62412280	6	67724705	T	A	-0.656	0.140	0.004	7174	2.97E-06	0.0030	21.86
Sphingomyelin (d34:2) levels	rs7787163	7	89538067	T	C	0.477	0.103	0.007	7174	3.33E-06	0.0030	21.64
Sphingomyelin (d34:2) levels	rs1404846	7	110130812	C	T	0.696	0.154	0.003	7174	6.24E-06	0.0028	20.43
Sphingomyelin (d34:2) levels	rs7012814	8	9315848	A	G	0.087	0.017	0.512	7174	2.67E-07	0.0037	26.51
Sphingomyelin (d34:2) levels	rs10975312	9	5837780	A	G	0.237	0.049	0.031	7174	1.18E-06	0.0033	23.65
Sphingomyelin (d34:2) levels	rs10867632	9	80862163	G	C	0.092	0.021	0.207	7174	8.84E-06	0.0027	19.77
Sphingomyelin (d34:2) levels	rs113127305	10	70837164	A	G	-0.217	0.038	0.050	7174	1.46E-08	0.0045	32.17
Sphingomyelin (d34:2) levels	rs174561	11	61815236	C	T	-0.170	0.017	0.381	7174	7.26E-23	0.0134	97.56
Sphingomyelin (d34:2) levels	rs3912622	11	103250744	C	A	-0.093	0.021	0.200	7174	9.05E-06	0.0027	19.72
Sphingomyelin (d34:2) levels	rs117009181	12	124846478	C	T	0.110	0.023	0.150	7174	2.03E-06	0.0031	22.59

Sphingomyelin (d34:2) levels	rs111507730	13	33753876	C	T	0.171	0.037	0.054	7174	3.33E-06	0.0030	21.64
Sphingomyelin (d34:2) levels	rs17276940	14	20017956	T	C	-0.214	0.046	0.034	7174	3.48E-06	0.0030	21.56
Sphingomyelin (d34:2) levels	rs1033030	14	99309314	A	G	-0.105	0.022	0.176	7174	2.16E-06	0.0031	22.48
Sphingomyelin (d34:2) levels	rs1532085	15	58391167	G	A	-0.109	0.017	0.573	7174	7.57E-11	0.0059	42.48
Sphingomyelin (d34:2) levels	rs939971	15	80029927	A	C	-0.096	0.020	0.211	7174	2.43E-06	0.0031	22.25
Sphingomyelin (d34:2) levels	rs12952341	17	4482208	T	C	0.085	0.019	0.326	7174	4.84E-06	0.0029	20.93
Sphingomyelin (d34:2) levels	rs113722226	19	11078596	C	T	-0.186	0.028	0.094	7174	3.70E-11	0.0061	43.89
Sphingomyelin (d34:2) levels	rs7247433	19	19684751	A	C	-0.078	0.017	0.394	7174	7.82E-06	0.0028	20.00
Sphingomyelin (d34:2) levels	rs61751862	19	48629610	C	G	0.253	0.048	0.031	7174	1.39E-07	0.0039	27.79
Sphingomyelin (d34:2) levels	rs1800961	20	44413724	T	C	-0.168	0.037	0.052	7174	5.97E-06	0.0029	20.52
Sphingomyelin (d36:1) levels	rs11591147	1	55039974	T	G	-0.259	0.047	0.033	7174	2.96E-08	0.0043	30.79
Sphingomyelin (d36:1) levels	rs1452773	2	50050811	G	T	-0.11	0.024	0.138	7174	1.10E-06	0.0033	23.78

						7						
Sphingomyelin (d36:1) levels	rs12464353	2	60982893	A	G	0.229	0.048	0.033	7174	1.74E-06	0.0032	22.89
Sphingomyelin (d36:1) levels	rs886813	2	241457278	T	G	0.081	0.017	0.468	7174	1.61E-06	0.0032	23.04
Sphingomyelin (d36:1) levels	rs73073606	3	194176577	C	A	-0.090	0.020	0.241	7174	8.25E-06	0.0028	19.90
Sphingomyelin (d36:1) levels	rs112585713	4	72876701	A	G	0.282	0.048	0.032	7174	4.41E-09	0.0048	34.50
Sphingomyelin (d36:1) levels	rs79146711	4	73424911	T	C	0.234	0.041	0.045	7174	1.02E-08	0.0046	32.87
Sphingomyelin (d36:1) levels	rs182695896	4	73947510	C	A	0.339	0.053	0.025	7174	1.58E-10	0.0057	41.03
Sphingomyelin (d36:1) levels	rs6829637	4	110405753	G	A	-0.079	0.017	0.416	7174	4.77E-06	0.0029	20.95
Sphingomyelin (d36:1) levels	rs114839471	5	90249218	G	A	-0.445	0.095	0.008	7174	2.59E-06	0.0031	22.13
Sphingomyelin (d36:1) levels	rs884532	5	174239481	T	C	-0.075	0.017	0.510	7174	6.95E-06	0.0028	20.23
Sphingomyelin (d36:1) levels	rs4144237	6	2365034	G	A	-0.113	0.025	0.126	7174	8.50E-06	0.0028	19.84
Sphingomyelin (d36:1) levels	rs17664981	6	13187371	C	T	0.167	0.037	0.057	7174	5.34E-06	0.0029	20.74
Sphingomyelin (d36:1) levels	rs374071816	6	160682773	A	T	0.161	0.036	0.057	7174	9.69E-06	0.0027	19.59
Sphingomyelin (d36:1) levels	rs12675806	8	4789612	A	G	0.126	0.026	0.112	7174	1.90E-06	0.0032	22.72

Sphingomyelin (d36:1) levels	rs6558122	8	29518250	C	T	-0.140	0.028	0.101	7174	4.58E-07	0.0035	25.47
Sphingomyelin (d36:1) levels	rs11779209	8	71073538	G	T	0.114	0.026	0.126	7174	9.39E-06	0.0027	19.65
Sphingomyelin (d36:1) levels	rs581080	9	15305380	C	G	0.114	0.024	0.856	7174	1.47E-06	0.0032	23.22
Sphingomyelin (d36:1) levels	rs117442205	11	4966841	A	G	0.609	0.138	0.004	7174	9.71E-06	0.0027	19.59
Sphingomyelin (d36:1) levels	rs10838826	11	48198260	T	G	0.092	0.020	0.783	7174	5.13E-06	0.0029	20.81
Sphingomyelin (d36:1) levels	rs4246215	11	61796827	T	G	-0.075	0.017	0.436	7174	8.88E-06	0.0027	19.76
Sphingomyelin (d36:1) levels	rs3742023	12	109256177	T	C	-0.077	0.017	0.423	7174	5.63E-06	0.0029	20.63
Sphingomyelin (d36:1) levels	rs1169306	12	121000508	T	C	-0.085	0.017	0.360	7174	9.27E-07	0.0033	24.11
Sphingomyelin (d36:1) levels	rs4981443	14	22831454	A	G	-0.077	0.017	0.360	7174	9.63E-06	0.0027	19.60
Sphingomyelin (d36:1) levels	rs7157785	14	63768838	T	G	0.136	0.025	0.125	7174	6.56E-08	0.0041	29.24
Sphingomyelin (d36:1) levels	rs138524673	15	68380799	A	G	0.193	0.042	0.045	7174	5.75E-06	0.0029	20.59
Sphingomyelin (d36:1) levels	rs186039163	17	3547599	G	T	-0.416	0.084	0.010	7174	7.74E-07	0.0034	24.46
Sphingomyelin (d36:1) levels	rs75679663	17	4764677	A	C	-0.32	0.054	0.026	7174	1.42E-09	0.0051	36.72

						5						
Sphingomyelin (d36:1) levels	rs117643293	17	5065669	T	C	-0.171	0.034	0.062	7174	5.50E-07	0.0035	25.12
Sphingomyelin (d36:1) levels	rs72898481	17	77755768	T	C	0.286	0.064	0.019	7174	6.72E-06	0.0028	20.30
Sphingomyelin (d36:1) levels	rs2336171	19	8209156	C	G	0.195	0.017	0.399	7174	2.94E-30	0.0180	131.82
Sphingomyelin (d36:1) levels	rs7248003	19	8236164	T	C	0.145	0.019	0.721	7174	2.69E-14	0.0080	58.17
Sphingomyelin (d36:1) levels	rs12462619	19	8607097	C	T	-0.108	0.019	0.261	7174	2.32E-08	0.0043	31.26
Sphingomyelin (d36:1) levels	rs364585	20	12982070	G	A	-0.109	0.018	0.661	7174	5.55E-10	0.0053	38.56
Sphingomyelin (d36:1) levels	rs4809845	20	51460945	C	T	0.129	0.025	0.134	7174	2.41E-07	0.0037	26.71
Sphingomyelin (d36:1) levels	rs8139868	22	17172477	T	C	-0.088	0.019	0.257	7174	5.04E-06	0.0029	20.85
Sphingomyelin (d36:2) levels	rs1452773	2	50050811	G	T	-0.109	0.024	0.138	7173	5.98E-06	0.0029	20.52
Sphingomyelin (d36:2) levels	rs12464353	2	60982893	A	G	0.221	0.048	0.033	7173	4.04E-06	0.0030	21.27
Sphingomyelin (d36:2) levels	rs13005731	2	64114191	G	T	-0.504	0.113	0.006	7173	7.86E-06	0.0028	19.99
Sphingomyelin (d36:2) levels	rs78999781	2	203425314	C	T	-0.148	0.029	0.091	7173	3.95E-07	0.0036	25.76

Sphingomyelin (d36:2) levels	rs146465816	3	19323998	A	G	-0.361	0.081	0.011	7173	8.07E-06	0.0028	19.94
Sphingomyelin (d36:2) levels	rs6444176	3	186868271	T	C	0.175	0.038	0.052	7173	3.22E-06	0.0030	21.71
Sphingomyelin (d36:2) levels	rs77645768	4	72803111	A	G	0.243	0.054	0.026	7173	6.45E-06	0.0028	20.37
Sphingomyelin (d36:2) levels	rs182695896	4	73947510	C	A	0.303	0.053	0.025	7173	1.12E-08	0.0045	32.68
Sphingomyelin (d36:2) levels	rs313950	4	113100013	A	G	-0.076	0.017	0.396	7173	7.32E-06	0.0028	20.13
Sphingomyelin (d36:2) levels	rs146253188	5	35225964	A	G	-0.288	0.064	0.017	7173	8.07E-06	0.0028	19.94
Sphingomyelin (d36:2) levels	rs7700432	5	130859001	A	G	-0.079	0.017	0.354	7173	4.55E-06	0.0029	21.04
Sphingomyelin (d36:2) levels	rs12207488	6	10952103	A	G	-0.096	0.022	0.182	7173	8.21E-06	0.0028	19.91
Sphingomyelin (d36:2) levels	rs117546336	6	98098087	G	T	-0.190	0.040	0.047	7173	2.00E-06	0.0031	22.62
Sphingomyelin (d36:2) levels	rs581080	9	15305380	C	G	0.108	0.024	0.856	7173	5.20E-06	0.0029	20.79
Sphingomyelin (d36:2) levels	rs7920200	10	70899547	A	G	-0.188	0.042	0.042	7173	8.75E-06	0.0028	19.79
Sphingomyelin (d36:2) levels	rs174544	11	61800281	A	C	-0.178	0.017	0.380	7173	5.30E-25	0.0148	107.43

Sphingomyelin (d36:2) levels	rs9738226	12	120985856	G	A	0.101	0.017	0.579	7173	2.37E-09	0.0050	35.72
Sphingomyelin (d36:2) levels	rs17276940	14	20017956	T	C	-0.219	0.046	0.034	7173	2.12E-06	0.0031	22.51
Sphingomyelin (d36:2) levels	rs72879665	18	13203131	A	G	0.382	0.083	0.011	7173	4.73E-06	0.0029	20.97
Sphingomyelin (d36:2) levels	rs7253584	19	8222034	C	T	0.181	0.018	0.675	7173	2.48E-24	0.0143	104.32
Sphingomyelin (d36:2) levels	rs7247304	19	8602475	T	C	-0.103	0.019	0.267	7173	9.07E-08	0.0040	28.61
Sphingomyelin (d36:2) levels	rs68137603	20	37965215	T	C	0.153	0.035	0.062	7173	9.80E-06	0.0027	19.57
Sphingomyelin (d36:2) levels	rs148860058	22	48612019	A	G	0.401	0.087	0.011	7173	4.37E-06	0.0029	21.13
Sphingomyelin (d38:2) levels	rs11591147	1	55039974	T	G	-0.219	0.047	0.033	7150	2.84E-06	0.0031	21.95
Sphingomyelin (d38:2) levels	rs116411822	2	46494121	G	A	-0.420	0.094	0.008	7150	7.29E-06	0.0028	20.14
Sphingomyelin (d38:2) levels	rs10206976	2	210749914	T	G	0.091	0.019	0.268	7150	1.88E-06	0.0032	22.74
Sphingomyelin (d38:2) levels	rs3936109	3	184623650	G	A	-0.104	0.022	0.180	7150	2.72E-06	0.0031	22.03
Sphingomyelin (d38:2) levels	rs77645768	4	72803111	A	G	0.268	0.054	0.026	7150	6.16E-07	0.0035	24.89
Sphingomyelin (d38:2) levels	rs114483871	4	73124451	T	C	0.220	0.045	0.038	7150	9.38E-07	0.0034	24.08
Sphingomyelin (d38:2) levels	rs182695	4	73947510	C	A	0.32	0.05	0.02	715	8.57E-	0.005	37.72

	896					6	3	5	0	10	2	
Sphingomyelin (d38:2) levels	rs114717988	6	48788044	A	T	0.197	0.040	0.046	7150	6.37E-07	0.0035	24.83
Sphingomyelin (d38:2) levels	rs581080	9	15305380	C	G	0.115	0.024	0.856	7150	1.19E-06	0.0033	23.63
Sphingomyelin (d38:2) levels	rs672959	10	14418295	G	A	-0.459	0.100	0.008	7150	4.67E-06	0.0029	20.99
Sphingomyelin (d38:2) levels	rs139150589	10	70459848	T	C	0.521	0.100	0.008	7150	1.87E-07	0.0038	27.20
Sphingomyelin (d38:2) levels	rs72839716	10	122833455	A	G	-0.108	0.023	0.159	7150	2.53E-06	0.0031	22.17
Sphingomyelin (d38:2) levels	rs174556	11	61813163	T	C	-0.144	0.017	0.380	7150	6.46E-17	0.0097	70.17
Sphingomyelin (d38:2) levels	rs657315	11	69517944	T	C	0.082	0.018	0.284	7150	8.75E-06	0.0028	19.79
Sphingomyelin (d38:2) levels	rs75038600	12	12341682	C	G	0.726	0.160	0.003	7150	5.94E-06	0.0029	20.53
Sphingomyelin (d38:2) levels	rs1169306	12	121000508	T	C	-0.121	0.017	0.360	7150	4.14E-12	0.0067	48.21
Sphingomyelin (d38:2) levels	rs75098294	13	62988645	G	A	0.527	0.107	0.006	7150	9.06E-07	0.0034	24.15
Sphingomyelin (d38:2) levels	rs17276940	14	20017956	T	C	-0.245	0.046	0.034	7150	1.02E-07	0.0040	28.38
Sphingomyelin (d38:2) levels	rs113118892	15	59274186	T	C	0.383	0.080	0.012	7150	1.58E-06	0.0032	23.08
Sphingomyelin (d38:2) levels	rs146723536	16	12147559	A	G	-0.25	0.057	0.022	7150	8.40E-06	0.0028	19.87

						6						
Sphingomyelin (d38:2) levels	rs72794954	16	76342662	T	C	-0.490	0.105	0.007	7150	3.02E-06	0.0030	21.83
Sphingomyelin (d38:2) levels	rs142118024	18	67530560	G	A	-0.303	0.068	0.019	7150	8.02E-06	0.0028	19.95
Sphingomyelin (d38:2) levels	rs2336171	19	8209156	C	G	0.261	0.017	0.399	7150	5.70E-53	0.0323	238.50
Sphingomyelin (d38:2) levels	rs7248003	19	8236164	T	C	0.152	0.019	0.721	7150	2.09E-15	0.0088	63.24
Sphingomyelin (d38:2) levels	rs250507	19	8268177	C	T	-0.109	0.020	0.229	7150	7.49E-08	0.0040	28.98
Sphingomyelin (d38:2) levels	rs10405061	19	8341496	T	C	-0.095	0.018	0.309	7150	2.01E-07	0.0038	27.06
Sphingomyelin (d38:2) levels	rs12462619	19	8607097	C	T	-0.131	0.019	0.261	7150	1.44E-11	0.0064	45.76
Sphingomyelin (d38:2) levels	rs4804319	19	8628548	A	C	-0.087	0.018	0.691	7150	1.74E-06	0.0032	22.90
Sphingomyelin (d38:2) levels	rs61751862	19	48629610	C	G	0.230	0.048	0.031	7150	1.70E-06	0.0032	22.94
Sphingomyelin (d38:2) levels	rs142551539	21	29991071	T	C	0.545	0.122	0.005	7150	7.56E-06	0.0028	20.07
Sphingomyelin (d38:2) levels	rs148683988	22	48622225	G	C	0.339	0.073	0.014	7150	4.04E-06	0.0030	21.27
Triacylglycerol (48:0) levels	rs10218823	1	6029418	T	G	-0.352	0.076	0.017	5463	3.63E-06	0.0039	21.49

Triacylglycerol (48:0) levels	rs969816	2	210779037	G	A	0.133	0.030	0.120	5463	9.09E-06	0.0036	19.72
Triacylglycerol (48:0) levels	rs4955801	3	179092711	C	T	-0.107	0.023	0.785	5463	4.09E-06	0.0039	21.26
Triacylglycerol (48:0) levels	rs140828820	4	34767471	G	A	0.424	0.095	0.011	5463	7.93E-06	0.0036	19.99
Triacylglycerol (48:0) levels	rs62348866	4	186453244	A	G	-0.141	0.030	0.118	5463	2.01E-06	0.0041	22.62
Triacylglycerol (48:0) levels	rs7721676	5	149703644	C	T	-0.104	0.023	0.218	5463	8.45E-06	0.0036	19.86
Triacylglycerol (48:0) levels	rs79159286	7	31677697	G	T	0.163	0.036	0.080	5463	5.20E-06	0.0038	20.79
Triacylglycerol (48:0) levels	rs117945317	8	11538263	A	T	-0.475	0.107	0.009	5463	9.91E-06	0.0036	19.56
Triacylglycerol (48:0) levels	rs2242104	9	2640492	G	A	-0.103	0.022	0.752	5463	4.16E-06	0.0039	21.22
Triacylglycerol (48:0) levels	rs117264209	9	135992678	G	C	-0.331	0.072	0.019	5463	4.84E-06	0.0038	20.93
Triacylglycerol (48:0) levels	rs73302224	10	48593367	C	T	0.518	0.116	0.007	5463	7.88E-06	0.0036	19.99
Triacylglycerol (48:0) levels	rs60243822	12	81637055	C	A	0.114	0.026	0.172	5463	7.93E-06	0.0036	19.99
Triacylglycerol (48:0) levels	rs222507	12	101725095	T	G	-0.323	0.070	0.979	5463	3.68E-06	0.0039	21.46
Triacylglycerol (48:0) levels	rs101410	14	36678136	C	G	-	0.02	0.61	546	5.80E-	0.003	20.58

	87					0.089	0	5	3	06	8	
Triacylglycerol (48:0) levels	rs769432	17	3420961	T	C	0.091	0.019	0.509	5463	2.04E-06	0.0041	22.60
Triacylglycerol (48:0) levels	rs6504835	17	53554728	A	T	-0.087	0.020	0.412	5463	9.96E-06	0.0036	19.55
Triacylglycerol (48:0) levels	rs3894049	18	79524930	G	C	0.088	0.020	0.522	5463	8.06E-06	0.0036	19.95
Triacylglycerol (48:0) levels	rs10425025	19	9009145	A	T	0.305	0.066	0.023	5463	4.55E-06	0.0038	21.05
Triacylglycerol (48:0) levels	rs76375876	19	49524378	T	C	0.296	0.063	0.026	5463	2.78E-06	0.0040	22.00
Triacylglycerol (48:0) levels	rs2180528	20	15445391	T	C	0.096	0.020	0.389	5463	9.31E-07	0.0044	24.11
Triacylglycerol (48:0) levels	rs62235973	22	37593713	A	G	0.241	0.051	0.037	5463	2.54E-06	0.0040	22.17
Triacylglycerol (51:2) levels	rs4266911	1	27100305	C	T	0.160	0.036	0.941	7149	9.76E-06	0.0027	19.58
Triacylglycerol (51:2) levels	rs144241030	1	103243167	T	C	0.147	0.032	0.077	7149	5.05E-06	0.0029	20.84
Triacylglycerol (51:2) levels	rs1260326	2	27508073	C	T	-0.128	0.017	0.651	7149	2.46E-13	0.0075	53.79
Triacylglycerol (51:2) levels	rs76950187	2	128427075	G	A	0.163	0.032	0.074	7149	3.65E-07	0.0036	25.91
Triacylglycerol (51:2) levels	rs6721083	2	170225142	C	G	0.161	0.035	0.937	7149	4.43E-06	0.0029	21.09
Triacylglycerol (51:2) levels	rs17282355	3	134977489	T	C	-0.116	0.026	0.119	7149	1.00E-05	0.0027	19.53
Triacylglycerol (51:2) levels	rs139499	4	120830005	C	G	-	0.13	0.00	714	6.00E-	0.002	20.51

	638					0.617	6	4	9	06	9	
Triacylglycerol (51:2) levels	rs28437809	4	182501447	G	T	0.075	0.017	0.516	7149	8.29E-06	0.0028	19.89
Triacylglycerol (51:2) levels	rs143838492	5	114892988	A	G	0.681	0.148	0.003	7149	4.53E-06	0.0029	21.05
Triacylglycerol (51:2) levels	rs316343	6	2839825	T	C	-0.076	0.017	0.468	7149	9.37E-06	0.0027	19.66
Triacylglycerol (51:2) levels	rs33951980	7	73615107	T	C	-0.136	0.025	0.122	7149	9.09E-08	0.0040	28.61
Triacylglycerol (51:2) levels	rs117730598	9	2770506	C	T	0.231	0.050	0.030	7149	4.35E-06	0.0029	21.13
Triacylglycerol (51:2) levels	rs72753379	9	120350676	T	C	0.207	0.045	0.039	7149	3.94E-06	0.0030	21.32
Triacylglycerol (51:2) levels	rs28368829	9	136001733	A	G	-0.088	0.020	0.240	7149	8.07E-06	0.0028	19.94
Triacylglycerol (51:2) levels	rs112122274	9	136257994	G	A	-0.221	0.049	0.036	7149	5.64E-06	0.0029	20.63
Triacylglycerol (51:2) levels	rs174556	11	61813163	T	C	0.084	0.017	0.380	7149	1.08E-06	0.0033	23.81
Triacylglycerol (51:2) levels	rs964184	11	116778201	C	G	-0.212	0.023	0.849	7149	5.60E-20	0.0116	84.22
Triacylglycerol (51:2) levels	rs75573052	13	21038010	T	C	-0.135	0.030	0.085	7149	9.70E-06	0.0027	19.59
Triacylglycerol (51:2) levels	rs10147474	14	29823442	G	A	-0.22	0.045	0.037	7149	3.98E-07	0.0036	25.74

						7						
Triacylglycerol (51:2) levels	rs62043594	15	93154154	A	C	-0.080	0.017	0.418	7149	2.16E-06	0.0031	22.48
Triacylglycerol (51:2) levels	rs7412	19	44908822	T	C	0.168	0.037	0.053	7149	7.41E-06	0.0028	20.11
Triacylglycerol (58:7) levels	rs592586	1	109650768	G	A	0.091	0.019	0.595	5403	2.91E-06	0.0040	21.91
Triacylglycerol (58:7) levels	rs34621709	1	220806736	A	C	0.111	0.025	0.185	5403	8.13E-06	0.0037	19.94
Triacylglycerol (58:7) levels	rs1260326	2	27508073	C	T	-0.119	0.020	0.651	5403	2.66E-09	0.0065	35.52
Triacylglycerol (58:7) levels	rs7764213	6	112137404	C	T	0.108	0.024	0.195	5403	7.83E-06	0.0037	20.01
Triacylglycerol (58:7) levels	rs117926062	6	138930517	T	G	-0.454	0.101	0.009	5403	7.65E-06	0.0037	20.05
Triacylglycerol (58:7) levels	rs12700064	7	19617082	A	G	0.158	0.033	0.900	5403	2.12E-06	0.0042	22.52
Triacylglycerol (58:7) levels	rs15285	8	19967156	T	C	-0.123	0.022	0.261	5403	1.99E-08	0.0058	31.59
Triacylglycerol (58:7) levels	rs3011337	10	120774281	G	A	0.099	0.021	0.673	5403	1.85E-06	0.0042	22.78
Triacylglycerol (58:7) levels	rs102275	11	61790331	C	T	-0.159	0.020	0.410	5403	5.95E-16	0.0120	65.83
Triacylglycerol (58:7) levels	rs145956593	11	88458623	A	G	-0.122	0.028	0.141	5403	8.97E-06	0.0036	19.75
Triacylglycerol (58:7) levels	rs964184	11	116778201	C	G	-	0.02	0.84	540	1.38E-	0.011	64.14

						0.210	6	9	3	15	7	
Triacylglycerol (58:7) levels	rs1258712	12	31313616	T	G	0.180	0.039	0.931	5403	4.08E-06	0.0039	21.26
Triacylglycerol (58:7) levels	rs75214296	12	43836589	G	A	0.125	0.028	0.138	5403	5.94E-06	0.0038	20.54
Triacylglycerol (58:7) levels	rs141765432	14	76516128	T	G	-0.263	0.053	0.035	5403	8.36E-07	0.0045	24.32
Triacylglycerol (58:7) levels	rs67076780	18	78936828	T	C	-0.282	0.063	0.024	5403	6.79E-06	0.0037	20.28
Triacylglycerol (58:7) levels	rs187429064	19	19269704	G	A	-0.216	0.045	0.054	5403	1.60E-06	0.0043	23.06
Triacylglycerol (58:7) levels	rs111473971	19	21652047	A	G	0.138	0.030	0.121	5403	5.11E-06	0.0038	20.83
Triacylglycerol (58:7) levels	rs445925	19	44912383	A	G	0.221	0.035	0.077	5403	5.08E-10	0.0071	38.77
Triacylglycerol (58:7) levels	rs62209231	20	13843242	C	T	0.088	0.020	0.354	5403	9.08E-06	0.0036	19.72
Triacylglycerol (58:7) levels	rs371927	20	44634395	A	G	-0.177	0.037	0.915	5403	1.50E-06	0.0043	23.18
Triacylglycerol (58:7) levels	rs150578762	22	28083968	A	G	0.155	0.033	0.094	5403	2.52E-06	0.0041	22.19
Triacylglycerol (58:7) levels	rs1883350	22	43932163	C	T	0.114	0.020	0.391	5403	6.13E-09	0.0062	33.89

R2 refers to the proportion of variance explained for the association between the SNPs and the exposure variable. The calculation formula is R2 = 2*β2*EAF*(1-EAF)/(2*β2*EAF*(1-EAF) + 2*SE2*N*EAF*(1-EAF)).

F refers to the F-statistic. The calculation formula is F = R2*(N-2)/(1-R2), where F < 10 indicated a weak instrument variant.

N refers to the sample size of the initial GWAS from which the genetic instruments were selected.

Abbreviations: SNPs: single nucleotide polymorphisms; EAF: effect allele frequency; SE: standard error.

Supplementary table S4. Summary information for SNPs that were used as genetic instruments for Mendelian randomization analyses of inflammatory proteins.

Phenotype	SNP	chromosome	base_pair_location	effect_allele	other_allele	BETA	SE	EAF	p-value	N	R2	F
C-C motif chemokine 25 levels	rs11589432	1	23748852	C	G	0.057	0.012	0.478	1.97E-06	14287	0.0016	22.62
C-C motif chemokine 25 levels	rs143132666	1	160762078	A	C	-0.193	0.041	0.023	1.90E-06	14734	0.0015	22.69
C-C motif chemokine 25 levels	rs142015555	1	243141624	A	T	-0.238	0.052	0.021	5.62E-06	12935	0.0016	20.61
C-C motif chemokine 25 levels	rs6744934	2	115179913	T	C	-0.054	0.012	0.621	8.23E-06	14288	0.0014	19.88
C-C motif chemokine 25 levels	rs149395110	2	130959219	T	C	-0.278	0.061	0.013	4.92E-06	12880	0.0016	20.86
C-C motif chemokine 25 levels	rs9825982	3	132612790	A	G	0.059	0.013	0.356	9.16E-06	12196	0.0016	19.68
C-C motif chemokine 25 levels	rs7611785	3	144213414	A	G	0.094	0.020	0.103	2.01E-06	14736	0.0015	22.58
C-C motif chemokine 25 levels	rs851850	6	67085077	A	G	-0.078	0.016	0.846	1.87E-06	14288	0.0016	22.72
C-C motif chemokine 25 levels	rs142613772	6	161449388	T	TG	0.102	0.022	0.900	4.79E-06	11324	0.0018	20.92
C-C motif chemokine 25 levels	rs7806274	7	155146782	T	C	-0.076	0.016	0.167	2.74E-06	13667	0.0016	21.99
C-C motif chemokine 25 levels	rs111437511	8	10694001	T	C	0.144	0.030	0.043	1.31E-06	14733	0.0016	23.41
C-C motif chemokine 25 levels	rs1705699	8	23781453	T	C	-0.055	0.012	0.545	2.52E-06	14714	0.0015	22.15
C-C motif chemokine 25 levels	rs12114935	8	42100809	A	G	0.077	0.014	0.786	6.11E-08	14732	0.0020	29.32

C-C motif chemokine 25 levels	rs141247883	8	144986071	A	G	-0.580	0.130	0.992	8.05E-06	6780	0.0029	19.92
C-C motif chemokine 25 levels	rs11140450	9	86838024	A	T	-0.268	0.058	0.969	3.65E-06	10797	0.0020	21.44
C-C motif chemokine 25 levels	rs116534774	9	125200862	T	C	0.372	0.078	0.992	1.72E-06	11139	0.0021	22.88
C-C motif chemokine 25 levels	rs635634	9	136155000	T	C	-0.248	0.017	0.188	1.52E-50	11785	0.0186	223.52
C-C motif chemokine 25 levels	rs79990760	10	29446793	A	G	0.219	0.048	0.983	4.52E-06	14725	0.0014	21.03
C-C motif chemokine 25 levels	rs11028036	11	24600146	A	G	0.055	0.012	0.571	3.06E-06	14728	0.0015	21.77
C-C motif chemokine 25 levels	rs200184122	11	50675038	T	C	-0.082	0.018	0.698	4.96E-06	9830	0.0021	20.85
C-C motif chemokine 25 levels	rs10792444	11	64434151	T	C	-0.087	0.018	0.884	1.76E-06	14732	0.0015	22.84
C-C motif chemokine 25 levels	rs636479	11	75419643	A	G	0.055	0.012	0.376	4.12E-06	14731	0.0014	21.20
C-C motif chemokine 25 levels	rs7296588	12	578100	A	G	-0.116	0.012	0.437	1.66E-23	14725	0.0067	99.81
C-C motif chemokine 25 levels	rs7137942	12	81177765	A	G	-0.064	0.013	0.650	1.67E-06	12404	0.0018	22.94
C-C motif chemokine 25 levels	rs34520165	12	119526244	T	C	0.055	0.012	0.411	6.54E-06	14727	0.0014	20.32
C-C motif chemokine 25 levels	rs521795	13	22284926	T	C	-0.062	0.012	0.384	3.14E-07	14288	0.0018	26.16
C-C motif chemokine 25 levels	rs9530655	13	78006196	A	G	0.154	0.034	0.034	7.23E-06	14724	0.0014	20.13
C-C motif chemokine 25 levels	rs146498677	13	94811031	C	G	0.229	0.051	0.017	6.58E-06	13422	0.0015	20.31
C-C motif chemokine 25 levels	rs147851492	15	56638156	A	G	0.213	0.045	0.022	2.17E-06	14727	0.0015	22.44

C-C motif chemokine 25 levels	rs28494158	15	82219411	T	C	-0.073	0.016	0.834	5.29E-06	13867	0.0015	20.72
C-C motif chemokine 25 levels	rs12606983	18	2511738	T	C	-0.067	0.013	0.248	5.10E-07	14715	0.0017	25.22
C-C motif chemokine 25 levels	rs7252764	19	7827137	T	C	0.232	0.030	0.047	3.61E-15	14736	0.0042	61.89
C-C motif chemokine 25 levels	rs149462828	19	7901618	A	G	0.302	0.052	0.017	7.01E-09	14288	0.0023	33.53
C-C motif chemokine 25 levels	rs78865157	19	8107212	A	G	-0.392	0.045	0.021	2.13E-18	14726	0.0052	76.55
C-C motif chemokine 25 levels	rs3136647	19	8115457	A	G	0.768	0.015	0.170	0	14288	0.1604	2729.86
C-C motif chemokine 25 levels	rs61345716	19	8259301	T	C	-0.105	0.021	0.104	7.68E-07	14288	0.0017	24.43
C-C motif chemokine 25 levels	rs516316	19	49206145	C	G	-0.328	0.011	0.445	6.20E-182	14734	0.0532	827.21
C-C motif chemokine 25 levels	rs2317999	20	1689636	A	G	-0.058	0.013	0.457	9.06E-06	11760	0.0017	19.70
Interleukin-7 levels	rs77336315	1	97287518	A	C	0.251	0.053	0.984	2.24E-06	13801	0.0016	22.38
Interleukin-7 levels	rs56889120	1	174141356	A	G	0.097	0.021	0.874	3.86E-06	11340	0.0019	21.33
Interleukin-7 levels	rs74131483	1	194276216	A	G	0.137	0.027	0.945	5.52E-07	13240	0.0019	25.07
Interleukin-7 levels	rs17161952	1	223649878	T	C	0.093	0.020	0.903	5.27E-06	14734	0.0014	20.74
Interleukin-7 levels	rs144728120	3	35502404	T	C	0.150	0.032	0.044	2.23E-06	14717	0.0015	22.38
Interleukin-7 levels	rs116415547	4	110732727	T	G	0.209	0.046	0.020	5.36E-06	13870	0.0015	20.70
Interleukin-7 levels	rs139102025	4	147814894	C	G	-0.149	0.034	0.038	9.41E-06	12846	0.0015	19.62

Interleukin-7 levels	rs62338232	4	185760185	A	G	-0.081	0.018	0.136	6.28E-06	14288	0.0014	20.40
Interleukin-7 levels	rs11742240	5	35881376	T	G	-0.065	0.013	0.280	7.86E-07	14736	0.0017	24.39
Interleukin-7 levels	rs1050152	5	131676320	T	C	-0.065	0.013	0.412	9.84E-07	11785	0.0020	23.95
Interleukin-7 levels	rs202212924	5	133893338	A	G	-0.071	0.016	0.209	7.32E-06	12202	0.0016	20.11
Interleukin-7 levels	rs62387962	5	157298830	T	G	-0.078	0.018	0.135	9.34E-06	14288	0.0014	19.64
Interleukin-7 levels	rs12699881	7	2401569	A	G	0.068	0.014	0.252	8.33E-07	14288	0.0017	24.28
Interleukin-7 levels	rs78939185	7	129478199	C	G	0.213	0.048	0.021	9.14E-06	11775	0.0017	19.68
Interleukin-7 levels	rs1375955	8	106555039	T	G	0.061	0.012	0.401	4.06E-07	14288	0.0018	25.66
Interleukin-7 levels	rs7026771	9	133854336	A	G	0.069	0.015	0.568	3.03E-06	12403	0.0018	21.80
Interleukin-7 levels	rs12217463	10	16318694	T	C	-0.137	0.031	0.957	9.33E-06	14736	0.0013	19.64
Interleukin-7 levels	rs10734292	11	20486401	A	G	0.121	0.027	0.067	6.06E-06	11776	0.0017	20.47
Interleukin-7 levels	rs76213792	12	17798114	T	G	-0.279	0.062	0.011	5.87E-06	14288	0.0014	20.53
Interleukin-7 levels	rs11171132	12	55251385	T	C	-0.072	0.016	0.834	8.92E-06	14288	0.0014	19.73
Interleukin-7 levels	rs12321566	12	97807415	A	G	0.070	0.014	0.237	5.12E-07	14287	0.0018	25.21
Interleukin-7 levels	rs11158239	14	59452945	T	G	0.097	0.022	0.081	8.71E-06	14288	0.0014	19.77
Interleukin-7 levels	rs79072837	15	81321256	T	C	-0.136	0.028	0.050	1.19E-06	14715	0.0016	23.59

Interleukin-7 levels	rs1040320 1	19	14764810	A	G	- 0.058	0.01 3	0.29 2	4.26E- 06	1473 0	0.001 4	21.14

R2 refers to the proportion of variance explained for the association between the SNPs and the exposure variable. The calculation formula is R2 = 2*β2*EAF*(1-EAF)/(2*β2*EAF*(1-EAF) + 2*SE2*N*EAF*(1-EAF))

F refers to the F-statistic. The calculation formula is F = R2*(N-2)/(1-R2), where F < 10 indicated a weak instrument variant.

N refers to the sample size of the initial GWAS from which the genetic instruments were selected.

Abbreviations: SNPs: single nucleotide polymorphisms; EAF: effect allele frequency; SE: standard error.

Supplementary table S5. Summary information for SNPs that were used as genetic instruments for Mendelian randomization analyses of lung carcinoma and subtypes.

Phenotype	SNP	effect_allele	other_allele	BETA	SE	EAF	p-value	N	R2	F
Lung carcinoma	rs71658797	A	T	0.128	0.019	0.103	3.25E-11	85716	0.0005	44.02
Lung carcinoma	rs7705526	A	C	0.117	0.013	0.340	1.01E-18	85716	0.0009	78.03
Lung carcinoma	rs380286	A	G	-0.141	0.012	0.423	1.51E-32	85716	0.0016	141.13
Lung carcinoma	rs2893843	G	A	-0.072	0.012	0.392	2.03E-09	85716	0.0004	35.95
Lung carcinoma	rs501942	T	C	0.170	0.019	0.099	8.40E-19	85716	0.0009	78.40
Lung carcinoma	rs239935	G	A	0.067	0.012	0.478	1.29E-08	85716	0.0004	32.35
Lung carcinoma	rs11780471	A	G	-0.141	0.025	0.060	1.69E-08	85716	0.0004	31.82
Lung carcinoma	rs1629083	C	T	0.067	0.012	0.498	1.25E-08	85716	0.0004	32.41
Lung carcinoma	rs7953330	C	G	-0.087	0.013	0.312	6.10E-12	85716	0.0006	47.30
Lung carcinoma	rs11571833	T	A	0.472	0.058	0.011	6.12E-16	85716	0.0008	65.39
Lung carcinoma	rs4774488	C	T	0.066	0.012	0.404	3.60E-08	85716	0.0004	30.35
Lung carcinoma	rs77468143	G	T	-0.083	0.014	0.254	1.00E-09	85716	0.0004	37.32
Lung carcinoma	rs55781567	G	C	0.260	0.012	0.367	3.08E-103	85716	0.0054	465.51
Lung carcinoma	rs151118057	A	G	-0.294	0.051	0.018	9.56E-09	85716	0.0004	32.93
Lung carcinoma	rs56113850	T	C	-0.123	0.014	0.440	5.02E-19	85716	0.0009	79.41
Lung adenocarcinoma	rs71658797	A	T	0.169	0.027	0.103	3.12E-10	66756	0.0006	39.60
Lung adenocarcinoma	rs13080835	T	G	-0.111	0.016	0.493	7.45E-12	66756	0.0007	46.91
Lung adenocarcinoma	rs7705526	A	C	0.222	0.018	0.342	3.80E-35	66756	0.0023	153.01

Lung adenocarcinoma	rs421629	A	G	-0.157	0.016	0.427	9.75E-22	66756	0.0014	91.77
Lung adenocarcinoma	rs4236709	G	A	0.124	0.019	0.218	1.28E-10	66756	0.0006	41.34
Lung adenocarcinoma	rs885518	G	A	0.155	0.025	0.101	9.96E-10	66756	0.0006	37.33
Lung adenocarcinoma	rs62560775	G	A	0.166	0.028	0.107	2.23E-09	66756	0.0005	35.76
Lung adenocarcinoma	rs11591710	C	A	0.151	0.023	0.137	6.30E-11	66756	0.0006	42.72
Lung adenocarcinoma	rs1056562	T	C	0.102	0.016	0.473	2.76E-10	66756	0.0006	39.84
Lung adenocarcinoma	rs77468143	G	T	-0.155	0.019	0.253	1.69E-16	66756	0.0010	67.94
Lung adenocarcinoma	rs55781567	G	C	0.244	0.017	0.359	2.83E-48	66756	0.0032	213.12
Lung adenocarcinoma	rs56113850	T	C	-0.114	0.019	0.445	8.64E-10	66756	0.0006	37.61
Lung adenocarcinoma	rs41309931	T	G	0.157	0.026	0.117	1.31E-09	66756	0.0006	36.80
Squamous cell lung carcinoma	rs467095	C	T	-0.179	0.019	0.427	6.73E-21	63053	0.0014	87.95
Squamous cell lung carcinoma	rs501942	T	C	0.260	0.031	0.097	3.06E-17	63053	0.0011	71.30
Squamous cell lung carcinoma	rs9273429	G	A	-0.134	0.024	0.337	2.03E-08	63053	0.0005	31.46
Squamous cell lung carcinoma	rs7953330	C	G	-0.146	0.020	0.315	7.26E-13	63053	0.0008	51.47
Squamous cell lung carcinoma	rs11571818	C	T	0.754	0.094	0.011	9.77E-16	63053	0.0010	64.47
Squamous cell lung carcinoma	rs8040868	C	T	0.255	0.019	0.414	2.50E-41	63053	0.0029	181.32
Squamous cell lung carcinoma	rs56113850	T	C	-0.145	0.022	0.445	7.18E-11	63053	0.0007	42.47
Small cell lung carcinoma	rs11571833	T	A	0.786	0.142	0.010	2.82E-08	24108	0.0013	30.83
Small cell lung carcinoma	rs55853698	G	T	0.288	0.031	0.360	4.78E-21	24108	0.0037	88.61

R2 refers to the proportion of variance explained for the association between the SNPs and the exposure variable. The calculation formula is R2 = 2*β2*EAF*(1-EAF)/(2*β2*EAF*(1-EAF) + 2*SE2*N*EAF*(1-EAF))

F refers to the F-statistic. The calculation formula is F = R2*(N-2)/(1-R2), where F < 10 indicated a weak instrument variant.

N refers to the sample size of the initial GWAS from which the genetic instruments were selected.

Abbreviations: SNPs: single nucleotide polymorphisms; EAF: effect allele frequency; SE: standard error.

Supplementary table S6. Association of SNPs for plasma lipidome with lung carcinoma or subtypes using MR with different methods.

Exposure	Outcome	method	nsnp	or	or_lci95	or_uci95	pval
		MR Egger	25	0.959	0.847	1.087	0.521
		Weighted median	25	0.922	0.857	0.991	0.027

Sterol ester (27:1/20:2) levels	Lung carcinoma	Inverse variance weighted	25	0.922	0.876	0.971	0.002
		Simple mode	25	0.905	0.805	1.019	0.111
		Weighted mode	25	0.926	0.852	1.005	0.079
		MR Egger	16	1.137	0.961	1.343	0.156
		Weighted median	16	1.068	0.971	1.175	0.175
Diacylglycerol (16:1_18:1) levels	Lung carcinoma	Inverse variance weighted	16	1.107	1.034	1.186	0.004
		Simple mode	16	1.090	0.930	1.277	0.305
		Weighted mode	16	1.073	0.940	1.226	0.313
		MR Egger	16	0.955	0.849	1.075	0.458
		Weighted median	16	0.924	0.841	1.015	0.099
Phosphatidylcholine (O-16:1_18:0) levels	Lung carcinoma	Inverse variance weighted	16	0.912	0.856	0.972	0.005
		Simple mode	16	0.882	0.754	1.032	0.139
		Weighted mode	16	0.962	0.855	1.082	0.528
		MR Egger	20	1.097	1.010	1.192	0.042
		Weighted median	20	1.089	1.025	1.156	0.005
Phosphatidylcholine (16:0_20:5) levels	Lung carcinoma	Inverse variance weighted	20	1.070	1.021	1.122	0.005
		Simple mode	20	1.075	0.943	1.225	0.293
		Weighted mode	20	1.090	1.021	1.163	0.018
		MR Egger	21	1.063	1.010	1.119	0.030
		Weighted median	21	1.058	1.019	1.099	0.003
Phosphatidylcholine (16:0_20:4) levels	Lung carcinoma	Inverse variance weighted	21	1.045	1.010	1.082	0.012
		Simple mode	21	1.035	0.912	1.174	0.604
		Weighted mode	21	1.058	1.018	1.099	0.009
		MR Egger	15	0.928	0.830	1.036	0.208
		Weighted median	15	0.927	0.864	0.995	0.035
Phosphatidylcholine (18:0_20:2) levels	Lung carcinoma	Inverse variance weighted	15	0.935	0.886	0.986	0.013
		Simple mode	15	0.962	0.841	1.101	0.584
		Weighted mode	15	0.905	0.839	0.977	0.023
		MR Egger	25	1.001	0.846	1.186	0.987

		Weighted median	25	1.140	1.049	1.240	0.002
Phosphatidylethanolamine (O-18:1_20:4)	Lung carcinoma	Inverse variance weighted	25	1.096	1.019	1.180	0.014
		Simple mode	25	1.053	0.873	1.270	0.596
		Weighted mode	25	1.127	1.031	1.233	0.015
		MR Egger	24	1.091	0.962	1.239	0.190
		Weighted median	24	1.079	1.000	1.165	0.051
Phosphatidylcholine (18:0_20:3) levels	Lung carcinoma	Inverse variance weighted	24	1.067	1.012	1.125	0.016
		Simple mode	24	1.095	0.964	1.243	0.174
		Weighted mode	24	1.092	0.994	1.201	0.079
		MR Egger	20	1.008	0.864	1.175	0.924
		Weighted median	20	1.094	1.003	1.194	0.042
Sphingomyelin (d36:2) levels	Lung carcinoma	Inverse variance weighted	20	1.085	1.013	1.163	0.020
		Simple mode	20	1.145	0.962	1.362	0.144
		Weighted mode	20	1.114	0.966	1.284	0.153
		MR Egger	16	0.900	0.825	0.981	0.031
		Weighted median	16	0.931	0.876	0.989	0.020
Phosphatidylcholine (18:1_18:2) levels	Lung carcinoma	Inverse variance weighted	16	0.948	0.905	0.994	0.026
		Simple mode	16	0.996	0.879	1.129	0.950
		Weighted mode	16	0.918	0.861	0.978	0.018
		MR Egger	27	0.975	0.895	1.062	0.568
		Weighted median	27	0.975	0.917	1.036	0.406
Phosphatidylethanolamine (18:0_18:2)	Lung carcinoma	Inverse variance weighted	27	0.954	0.915	0.995	0.027
		Simple mode	27	0.943	0.845	1.052	0.302
		Weighted mode	27	0.958	0.903	1.018	0.176
		MR Egger	18	1.143	1.026	1.274	0.028
		Weighted median	18	1.099	1.023	1.180	0.009
Phosphatidylcholine (O-18:0_20:4) levels	Lung carcinoma	Inverse variance weighted	18	1.067	1.008	1.131	0.027
		Simple mode	18	1.063	0.917	1.231	0.428
		Weighted mode	18	1.102	1.019	1.190	0.026

		MR Egger	19	1.116	0.980	1.271	0.115
		Weighted median	19	1.092	1.005	1.187	0.038
Phosphatidylcholine (O-18:2_20:4) levels	Lung carcinoma	Inverse variance weighted	19	1.071	1.007	1.138	0.028
		Simple mode	19	1.156	1.007	1.328	0.055
		Weighted mode	19	1.161	1.035	1.301	0.020
		MR Egger	20	0.913	0.773	1.080	0.303
		Weighted median	20	0.888	0.815	0.968	0.007
Phosphatidylcholine (O-18:1_20:3) levels	Lung carcinoma	Inverse variance weighted	20	0.923	0.858	0.993	0.032
		Simple mode	20	0.872	0.748	1.017	0.097
		Weighted mode	20	0.884	0.803	0.974	0.021
		MR Egger	20	1.087	0.976	1.212	0.146
		Weighted median	20	1.085	1.026	1.147	0.004
Phosphatidylcholine (O-16:1_20:4) levels	Lung carcinoma	Inverse variance weighted	20	1.063	1.005	1.125	0.032
		Simple mode	20	1.063	0.927	1.220	0.392
		Weighted mode	20	1.072	1.016	1.131	0.021
		MR Egger	24	1.066	0.955	1.189	0.270
		Weighted median	24	1.119	1.029	1.217	0.008
Sphingomyelin (d38:2) levels	Lung carcinoma	Inverse variance weighted	24	1.066	1.004	1.131	0.036
		Simple mode	24	1.131	0.990	1.292	0.084
		Weighted mode	24	1.131	1.017	1.257	0.032
		MR Egger	16	1.060	0.818	1.373	0.668
		Weighted median	16	0.957	0.854	1.072	0.448
Phosphatidylcholine (16:0_16:1) levels*	Lung carcinoma	Inverse variance weighted	16	0.920	0.850	0.996	0.040
		Simple mode	16	1.015	0.835	1.233	0.884
		Weighted mode	16	0.984	0.825	1.173	0.859
		MR Egger	24	1.058	1.004	1.115	0.048
		Weighted median	24	1.056	1.018	1.095	0.004
Phosphatidylcholine (18:0_20:4) levels	Lung carcinoma	Inverse variance weighted	24	1.039	1.000	1.078	0.047
		Simple mode	24	1.034	0.895	1.195	0.653

		Weighted mode	24	1.056	1.018	1.094	0.007
		MR Egger	27	1.073	1.016	1.134	0.018
		Weighted median	27	1.064	1.020	1.111	0.004
Phosphatidylcholine (17:0_20:4) levels	Lung carcinoma	Inverse variance weighted	27	1.037	1.000	1.076	0.049
		Simple mode	27	1.017	0.903	1.146	0.779
		Weighted mode	27	1.058	1.012	1.106	0.019
		MR Egger	26	1.173	1.041	1.321	0.015
		Weighted median	26	1.122	1.017	1.237	0.022
Sphingomyelin (d34:2) levels	Lung adenocarcinoma	Inverse variance weighted	26	1.124	1.051	1.203	0.001
		Simple mode	26	1.163	0.997	1.357	0.066
		Weighted mode	26	1.149	1.026	1.286	0.024
		MR Egger	23	0.939	0.843	1.045	0.261
		Weighted median	23	0.927	0.852	1.009	0.078
Phosphatidylcholine (16:0_20:2) levels	Lung adenocarcinoma	Inverse variance weighted	23	0.924	0.870	0.981	0.010
		Simple mode	23	0.935	0.819	1.068	0.332
		Weighted mode	23	0.918	0.826	1.020	0.124
		MR Egger	20	1.197	0.848	1.691	0.321
		Weighted median	20	1.142	1.021	1.278	0.021
Triacylglycerol (58:7) levels	Lung adenocarcinoma	Inverse variance weighted	20	1.124	1.023	1.234	0.015
		Simple mode	20	1.111	0.910	1.355	0.314
		Weighted mode	20	1.147	0.965	1.363	0.136
		MR Egger	22	1.116	0.999	1.247	0.065
		Weighted median	22	1.092	1.004	1.188	0.041
Phosphatidylcholine (16:0_20:5) levels	Lung adenocarcinoma	Inverse variance weighted	22	1.079	1.012	1.150	0.020
		Simple mode	22	1.098	0.943	1.279	0.242
		Weighted mode	22	1.091	0.998	1.193	0.068
		MR Egger	19	1.187	1.033	1.362	0.027
		Weighted median	19	1.122	1.019	1.236	0.019
Phosphatidylcholine (O-18:0_20:4) levels	Lung adenocarcinoma	Inverse variance weighted	19	1.088	1.011	1.171	0.023

		Simple mode	19	1.251	1.014	1.543	0.052
		Weighted mode	19	1.148	1.032	1.278	0.021
		MR Egger	16	0.903	0.775	1.051	0.208
		Weighted median	16	0.891	0.804	0.987	0.027
Phosphatidylcholine (18:0_20:2) levels	Lung adenocarcinoma	Inverse variance weighted	16	0.920	0.856	0.989	0.024
		Simple mode	16	0.934	0.779	1.120	0.472
		Weighted mode	16	0.903	0.810	1.008	0.088
		MR Egger	21	1.397	1.118	1.746	0.008
		Weighted median	21	1.169	1.040	1.314	0.009
Phosphatidylcholine (O-16:1_20:3) levels	Lung adenocarcinoma	Inverse variance weighted	21	1.096	1.007	1.193	0.033
		Simple mode	21	1.066	0.859	1.322	0.569
		Weighted mode	21	1.178	1.004	1.383	0.059
		MR Egger	20	0.990	0.850	1.152	0.898
		Weighted median	20	0.937	0.844	1.041	0.225
Phosphatidylethanolamine (O-18:2_18:1)	Lung adenocarcinoma	Inverse variance weighted	20	0.921	0.851	0.996	0.040
		Simple mode	20	0.933	0.785	1.109	0.441
		Weighted mode	20	0.948	0.815	1.103	0.501
		MR Egger	25	1.016	0.865	1.193	0.852
		Weighted median	25	1.062	0.964	1.169	0.222
Diacylglycerol (16:0_18:2) levels	Lung adenocarcinoma	Inverse variance weighted	25	1.089	1.004	1.181	0.040
		Simple mode	25	1.053	0.888	1.250	0.557
		Weighted mode	25	1.050	0.923	1.194	0.466
		MR Egger	13	0.981	0.740	1.299	0.894
		Weighted median	13	0.934	0.817	1.069	0.323
Phosphatidylcholine (O-18:0_16:1) levels	Lung adenocarcinoma	Inverse variance weighted	13	0.905	0.822	0.996	0.041
		Simple mode	13	0.970	0.781	1.204	0.786
		Weighted mode	13	0.963	0.810	1.145	0.675
		MR Egger	25	0.939	0.807	1.092	0.420
		Weighted median	25	0.943	0.857	1.037	0.227

Sterol ester (27:1/20:2) levels	Lung adenocarcinoma	Inverse variance weighted	25	0.937	0.880	0.999	0.045
		Simple mode	25	0.874	0.744	1.027	0.116
		Weighted mode	25	0.938	0.842	1.045	0.257
		MR Egger	15	1.083	0.856	1.370	0.517
		Weighted median	15	1.096	0.966	1.244	0.154
Diacylglycerol (16:1_18:1) levels	Lung adenocarcinoma	Inverse variance weighted	15	1.103	1.002	1.214	0.046
		Simple mode	15	1.100	0.891	1.359	0.389
		Weighted mode	15	1.100	0.907	1.335	0.350
		MR Egger	24	1.189	0.998	1.416	0.065
		Weighted median	24	1.154	1.031	1.290	0.013
Phosphatidylcholine (18:0_20:3) levels	Lung adenocarcinoma	Inverse variance weighted	24	1.077	1.001	1.159	0.047
		Simple mode	24	1.174	0.961	1.434	0.130
		Weighted mode	24	1.188	1.026	1.376	0.031
		MR Egger	15	1.126	0.919	1.380	0.272
		Weighted median	15	1.099	0.972	1.243	0.132
Triacylglycerol (48:0) levels	Lung adenocarcinoma	Inverse variance weighted	15	1.093	1.000	1.194	0.050
		Simple mode	15	1.120	0.922	1.362	0.273
		Weighted mode	15	1.120	0.936	1.341	0.235
		MR Egger	24	1.154	0.949	1.403	0.166
		Weighted median	24	1.158	1.017	1.319	0.026
Phosphatidylethanolamine (O-18:1_20:4)	Squamous cell lung carcinoma	Inverse variance weighted	24	1.159	1.064	1.262	0.001
		Simple mode	24	1.075	0.870	1.328	0.512
		Weighted mode	24	1.140	0.993	1.310	0.076
		MR Egger	16	0.871	0.719	1.055	0.179
		Weighted median	16	0.865	0.754	0.993	0.039
Phosphatidylcholine (O-16:1_18:0) levels	Squamous cell lung carcinoma	Inverse variance weighted	16	0.852	0.769	0.943	0.002
		Simple mode	16	0.791	0.623	1.003	0.072
		Weighted mode	16	0.883	0.739	1.056	0.194
		MR Egger	16	0.896	0.751	1.069	0.243

		Weighted median	16	0.869	0.774	0.977	0.018
Phosphatidylcholine (18:0_20:2) levels	Squamous cell lung carcinoma	Inverse variance weighted	16	0.881	0.810	0.959	0.003
		Simple mode	16	0.811	0.674	0.977	0.044
		Weighted mode	16	0.861	0.759	0.976	0.034
		MR Egger	25	0.928	0.822	1.047	0.237
		Weighted median	25	0.923	0.850	1.003	0.058
Phosphatidylethanolamine (18:0_18:2)	Squamous cell lung carcinoma	Inverse variance weighted	25	0.914	0.860	0.971	0.004
		Simple mode	25	0.930	0.801	1.081	0.354
		Weighted mode	25	0.927	0.853	1.008	0.089
		MR Egger	22	1.026	0.828	1.271	0.816
		Weighted median	22	1.087	0.943	1.254	0.250
Sphingomyelin (d38:2) levels*	Squamous cell lung carcinoma	Inverse variance weighted	22	1.144	1.042	1.257	0.005
		Simple mode	22	1.282	0.968	1.699	0.098
		Weighted mode	22	0.955	0.736	1.239	0.733
		MR Egger	22	1.081	0.882	1.324	0.462
		Weighted median	22	1.166	1.026	1.326	0.019
Sphingomyelin (d34:0) levels	Squamous cell lung carcinoma	Inverse variance weighted	22	1.138	1.030	1.256	0.011
		Simple mode	22	1.120	0.894	1.402	0.335
		Weighted mode	22	1.165	1.000	1.359	0.064
		MR Egger	27	0.869	0.720	1.048	0.155
		Weighted median	27	0.915	0.806	1.038	0.168
Cholesterol levels	Squamous cell lung carcinoma	Inverse variance weighted	27	0.893	0.818	0.975	0.011
		Simple mode	27	0.984	0.783	1.238	0.893
		Weighted mode	27	0.943	0.789	1.127	0.526
		MR Egger	20	1.143	1.007	1.298	0.053
		Weighted median	20	1.096	1.003	1.197	0.043
Phosphatidylcholine (O-16:1_20:4) levels	Squamous cell lung carcinoma	Inverse variance weighted	20	1.089	1.018	1.165	0.013
		Simple mode	20	1.152	0.966	1.373	0.132
		Weighted mode	20	1.100	1.006	1.204	0.051

		MR Egger	16	0.807	0.674	0.967	0.036
		Weighted median	16	0.862	0.739	1.005	0.059
Phosphatidylcholine (17:0_18:1) levels	Squamous cell lung carcinoma	Inverse variance weighted	16	0.879	0.791	0.975	0.015
		Simple mode	16	0.793	0.623	1.009	0.079
		Weighted mode	16	0.859	0.718	1.027	0.116
		MR Egger	22	1.074	0.990	1.166	0.099
		Weighted median	22	1.072	1.005	1.142	0.035
Phosphatidylcholine (16:0_20:4) levels	Squamous cell lung carcinoma	Inverse variance weighted	22	1.070	1.013	1.131	0.015
		Simple mode	22	1.138	0.943	1.374	0.191
		Weighted mode	22	1.076	1.012	1.145	0.030
		MR Egger	21	0.855	0.663	1.103	0.243
		Weighted median	21	0.866	0.756	0.992	0.037
Phosphatidylcholine (18:1_18:1) levels	Squamous cell lung carcinoma	Inverse variance weighted	21	0.890	0.806	0.983	0.021
		Simple mode	21	0.898	0.665	1.212	0.489
		Weighted mode	21	0.855	0.717	1.019	0.096
		MR Egger	24	1.092	1.019	1.170	0.021
		Weighted median	24	1.080	1.020	1.143	0.008
Phosphatidylcholine (18:0_20:4) levels*	Squamous cell lung carcinoma	Inverse variance weighted	24	1.055	1.005	1.108	0.031
		Simple mode	24	0.945	0.774	1.154	0.582
		Weighted mode	24	1.083	1.025	1.144	0.009
		MR Egger	19	1.191	0.980	1.448	0.096
		Weighted median	19	1.219	1.074	1.384	0.002
Phosphatidylcholine (O-18:2_20:4) levels	Squamous cell lung carcinoma	Inverse variance weighted	19	1.106	1.008	1.214	0.033
		Simple mode	19	1.223	0.986	1.516	0.083
		Weighted mode	19	1.226	1.039	1.446	0.027
		MR Egger	26	0.889	0.632	1.251	0.505
		Weighted median	26	1.097	0.947	1.272	0.218
Sphingomyelin (d36:1) levels*	Squamous cell lung carcinoma	Inverse variance weighted	26	1.129	1.006	1.266	0.039
		Simple mode	26	1.157	0.870	1.539	0.326

		Weighted mode	26	1.108	0.867	1.416	0.421
		MR Egger	19	1.008	0.753	1.349	0.959
		Weighted median	19	0.953	0.830	1.095	0.499
Phosphatidylcholine (O-16:0_18:1) levels*	Squamous cell lung carcinoma	Inverse variance weighted	19	0.901	0.816	0.996	0.041
		Simple mode	19	0.969	0.775	1.213	0.789
		Weighted mode	19	0.981	0.816	1.180	0.840
		MR Egger	21	0.980	0.695	1.380	0.908
		Weighted median	21	0.899	0.727	1.113	0.329
Phosphatidylinositol (18:1_20:4) levels	Small cell lung carcinoma	Inverse variance weighted	21	0.822	0.707	0.956	0.011
		Simple mode	21	0.943	0.655	1.357	0.753
		Weighted mode	21	0.934	0.672	1.298	0.689
		MR Egger	17	1.111	0.717	1.721	0.646
		Weighted median	17	1.059	0.844	1.330	0.619
Sphingomyelin (d36:2) levels	Small cell lung carcinoma	Inverse variance weighted	17	1.211	1.031	1.421	0.019
		Simple mode	17	1.068	0.717	1.591	0.749
		Weighted mode	17	1.047	0.760	1.443	0.782
		MR Egger	16	1.330	0.980	1.805	0.088
		Weighted median	16	1.085	0.878	1.341	0.449
Phosphatidylcholine (O-16:0_20:3) levels	Small cell lung carcinoma	Inverse variance weighted	16	1.180	1.021	1.364	0.025
		Simple mode	16	1.139	0.804	1.613	0.475
		Weighted mode	16	1.088	0.843	1.403	0.527
		MR Egger	15	1.194	0.911	1.565	0.221
		Weighted median	15	1.096	0.892	1.347	0.381
Phosphatidylcholine (O-18:0_14:0) levels	Small cell lung carcinoma	Inverse variance weighted	15	1.190	1.018	1.392	0.029
		Simple mode	15	1.121	0.812	1.548	0.499
		Weighted mode	15	1.104	0.866	1.408	0.438
		MR Egger	19	1.018	0.751	1.379	0.912
		Weighted median	19	0.876	0.706	1.087	0.229
Phosphatidylcholine (20:4_0:0) levels*	Small cell lung carcinoma	Inverse variance weighted	19	0.861	0.744	0.996	0.044

		Simple mode	19	0.843	0.573	1.239	0.396
		Weighted mode	19	0.869	0.609	1.240	0.449
		MR Egger	31	0.859	0.661	1.117	0.267
		Weighted median	31	0.845	0.717	0.996	0.045
Diacylglycerol (18:1_18:2) levels*	Small cell lung carcinoma	Inverse variance weighted	31	0.879	0.773	0.999	0.049
		Simple mode	31	1.201	0.861	1.675	0.289
		Weighted mode	31	0.864	0.699	1.067	0.185
		MR Egger	16	1.009	0.533	1.907	0.979
		Weighted median	16	0.833	0.651	1.066	0.146
Triacylglycerol (51:2) levels*	Small cell lung carcinoma	Inverse variance weighted	16	0.804	0.647	0.999	0.049
		Simple mode	16	0.870	0.589	1.285	0.494
		Weighted mode	16	0.845	0.620	1.153	0.306

*The result with additional MR method (MR-Egger, weighted median, simple mode, weighted mode) is not consistent with the direction of inverse variance weighted method.

Abbreviations: SNPs: single nucleotide polymorphisms.

Supplementary table S7. Association of SNPs for plasma lipidome with LC or subtypes using MR-BMA method.

Exposure	Outcome	or	or_lci95	or_uci95	pval
Sterol ester (27:1/20:2) levels	Lung carcinoma	0.917	0.874	0.963	0.001
Diacylglycerol (16:1_18:1) levels	Lung carcinoma	1.110	1.032	1.194	0.005
Phosphatidylcholine (16:0_20:5) levels	Lung carcinoma	1.071	1.020	1.124	0.005
Phosphatidylcholine (O-16:1_18:0) levels	Lung carcinoma	0.903	0.840	0.971	0.006
Phosphatidylcholine (18:0_20:2) levels	Lung carcinoma	0.933	0.880	0.988	0.018
Sphingomyelin (d36:2) levels	Lung carcinoma	1.092	1.014	1.177	0.021
Phosphatidylcholine (18:1_18:2) levels	Lung carcinoma	0.945	0.900	0.992	0.022
Phosphatidylcholine (O-18:2_20:4) levels	Lung carcinoma	1.073	1.009	1.140	0.025
Phosphatidylcholine (16:0_20:4) levels	Lung carcinoma	1.045	1.005	1.086	0.026
Phosphatidylcholine (O-16:1_20:4) levels	Lung carcinoma	1.057	1.002	1.116	0.041
Phosphatidylcholine (O-18:1_20:3) levels	Lung carcinoma	0.921	0.848	1.000	0.049

Phosphatidylcholine (17:0_20:4) levels	Lung carcinoma	1.038	1.000	1.077	0.049
Phosphatidylethanolamine (O-18:1_20:4)	Lung carcinoma	1.079	1.000	1.164	0.050
Phosphatidylethanolamine (18:0_18:2)	Lung carcinoma	0.956	0.913	1.000	0.052
Phosphatidylcholine (18:0_20:4) levels	Lung carcinoma	1.038	0.998	1.079	0.063
Phosphatidylcholine (18:0_20:3) levels	Lung carcinoma	1.057	0.996	1.122	0.067
Phosphatidylcholine (16:0_16:1) levels	Lung carcinoma	0.919	0.840	1.007	0.069
Sphingomyelin (d38:2) levels	Lung carcinoma	1.051	0.994	1.110	0.080
Phosphatidylcholine (O-18:0_20:4) levels	Lung carcinoma	1.050	0.973	1.133	0.209
Triacylglycerol (48:0) levels	Lung adenocarcinoma	1.131	1.035	1.237	0.007
Phosphatidylcholine (16:0_20:5) levels	Lung adenocarcinoma	1.091	1.022	1.163	0.008
Triacylglycerol (58:7) levels	Lung adenocarcinoma	1.133	1.024	1.254	0.015
Phosphatidylcholine (18:0_20:2) levels	Lung adenocarcinoma	0.906	0.835	0.983	0.018
Diacylglycerol (16:0_18:2) levels	Lung adenocarcinoma	1.099	1.014	1.191	0.022
Phosphatidylcholine (O-16:1_20:3) levels	Lung adenocarcinoma	1.107	1.012	1.211	0.027
Phosphatidylcholine (16:0_20:2) levels	Lung adenocarcinoma	0.936	0.879	0.996	0.036
Phosphatidylethanolamine (O-18:2_18:1)	Lung adenocarcinoma	0.914	0.839	0.996	0.039
Sterol ester (27:1/20:2) levels	Lung adenocarcinoma	0.935	0.876	0.999	0.046
Diacylglycerol (16:1_18:1) levels	Lung adenocarcinoma	1.106	1.000	1.223	0.049
Sphingomyelin (d34:2) levels	Lung adenocarcinoma	1.075	1.000	1.156	0.050
Phosphatidylcholine (O-18:0_16:1) levels	Lung adenocarcinoma	0.902	0.812	1.004	0.058
Phosphatidylcholine (18:0_20:3) levels	Lung adenocarcinoma	1.065	0.985	1.152	0.113
Phosphatidylcholine (O-18:0_20:4) levels	Lung adenocarcinoma	1.076	0.973	1.190	0.154
Phosphatidylethanolamine (O-18:1_20:4)	Squamous cell lung carcinoma	1.168	1.071	1.275	0.000
Phosphatidylcholine (18:0_20:2) levels	Squamous cell lung carcinoma	0.874	0.802	0.952	0.002
Phosphatidylethanolamine (18:0_18:2)	Squamous cell lung carcinoma	0.912	0.858	0.969	0.003
Phosphatidylcholine (O-16:1_18:0) levels	Squamous cell lung carcinoma	0.841	0.748	0.945	0.004
Phosphatidylcholine (O-16:1_20:4) levels	Squamous cell lung carcinoma	1.093	1.024	1.165	0.007
Phosphatidylcholine (16:0_20:4) levels	Squamous cell lung carcinoma	1.076	1.019	1.135	0.008
Sphingomyelin (d38:2) levels	Squamous cell lung carcinoma	1.114	1.025	1.211	0.011

Sphingomyelin (d34:0) levels	Squamous cell lung carcinoma	1.135	1.029	1.253	0.012
Phosphatidylcholine (18:1_18:1) levels	Squamous cell lung carcinoma	0.888	0.804	0.980	0.018
Phosphatidylcholine (17:0_18:1) levels	Squamous cell lung carcinoma	0.871	0.775	0.980	0.022
Phosphatidylcholine (18:0_20:4) levels	Squamous cell lung carcinoma	1.057	1.007	1.111	0.026
Phosphatidylcholine (O-16:0_18:1) levels	Squamous cell lung carcinoma	0.898	0.811	0.994	0.038
Phosphatidylcholine (O-18:2_20:4) levels	Squamous cell lung carcinoma	1.109	1.005	1.223	0.039
Sphingomyelin (d36:1) levels	Squamous cell lung carcinoma	1.117	1.004	1.243	0.042
Cholesterol levels	Squamous cell lung carcinoma	0.940	0.854	1.035	0.208
Phosphatidylinositol (18:1_20:4) levels	Small cell lung carcinoma	0.806	0.686	0.948	0.009
Sphingomyelin (d36:2) levels	Small cell lung carcinoma	1.226	1.034	1.453	0.019
Phosphatidylcholine (O-16:0_20:3) levels	Small cell lung carcinoma	1.188	1.019	1.385	0.028
Phosphatidylcholine (O-18:0_14:0) levels	Small cell lung carcinoma	1.210	1.018	1.438	0.030
Phosphatidylcholine (20:4_0:0) levels	Small cell lung carcinoma	0.857	0.733	1.002	0.053
Triacylglycerol (51:2) levels	Small cell lung carcinoma	0.807	0.642	1.014	0.066
Diacylglycerol (18:1_18:2) levels	Small cell lung carcinoma	0.883	0.771	1.012	0.074

Abbreviations: SNPs: single nucleotide polymorphisms; MR-BMA: MR Bayesian model averaging.

Supplementary table S8. Association of SNPs for lung carcinoma or subtypes with plasma lipidome using MR with different methods.

Exposure	Outcome	method	nsnp	or	or_lci95	or_uci95	pval
		MR Egger	15	0.990	0.787	1.247	0.936
		Weighted median	15	0.979	0.868	1.104	0.727
Lung carcinoma	Sterol ester (27:1/20:2) levels	Inverse variance weighted	15	0.957	0.862	1.064	0.417
		Simple mode	15	0.993	0.793	1.242	0.949
		Weighted mode	15	0.975	0.852	1.117	0.721
		MR Egger	15	0.904	0.693	1.178	0.468
		Weighted median	15	1.045	0.928	1.177	0.469
Lung carcinoma	Diacylglycerol (16:1_18:1) levels	Inverse variance weighted	15	0.987	0.873	1.117	0.840
		Simple mode	15	1.011	0.838	1.219	0.913
		Weighted mode	15	1.026	0.907	1.162	0.687

		MR Egger	15	0.906	0.747	1.099	0.335
		Weighted median	15	0.988	0.885	1.102	0.822
Lung carcinoma	Phosphatidylcholine (16:0_20:4) levels	Inverse variance weighted	15	0.979	0.894	1.072	0.649
		Simple mode	15	0.916	0.745	1.127	0.423
		Weighted mode	15	0.982	0.867	1.112	0.779
		MR Egger	15	0.908	0.731	1.127	0.398
		Weighted median	15	0.967	0.860	1.087	0.575
Lung carcinoma	Phosphatidylcholine (16:0_20:5) levels	Inverse variance weighted	15	0.917	0.831	1.012	0.086
		Simple mode	15	0.947	0.738	1.216	0.677
		Weighted mode	15	0.972	0.851	1.109	0.676
		MR Egger	15	0.876	0.733	1.047	0.168
		Weighted median	15	0.899	0.802	1.008	0.069
Lung carcinoma	Phosphatidylcholine (17:0_20:4) levels	Inverse variance weighted	15	0.943	0.867	1.026	0.170
		Simple mode	15	0.868	0.699	1.079	0.223
		Weighted mode	15	0.878	0.773	0.998	0.067
		MR Egger	15	0.958	0.797	1.151	0.654
		Weighted median	15	0.969	0.856	1.097	0.621
Lung carcinoma	Phosphatidylcholine (18:0_20:2) levels	Inverse variance weighted	15	0.973	0.893	1.061	0.537
		Simple mode	15	0.923	0.728	1.171	0.521
		Weighted mode	15	0.962	0.831	1.115	0.615
		MR Egger	15	0.898	0.763	1.057	0.219
		Weighted median	15	0.960	0.861	1.069	0.455
Lung carcinoma	Phosphatidylcholine (18:1_18:2) levels	Inverse variance weighted	15	0.973	0.900	1.051	0.479
		Simple mode	15	1.020	0.824	1.263	0.859
		Weighted mode	15	0.934	0.821	1.063	0.320
		MR Egger	15	0.958	0.809	1.134	0.627
		Weighted median	15	0.961	0.866	1.066	0.454
Lung carcinoma	Phosphatidylcholine (O-16:1_18:0) levels	Inverse variance weighted	15	0.981	0.906	1.062	0.635
		Simple mode	15	0.980	0.824	1.166	0.826

		Weighted mode	15	0.938	0.834	1.054	0.297
		MR Egger	15	0.991	0.787	1.248	0.941
		Weighted median	15	0.979	0.870	1.101	0.718
Lung carcinoma	Phosphatidylcholine (O-16:1_20:4) levels	Inverse variance weighted	15	1.037	0.933	1.153	0.499
		Simple mode	15	0.947	0.769	1.167	0.619
		Weighted mode	15	0.969	0.855	1.097	0.626
		MR Egger	15	1.023	0.865	1.209	0.794
		Weighted median	15	1.026	0.919	1.144	0.650
Lung carcinoma	Phosphatidylcholine (O-18:1_20:3) levels	Inverse variance weighted	15	1.045	0.966	1.131	0.274
		Simple mode	15	1.009	0.835	1.218	0.930
		Weighted mode	15	1.028	0.913	1.159	0.652
		MR Egger	15	1.144	0.930	1.408	0.226
		Weighted median	15	1.027	0.914	1.154	0.657
Lung carcinoma	Phosphatidylcholine (O-18:2_20:4) levels	Inverse variance weighted	15	0.966	0.869	1.074	0.523
		Simple mode	15	0.973	0.747	1.268	0.843
		Weighted mode	15	1.065	0.935	1.212	0.359
		MR Egger	15	1.067	0.849	1.340	0.589
		Weighted median	15	1.127	1.005	1.264	0.041
Lung carcinoma	Sphingomyelin (d36:2) levels	Inverse variance weighted	15	1.042	0.939	1.156	0.438
		Simple mode	15	1.075	0.831	1.391	0.589
		Weighted mode	15	1.116	0.982	1.268	0.115
		MR Egger	13	0.884	0.673	1.160	0.392
		Weighted median	13	0.947	0.857	1.047	0.292
Lung adenocarcinoma	Sterol ester (27:1/20:2) levels	Inverse variance weighted	13	0.950	0.881	1.024	0.179
		Simple mode	13	0.911	0.774	1.071	0.280
		Weighted mode	13	0.941	0.817	1.085	0.419
		MR Egger	13	0.988	0.744	1.313	0.936
		Weighted median	13	1.013	0.917	1.117	0.805
Lung adenocarcinoma	Diacylglycerol (16:0_18:2) levels	Inverse variance weighted	13	1.005	0.932	1.083	0.902

		Simple mode	13	0.974	0.839	1.130	0.730
		Weighted mode	13	1.019	0.898	1.158	0.773
		MR Egger	13	1.097	0.772	1.560	0.615
		Weighted median	13	1.033	0.935	1.142	0.521
Lung adenocarcinoma	Diacylglycerol (16:1_18:1) levels	Inverse variance weighted	13	1.017	0.926	1.117	0.730
		Simple mode	13	1.076	0.914	1.267	0.395
		Weighted mode	13	1.045	0.932	1.171	0.465
		MR Egger	13	0.897	0.702	1.147	0.405
		Weighted median	13	0.915	0.840	0.997	0.042
Lung adenocarcinoma	Phosphatidylcholine (16:0_20:2) levels	Inverse variance weighted	13	0.934	0.873	1.000	0.050
		Simple mode	13	0.963	0.842	1.101	0.589
		Weighted mode	13	0.915	0.818	1.022	0.142
		MR Egger	13	1.091	0.796	1.498	0.598
		Weighted median	13	0.994	0.897	1.102	0.907
Lung adenocarcinoma	Phosphatidylcholine (16:0_20:5) levels	Inverse variance weighted	13	0.956	0.877	1.042	0.304
		Simple mode	13	1.027	0.868	1.215	0.765
		Weighted mode	13	1.016	0.890	1.160	0.821
		MR Egger	13	0.957	0.744	1.232	0.741
		Weighted median	13	1.022	0.932	1.121	0.640
Lung adenocarcinoma	Phosphatidylcholine (O-16:1_20:3) levels	Inverse variance weighted	13	0.998	0.931	1.070	0.957
		Simple mode	13	1.035	0.894	1.197	0.656
		Weighted mode	13	1.021	0.905	1.153	0.740
		MR Egger	13	0.900	0.698	1.159	0.431
		Weighted median	13	0.997	0.912	1.090	0.956
Lung adenocarcinoma	Phosphatidylethanolamine (O-18:2_18:1)	Inverse variance weighted	13	0.982	0.915	1.053	0.605
		Simple mode	13	0.983	0.861	1.123	0.807
		Weighted mode	13	0.987	0.889	1.095	0.805
		MR Egger	13	0.918	0.694	1.216	0.565
		Weighted median	13	1.012	0.923	1.110	0.793

Lung adenocarcinoma	Sphingomyelin (d34:2) levels	Inverse variance weighted	13	1.009	0.935	1.088	0.823
		Simple mode	13	0.990	0.873	1.122	0.874
		Weighted mode	13	1.004	0.902	1.117	0.947
		MR Egger	13	0.967	0.704	1.327	0.838
		Weighted median	13	1.008	0.904	1.124	0.891
Lung adenocarcinoma	Triacylglycerol (48:0) levels	Inverse variance weighted	13	1.030	0.946	1.120	0.498
		Simple mode	13	0.973	0.808	1.172	0.780
		Weighted mode	13	0.989	0.865	1.130	0.869
		MR Egger	13	1.148	0.825	1.597	0.430
		Weighted median	13	0.971	0.865	1.090	0.618
Lung adenocarcinoma	Triacylglycerol (58:7) levels	Inverse variance weighted	13	1.002	0.916	1.097	0.964
		Simple mode	13	1.004	0.801	1.259	0.970
		Weighted mode	13	0.950	0.780	1.157	0.621
		MR Egger	7	1.001	0.715	1.402	0.995
		Weighted median	7	0.996	0.899	1.103	0.936
Squamous cell lung carcinoma	Phosphatidylcholine (16:0_20:4) levels	Inverse variance weighted	7	0.984	0.883	1.098	0.776
		Simple mode	7	1.101	0.917	1.320	0.342
		Weighted mode	7	1.023	0.895	1.168	0.754
		MR Egger	7	1.045	0.775	1.409	0.783
		Weighted median	7	0.921	0.835	1.015	0.096
Squamous cell lung carcinoma	Phosphatidylcholine (17:0_18:1) levels	Inverse variance weighted	7	0.945	0.855	1.045	0.273
		Simple mode	7	0.920	0.799	1.060	0.294
		Weighted mode	7	0.922	0.820	1.036	0.219
		MR Egger	7	0.957	0.675	1.358	0.817
		Weighted median	7	1.013	0.903	1.137	0.823
Squamous cell lung carcinoma	Phosphatidylcholine (18:0_20:2) levels	Inverse variance weighted	7	0.998	0.889	1.119	0.967
		Simple mode	7	1.014	0.835	1.231	0.892
		Weighted mode	7	1.023	0.881	1.188	0.779
		MR Egger	7	1.012	0.742	1.379	0.943

		Weighted median	7	0.973	0.881	1.074	0.585
Squamous cell lung carcinoma	Phosphatidylcholine (18:0_20:4) levels	Inverse variance weighted	7	0.974	0.881	1.077	0.609
		Simple mode	7	1.031	0.862	1.235	0.747
		Weighted mode	7	0.995	0.880	1.126	0.941
		MR Egger	7	0.920	0.710	1.193	0.559
		Weighted median	7	0.973	0.879	1.076	0.590
Squamous cell lung carcinoma	Phosphatidylcholine (18:1_18:1) levels	Inverse variance weighted	7	1.001	0.917	1.093	0.984
		Simple mode	7	1.112	0.923	1.339	0.309
		Weighted mode	7	0.955	0.836	1.091	0.527
		MR Egger	7	1.056	0.854	1.307	0.636
		Weighted median	7	0.964	0.878	1.058	0.437
Squamous cell lung carcinoma	Phosphatidylcholine (O-16:0_18:1) levels	Inverse variance weighted	7	0.986	0.914	1.063	0.707
		Simple mode	7	0.953	0.833	1.089	0.505
		Weighted mode	7	0.962	0.861	1.076	0.525
		MR Egger	7	1.053	0.849	1.305	0.660
		Weighted median	7	0.988	0.896	1.089	0.809
Squamous cell lung carcinoma	Phosphatidylcholine (O-16:1_18:0) levels	Inverse variance weighted	7	1.003	0.929	1.082	0.946
		Simple mode	7	0.984	0.852	1.137	0.833
		Weighted mode	7	0.972	0.851	1.112	0.696
		MR Egger	7	1.166	0.879	1.547	0.335
		Weighted median	7	0.990	0.887	1.104	0.850
Squamous cell lung carcinoma	Phosphatidylcholine (O-16:1_20:4) levels	Inverse variance weighted	7	1.051	0.954	1.159	0.313
		Simple mode	7	0.973	0.809	1.171	0.780
		Weighted mode	7	0.968	0.845	1.110	0.659
		MR Egger	7	1.038	0.801	1.345	0.790
		Weighted median	7	1.017	0.913	1.133	0.758
Squamous cell lung carcinoma	Phosphatidylcholine (O-18:2_20:4) levels	Inverse variance weighted	7	1.004	0.923	1.092	0.923
		Simple mode	7	0.998	0.837	1.189	0.980
		Weighted mode	7	1.024	0.895	1.172	0.738

		MR Egger	7	0.804	0.538	1.201	0.335
		Weighted median	7	0.990	0.894	1.097	0.846
Squamous cell lung carcinoma	Phosphatidylethanolamine (18:0_18:2)	Inverse variance weighted	7	0.989	0.856	1.142	0.880
		Simple mode	7	1.006	0.854	1.185	0.944
		Weighted mode	7	0.985	0.879	1.104	0.803
		MR Egger	7	1.201	0.903	1.596	0.264
		Weighted median	7	1.041	0.939	1.153	0.449
Squamous cell lung carcinoma	Phosphatidylethanolamine (O-18:1_20:4)	Inverse variance weighted	7	1.072	0.972	1.182	0.165
		Simple mode	7	1.041	0.874	1.240	0.666
		Weighted mode	7	1.037	0.919	1.170	0.575
		MR Egger	7	0.975	0.736	1.290	0.864
		Weighted median	7	0.981	0.883	1.089	0.714
Squamous cell lung carcinoma	Sphingomyelin (d34:0) levels	Inverse variance weighted	7	0.985	0.899	1.080	0.750
		Simple mode	7	0.988	0.840	1.162	0.888
		Weighted mode	7	0.981	0.864	1.115	0.779
		MR Egger	7	1.003	0.741	1.358	0.986
		Weighted median	7	1.086	0.979	1.205	0.118
Squamous cell lung carcinoma	Sphingomyelin (d36:1) levels	Inverse variance weighted	7	1.031	0.935	1.137	0.543
		Simple mode	7	1.118	0.923	1.354	0.297
		Weighted mode	7	1.107	0.966	1.269	0.195
		MR Egger	7	0.890	0.692	1.145	0.406
		Weighted median	7	1.018	0.923	1.122	0.725
Squamous cell lung carcinoma	Sphingomyelin (d38:2) levels	Inverse variance weighted	7	0.999	0.914	1.091	0.980
		Simple mode	7	1.033	0.887	1.204	0.691
		Weighted mode	7	1.016	0.894	1.154	0.817
Small cell lung carcinoma	Phosphatidylcholine (O-16:0_20:3) levels	Inverse variance weighted	2	1.045	0.856	1.276	0.664
Small cell lung carcinoma	Phosphatidylcholine (O-18:0_14:0) levels	Inverse variance weighted	2	0.957	0.848	1.080	0.478
Small cell lung carcinoma	Phosphatidylinositol (18:1_20:4) levels	Inverse variance weighted	2	0.969	0.731	1.284	0.824
Small cell lung carcinoma	Sphingomyelin (d36:2) levels	Inverse variance weighted	2	1.089	0.981	1.209	0.109

Abbreviations: SNPs: single nucleotide polymorphisms.

Supplementary table S9. Using different methods to evaluation the heterogeneity and pleiotropy of our MR analysis.

Exposure	Outcome	No of SNPs	Heterogeneity		Pleiotropy			MR-PRESSO	
			Cochran's Q statistic[1]	P-value	MR-Egger intercept[2]	SE	P-value	Global Test[3]	P-value
Sterol ester (27:1/20:2) levels	Lung carcinoma	25	30.291	0.175	-0.0064	0.0094	0.501	32.098	0.218
Diacylglycerol (16:1_18:1) levels	Lung carcinoma	16	13.336	0.576	-0.0041	0.0122	0.743	14.917	0.614
Phosphatidylcholine (16:0_20:5) levels	Lung carcinoma	20	12.555	0.861	-0.0049	0.0068	0.485	14.242	0.872
Phosphatidylcholine (O-16:1_18:0) levels	Lung carcinoma	16	13.009	0.602	-0.0075	0.0084	0.383	14.811	0.619
Phosphatidylcholine (18:0_20:2) levels	Lung carcinoma	15	11.970	0.609	0.0013	0.0086	0.883	14.958	0.563
Sphingomyelin (d36:2) levels	Lung carcinoma	20	28.096	0.082	0.0120	0.0114	0.303	31.618	0.106
Phosphatidylcholine (18:1_18:2) levels	Lung carcinoma	16	14.297	0.503	0.0108	0.0076	0.179	20.971	0.436
Phosphatidylcholine (O-18:2_20:4) levels	Lung carcinoma	19	20.021	0.332	-0.0066	0.0092	0.484	22.675	0.330
Phosphatidylcholine (16:0_20:4) levels	Lung carcinoma	21	19.487	0.490	-0.0052	0.0059	0.385	24.371	0.520
Phosphatidylcholine (O-16:1_20:4) levels	Lung carcinoma	20	33.944	0.019	-0.0046	0.0096	0.637	36.765	0.043
Phosphatidylcholine (O-18:1_20:3) levels	Lung carcinoma	20	30.495	0.046	0.0017	0.0123	0.889	34.983	0.053

Phosphatidylcholine (17:0_20:4) levels	Lung carcinoma	27	20.039	0.790	-0.0080	0.0049	0.113	30.370	0.591
Triacylglycerol (48:0) levels	Lung adenocarcinoma	15	12.120	0.597	-0.0047	0.0145	0.751	13.612	0.615
Phosphatidylcholine (16:0_20:5) levels	Lung adenocarcinoma	22	9.767	0.982	-0.0064	0.0086	0.467	10.429	0.986
Triacylglycerol (58:7) levels	Lung adenocarcinoma	20	30.390	0.047	-0.0088	0.0234	0.712	33.761	0.052
Phosphatidylcholine (18:0_20:2) levels	Lung adenocarcinoma	16	13.883	0.534	0.0032	0.0116	0.783	15.615	0.595
Diacylglycerol (16:0_18:2) levels	Lung adenocarcinoma	25	37.612	0.038	0.0115	0.0117	0.337	40.026	0.052
Phosphatidylcholine (O-16:1_20:3) levels	Lung adenocarcinoma	21	23.653	0.258	-0.0324	0.0142	0.035	26.231	0.285
Phosphatidylcholine (16:0_20:2) levels	Lung adenocarcinoma	23	10.306	0.983	-0.0035	0.0097	0.727	11.280	0.987
Phosphatidylethanolamine (O-18:2_18:1)	Lung adenocarcinoma	20	15.137	0.714	-0.0130	0.0119	0.290	16.644	0.731
Sterol ester (27:1/20:2) levels	Lung adenocarcinoma	25	18.062	0.800	-0.0002	0.0114	0.985	19.159	0.841
Diacylglycerol (16:1_18:1) levels	Lung adenocarcinoma	15	6.705	0.945	0.0028	0.0170	0.871	7.670	0.957
Phosphatidylethanolamine (O-18:1_20:4)	Squamous cell lung carcinoma	24	16.751	0.821	0.0006	0.0125	0.964	17.813	0.866
Phosphatidylcholine (18:0_20:2) levels	Squamous cell lung carcinoma	16	5.978	0.980	-0.0028	0.0134	0.838	6.569	0.987
Phosphatidylethanolamine (18:0_18:2)	Squamous cell lung carcinoma	25	22.385	0.556	-0.0032	0.0112	0.778	24.092	0.621
Phosphatidylcholine (O-16:1_18:0) levels	Squamous cell lung carcinoma	16	11.656	0.705	-0.0035	0.0130	0.790	13.060	0.719
Phosphatidylcholine (O-16:1_20:4) levels	Squamous cell lung carcinoma	20	13.441	0.815	-0.0099	0.0113	0.389	14.236	0.841

Phosphatidylcholine (16:0_20:4) levels	Squamous cell lung carcinoma	22	17.984	0.650	-0.0011	0.0093	0.905	18.379	0.745
Sphingomyelin (d38:2) levels	Squamous cell lung carcinoma	22	20.402	0.496	0.0178	0.0161	0.281	22.977	0.489
Sphingomyelin (d34:0) levels	Squamous cell lung carcinoma	22	30.236	0.087	0.0085	0.0150	0.575	32.761	0.091
Phosphatidylcholine (18:1_18:1) levels	Squamous cell lung carcinoma	21	24.591	0.218	0.0058	0.0172	0.741	27.156	0.244
Phosphatidylcholine (17:0_18:1) levels	Squamous cell lung carcinoma	16	10.461	0.790	0.0145	0.0129	0.281	11.567	0.818
Phosphatidylcholine (18:0_20:4) levels	Squamous cell lung carcinoma	24	14.798	0.902	-0.0099	0.0072	0.184	24.872	0.697
Phosphatidylcholine (O-16:0_18:1) levels	Squamous cell lung carcinoma	19	15.214	0.647	-0.0134	0.0168	0.436	16.880	0.658
Phosphatidylcholine (O-18:2_20:4) levels	Squamous cell lung carcinoma	19	13.047	0.789	-0.0117	0.0138	0.408	14.762	0.795
Sphingomyelin (d36:1) levels	Squamous cell lung carcinoma	26	37.942	0.047	0.0278	0.0191	0.159	41.230	0.043
Phosphatidylinositol (18:1_20:4) levels	Small cell lung carcinoma	21	17.068	0.649	-0.0252	0.0225	0.277	18.841	0.677
Sphingomyelin (d36:2) levels	Small cell lung carcinoma	17	16.454	0.422	0.0125	0.0299	0.683	18.823	0.438
Phosphatidylcholine (O-16:0_20:3) levels	Small cell lung carcinoma	16	13.896	0.533	-0.0191	0.0219	0.397	15.779	0.591
Phosphatidylcholine (O-18:0_14:0) levels	Small cell lung carcinoma	15	4.568	0.991	-0.0007	0.0240	0.977	5.278	0.993
Sphingomyelin (d36:2) levels	Protein S100-A12 levels	24	23.160	0.451	0.0023	0.0084	0.787	25.236	0.469
Phosphatidylcholine (18:1_18:2) levels	Protein S100-A12 levels	22	17.462	0.683	-0.0098	0.0062	0.129	18.609	0.754
Phosphatidylcholine (18:1_18:2) levels	TNF-beta levels	22	23.874	0.299	-0.0055	0.0076	0.473	25.724	0.381

Phosphatidylcholine (16:0_20:4) levels	Interleukin-18 levels	24	13.817	0.932	0.0006	0.0057	0.920	14.409	0.959
Phosphatidylcholine (16:0_20:4) levels	TNF-beta levels	23	16.394	0.796	-0.0155	0.0075	0.051	17.886	0.795
Phosphatidylcholine (O-18:1_20:3) levels	C-X-C motif chemokine 10 levels	20	15.714	0.676	0.0011	0.0088	0.903	17.818	0.671
Triacylglycerol (58:7) levels	C-C motif chemokine 25 levels	22	21.902	0.405	-0.0144	0.0103	0.177	23.549	0.452
Phosphatidylcholine (18:0_20:2) levels	Interleukin-7 levels	16	17.001	0.319	-0.0129	0.0086	0.154	23.106	0.227
Phosphatidylcholine (16:0_20:4) levels	C-C motif chemokine 4 levels	24	24.319	0.386	-0.0019	0.0060	0.754	25.810	0.509
Phosphatidylcholine (16:0_20:4) levels	Delta and Notch-like epidermal growth factor-related receptor levels	24	17.877	0.764	-0.0017	0.0055	0.760	18.358	0.857
Phosphatidylcholine (16:0_20:4) levels	Interleukin-18 levels	24	13.817	0.932	0.0006	0.0057	0.920	14.409	0.959
Phosphatidylcholine (16:0_20:4) levels	Interleukin-7 levels	24	25.068	0.347	0.0044	0.0060	0.474	33.266	0.314
Sphingomyelin (d34:0) levels	Interleukin-7 levels	27	19.160	0.830	-0.0037	0.0066	0.572	20.373	0.857
Phosphatidylcholine (18:0_20:4) levels	Delta and Notch-like epidermal growth factor-related receptor levels	27	22.465	0.663	-0.0030	0.0042	0.471	23.117	0.743
Phosphatidylcholine (18:0_20:4) levels	Interleukin-18 levels	27	35.718	0.097	-0.0038	0.0050	0.452	39.854	0.201
Phosphatidylcholine (18:0_20:4) levels	Interleukin-7 levels	27	29.913	0.271	0.0018	0.0047	0.704	31.139	0.376
Phosphatidylcholine (18:0_20:4) levels	Monocyte chemoattractant protein-3 levels	27	27.741	0.371	-0.0021	0.0051	0.685	28.779	0.483
Protein S100-A12 levels	Lung carcinoma	23	17.373	0.742	-0.0021	0.0085	0.807	18.705	0.790
TNF-beta levels	Lung carcinoma	29	22.598	0.753	0.0053	0.0052	0.321	46.163	0.541

Interleukin-18 levels	Lung carcinoma	25	21.312	0.620	-0.0002	0.0076	0.980	22.771	0.699
C-X-C motif chemokine 10 levels	Lung carcinoma	29	48.398	0.010	-0.0025	0.0082	0.763	51.935	0.010
C-C motif chemokine 25 levels	Lung adenocarcinoma	27	36.956	0.075	-0.0030	0.0075	0.695	40.094	0.184
Interleukin-7 levels	Squamous cell lung carcinoma	19	12.303	0.831	0.0041	0.0198	0.838	13.597	0.855
C-C motif chemokine 4 levels	Squamous cell lung carcinoma	28	24.612	0.596	-0.0183	0.0081	0.032	28.401	0.557
Delta and Notch-like epidermal growth factor-related receptor levels	Squamous cell lung carcinoma	24	20.115	0.635	-0.0153	0.0111	0.179	23.134	0.627
Interleukin-18 levels	Squamous cell lung carcinoma	27	20.731	0.756	0.0056	0.0118	0.640	21.723	0.790
Monocyte chemoattractant protein-3 levels	Squamous cell lung carcinoma	26	24.107	0.513	-0.0124	0.0146	0.405	25.780	0.567

[1]The Cochran's Q test is a statistical test for heterogeneity.

[2]The intercept term from the MR-Egger regression method is a statistical test of horizontal pleiotropy.

[3]The MR-PRESSO method detected the existence of outlier IVs that may have horizontal pleiotropy through the global test.

Abbreviations: MR-PRESSO: the Mendelian Randomization Pleiotropy RESidual Sum and Outlier; SNPs: single nucleotide polymorphisms; SE: standard error.

Supplementary table S10. Association of SNPs for plasma lipidome with inflammation proteins using MR with different methods.

Exposure	Outcome	method	nsnp	or	or_lci95	or_uci95	pval
Phosphatidylcholine (18:0_20:2) levels	Interleukin-7 levels	MR Egger	16	1.152	1.029	1.289	0.027
		Weighted median	16	1.123	1.044	1.208	0.002
		Inverse variance weighted	16	1.067	1.010	1.128	0.022

		Simple mode	16	1.119	0.946	1.324	0.211
		Weighted mode	16	1.143	1.054	1.239	0.005
Sphingomyelin (d36:2) levels	Protein S100-A12 levels	MR Egger	24	1.049	0.944	1.166	0.385
		Weighted median	24	1.058	0.983	1.140	0.134
		Inverse variance weighted	24	1.063	1.009	1.119	0.020
		Simple mode	24	1.107	0.972	1.262	0.139
		Weighted mode	24	1.076	0.964	1.199	0.204
Phosphatidylcholine (18:1_18:2) levels*	Protein S100-A12 levels	MR Egger	22	1.004	0.930	1.084	0.914
		Weighted median	22	0.955	0.902	1.011	0.116
		Inverse variance weighted	22	0.954	0.914	0.995	0.029
		Simple mode	22	0.970	0.860	1.094	0.624
		Weighted mode	22	0.961	0.905	1.020	0.203
Phosphatidylcholine (18:1_18:2) levels	TNF-beta levels	MR Egger	22	0.972	0.885	1.067	0.556
		Weighted median	22	0.935	0.875	0.999	0.046
		Inverse variance weighted	22	0.944	0.897	0.993	0.027
		Simple mode	22	0.891	0.773	1.027	0.127

		Weighted mode	22	0.932	0.868	1.000	0.064
Phosphatidylcholine (16:0_20:4) levels	Interleukin-18 levels	MR Egger	24	0.964	0.918	1.013	0.167
		Weighted median	24	0.963	0.927	1.000	0.053
		Inverse variance weighted	24	0.966	0.935	0.999	0.043
		Simple mode	24	0.938	0.838	1.050	0.279
		Weighted mode	24	0.959	0.922	0.998	0.050
Phosphatidylcholine (16:0_20:4) levels	C-C motif chemokine 4 levels	MR Egger	24	0.970	0.920	1.022	0.266
		Weighted median	24	0.969	0.933	1.007	0.104
		Inverse variance weighted	24	0.964	0.931	0.998	0.036
		Simple mode	24	0.968	0.852	1.100	0.621
		Weighted mode	24	0.968	0.932	1.005	0.101
Phosphatidylcholine (16:0_20:4) levels	Delta and Notch-like epidermal growth factor-related receptor levels	MR Egger	24	0.953	0.909	1.000	0.064
		Weighted median	24	0.949	0.913	0.986	0.007
		Inverse variance weighted	24	0.948	0.918	0.979	0.001
		Simple mode	24	0.915	0.816	1.025	0.140
		Weighted mode	24	0.948	0.913	0.984	0.010

		MR Egger	24	0.944	0.896	0.995	0.043
		Weighted median	24	0.941	0.905	0.979	0.002
Phosphatidylcholine (16:0_20:4) levels	Interleukin-7 levels	Inverse variance weighted	24	0.958	0.925	0.992	0.016
		Simple mode	24	0.942	0.829	1.071	0.373
		Weighted mode	24	0.941	0.905	0.978	0.005
		MR Egger	23	1.130	1.038	1.230	0.010
		Weighted median	23	1.067	1.003	1.134	0.039
Phosphatidylcholine (16:0_20:4) levels	TNF-beta levels	Inverse variance weighted	23	1.047	1.001	1.095	0.045
		Simple mode	23	1.122	0.987	1.275	0.091
		Weighted mode	23	1.067	1.006	1.132	0.041
		MR Egger	20	1.055	0.937	1.188	0.387
		Weighted median	20	1.098	1.015	1.189	0.020
Phosphatidylcholine (O-18:1_20:3) levels*	C-X-C motif chemokine 10 levels	Inverse variance weighted	20	1.062	1.006	1.121	0.030
		Simple mode	20	0.978	0.844	1.134	0.773
		Weighted mode	20	1.090	0.994	1.196	0.083
		MR Egger	27	0.970	0.893	1.053	0.469

		Weighted median	27	0.949	0.887	1.016	0.131
Sphingomyelin (d34:0) levels	Interleukin-7 levels	Inverse variance weighted	27	0.950	0.908	0.995	0.030
		Simple mode	27	0.935	0.834	1.049	0.262
		Weighted mode	27	0.953	0.879	1.034	0.257
		MR Egger	27	0.974	0.935	1.015	0.222
		Weighted median	27	0.962	0.931	0.994	0.022
Phosphatidylcholine (18:0_20:4) levels	Delta and Notch-like epidermal growth factor-related receptor levels	Inverse variance weighted	27	0.964	0.936	0.992	0.012
		Simple mode	27	0.918	0.822	1.024	0.137
		Weighted mode	27	0.958	0.926	0.992	0.024
		MR Egger	27	0.973	0.926	1.023	0.292
		Weighted median	27	0.968	0.935	1.002	0.065
Phosphatidylcholine (18:0_20:4) levels	Interleukin-18 levels	Inverse variance weighted	27	0.960	0.927	0.994	0.021
		Simple mode	27	0.901	0.794	1.021	0.115
		Weighted mode	27	0.965	0.931	1.001	0.066
		MR Egger	27	0.946	0.903	0.991	0.026
		Weighted median	27	0.948	0.916	0.982	0.003

Phosphatidylcholine (18:0_20:4) levels	Interleukin-7 levels	Inverse variance weighted	27	0.952	0.922	0.983	0.003
		Simple mode	27	0.948	0.846	1.061	0.359
		Weighted mode	27	0.948	0.915	0.981	0.005
		MR Egger	27	0.962	0.915	1.012	0.143
		Weighted median	27	0.959	0.922	0.998	0.038
Phosphatidylcholine (18:0_20:4) levels*	Monocyte chemoattractant protein-3 levels	Inverse variance weighted	27	0.955	0.922	0.989	0.009
		Simple mode	27	1.082	0.949	1.235	0.250
		Weighted mode	27	0.958	0.919	0.999	0.057

*The result with additional MR method (MR-Egger, weighted median, simple mode, weighted mode) is not consistent with the direction of inverse variance weighted method.

Abbreviations: SNPs: single nucleotide polymorphisms.

Supplementary table S11. Association of SNPs for inflammation proteins with lung carcinoma or subtypes using MR with different methods.

Exposure	Outcome	method	nsnp	or	or_lci95	or_uci95	pval
		MR Egger	23	1.146	0.968	1.357	0.129
		Weighted median	23	1.113	0.985	1.259	0.087
Protein S100-A12 levels	Lung carcinoma	Inverse variance weighted	23	1.125	1.039	1.217	0.003
		Simple mode	23	1.241	1.000	1.541	0.062

		Weighted mode	23	1.127	0.972	1.307	0.127
TNF-beta levels	Lung carcinoma	MR Egger	29	1.023	0.985	1.062	0.248
		Weighted median	29	1.018	0.983	1.054	0.315
		Inverse variance weighted	29	1.033	1.001	1.066	0.044
		Simple mode	29	1.067	0.901	1.265	0.458
		Weighted mode	29	1.019	0.985	1.054	0.295
Interleukin-18 levels	Lung carcinoma	MR Egger	25	0.885	0.777	1.007	0.078
		Weighted median	25	0.852	0.780	0.931	0.000
		Inverse variance weighted	25	0.883	0.829	0.941	0.000
		Simple mode	25	0.847	0.718	1.000	0.061
		Weighted mode	25	0.852	0.779	0.931	0.002
C-X-C motif chemokine 10 levels	Lung carcinoma	MR Egger	29	0.932	0.788	1.103	0.421
		Weighted median	29	0.939	0.841	1.048	0.259
		Inverse variance weighted	29	0.912	0.834	0.997	0.043
		Simple mode	29	0.837	0.698	1.004	0.065
		Weighted mode	29	0.930	0.802	1.079	0.348

		MR Egger	28	1.004	0.908	1.109	0.943
		Weighted median	28	0.978	0.892	1.072	0.633
C-C motif chemokine 4 levels*	Squamous cell lung carcinoma	Inverse variance weighted	28	0.927	0.862	0.996	0.039
		Simple mode	28	0.855	0.658	1.109	0.248
		Weighted mode	28	0.978	0.901	1.062	0.601
		MR Egger	24	0.997	0.791	1.258	0.982
		Weighted median	24	0.954	0.813	1.120	0.565
Delta and Notch-like epidermal growth factor-related receptor levels	Squamous cell lung carcinoma	Inverse variance weighted	24	0.862	0.775	0.957	0.006
		Simple mode	24	0.984	0.720	1.344	0.919
		Weighted mode	24	0.964	0.813	1.143	0.676
		MR Egger	27	0.862	0.702	1.057	0.166
		Weighted median	27	0.895	0.781	1.025	0.109
Interleukin-18 levels	Squamous cell lung carcinoma	Inverse variance weighted	27	0.900	0.814	0.994	0.038
		Simple mode	27	0.881	0.688	1.130	0.328
		Weighted mode	27	0.899	0.780	1.035	0.151
		MR Egger	26	0.976	0.768	1.241	0.844

		Weighted median	26	0.894	0.760	1.052	0.178
Monocyte chemoattractant protein-3 levels	Squamous cell lung carcinoma	Inverse variance weighted	26	0.889	0.800	0.987	0.028
		Simple mode	26	0.987	0.740	1.317	0.930
		Weighted mode	26	0.898	0.733	1.102	0.313
		MR Egger	19	0.799	0.511	1.251	0.341
		Weighted median	19	0.813	0.660	1.001	0.051
Interleukin-7 levels	Squamous cell lung carcinoma	Inverse variance weighted	19	0.836	0.716	0.975	0.023
		Simple mode	19	0.808	0.553	1.180	0.285
		Weighted mode	19	0.805	0.565	1.147	0.245

*The result with additional MR method (MR-Egger, weighted median, simple mode, weighted mode) is not consistent with the direction of inverse variance weighted method.

Abbreviations: SNPs: single nucleotide polymorphisms.

Supplementary table S12. Mediating effects of inflammatory proteins in the causal relationship between plasma lipidome and lung carcinoma or subtypes

Exposure	Mediation	Outcome	Total effect (β)	β1	β2	Mediation effect	Direct effect	Mediation effect/Total effect
Sphingomyelin (d36:2) levels	Protein S100-A12 levels	Lung carcinoma	0.082	0.061	0.117	0.007	0.075	0.087
Phosphatidylcholine (18:1_18:2) levels	TNF-beta levels	Lung carcinoma	-0.053	-0.058	0.033	-0.002	-0.051	0.035

Phosphatidylcholine (16:0_20:4) levels	Interleukin-18 levels	Lung carcinoma	0.044	-0.034	-0.124	0.004	0.040	0.096
Phosphatidylcholine (16:0_20:4) levels	TNF-beta levels	Lung carcinoma	0.061	0.046	0.033	0.001	0.060	0.024
Phosphatidylcholine (18:0_20:2) levels	Interleukin-7 levels	Squamous cell lung carcinoma	-0.126	0.065	-0.180	-0.012	-0.115	0.092
Phosphatidylcholine (16:0_20:4) levels	Delta and Notch-like epidermal growth factor-related receptor levels	Squamous cell lung carcinoma	0.068	-0.053	-0.149	0.008	0.060	0.117
Phosphatidylcholine (16:0_20:4) levels	Interleukin-18 levels	Squamous cell lung carcinoma	0.068	-0.034	-0.106	0.004	0.064	0.053
Phosphatidylcholine (16:0_20:4) levels	Interleukin-7 levels	Squamous cell lung carcinoma	0.068	-0.043	-0.180	0.008	0.060	0.113
Sphingomyelin (d34:0) levels	Interleukin-7 levels	Squamous cell lung carcinoma	0.129	-0.051	-0.180	0.009	0.120	0.071
Phosphatidylcholine (18:0_20:4) levels	Delta and Notch-like epidermal growth factor-related receptor levels	Squamous cell lung carcinoma	0.054	-0.037	-0.149	0.006	0.048	0.102
Phosphatidylcholine (18:0_20:4) levels	Interleukin-18 levels	Squamous cell lung carcinoma	0.054	-0.041	-0.106	0.004	0.050	0.080
Phosphatidylcholine (18:0_20:4) levels	Interleukin-7 levels	Squamous cell lung carcinoma	0.054	-0.049	-0.180	0.009	0.045	0.164

β1: The effect of lipids on inflammatory proteins is denoted as β1; β2 The effect of inflammatory proteins on lung carcinoma or subtypes is denoted as β2.
